# Yttrium-90 Selective Internal Radiation Therapy for Neuroendocrine Liver Metastases: An Institutional Case Series, Updated Systematic Review, and Meta-Analysis

**DOI:** 10.3390/diagnostics16010111

**Published:** 2025-12-29

**Authors:** Xinlin Zheng, Fang Wen, Huan Xi, Joyce van Sluis, Reinoud P. H. Bokkers, Susanne Lütje, G. Matthijs Kater, Frederik J. H. Hoogwater, Annemiek M. E. Walenkamp, Derk-Jan A. de Groot, Simeon J. S. Ruiter, Walter Noordzij

**Affiliations:** 1 Department of Nuclear Medicine and Molecular Imaging, University Medical Center Groningen, University of Groningen, 9713 GZ Groningen, The Netherlands; x.zheng02@umcg.nl (X.Z.); h.xi@umcg.nl (H.X.);; 2 Department of Nuclear Medicine, RWTH Aachen University Hospital, 52074 Aachen, Germany; fwen@ukaachen.de (F.W.); sluetje@ukaachen.de (S.L.); 3 Department of Radiology, University Medical Center Groningen, University of Groningen, 9713 GZ Groningen, The Netherlands; 4 Department of Hepato-Pancreato-Biliary Surgery and Liver Transplantation, University Medical Center Groningen, University of Groningen, 9713 GZ Groningen, The Netherlands; 5 Department of Medical Oncology, University Medical Center Groningen, University of Groningen, 9713 GZ Groningen, The Netherlands; a.walenkamp@umcg.nl (A.M.E.W.);

**Keywords:** neuroendocrine liver metastases, radioembolization, survival outcomes, safety, symptom improvement, meta-analysis

## Abstract

**Background/Objectives**: To evaluate the efficacy, safety, and symptom impact of yttrium-90 selective internal radiation therapy (Y-90 SIRT) for neuroendocrine liver metastases (NELM) through an updated systematic review and meta-analysis integrated with our institutional data. **Methods**: We retrospectively analyzed 12 patients with NELM treated with Y-90 resin microspheres between 2019 and 2024. Outcomes included overall survival (OS), hepatic progression-free survival (HPFS), symptom improvement, and adverse events. Concurrently, a systematic review and meta-analysis of 43 studies (including our institutional cohort; total *n* = 2221; 2008–2025) was conducted following PRISMA guidelines. Pooled estimates for survival, tumor response, symptom improvement, and adverse events were derived using random- or fixed-effects models, with publication bias assessed by standard methods. **Results**: In our cohort, the median OS and HPFS were 33.3 and 15.3 months; 71.4% of symptomatic patients reported improvement, with no grade ≥ 3 toxicities. In the meta-analysis, pooled OS rates were 82%, 66%, 52%, and 34% at 1, 2, 3, and 5 years, and HPFS rates were 64%, 41%, and 29% at 1, 2, and 3 years. The pooled objective response rate (ORR) and disease control rate (DCR) were 40% and 87% by RECIST, and 56% and 91% by mRECIST. Patients treated with resin microspheres (ORR 38%, DCR 86%) and glass microspheres (ORR 39%, DCR 83%) showed comparable responses. Symptom improvement was observed in 77% of symptomatic patients, while reported grade ≥ 3 toxicities for individual adverse events were each below 5%. **Conclusions**: Y-90 SIRT is associated with promising survival outcomes, high disease control rates, and substantial symptom improvement in NELM with acceptable toxicity, suggesting its potential value as a liver-directed therapy option.

## 1. Introduction

Neuroendocrine tumors (NETs) are a heterogeneous and increasingly prevalent group of neoplasms, most commonly arising in the gastroenteropancreatic (GEP) tract or bronchopulmonary system [1]. Although many NETs exhibit indolent growth, they often present with distant metastases due to delayed diagnosis. The liver is the most common metastatic site, with up to 78% of patients developing neuroendocrine liver metastases (NELM), which significantly impact prognosis and quality of life [2,3].

NELMs are typically multifocal and diffusely distributed, rendering most patients ineligible for curative resection or local ablation; only 10–25% present with resectable disease [4]. Moreover, systemic therapies generally achieve limited tumor response rates, with outcomes strongly influenced by tumor grade, burden, and patient-related factors, as highlighted in the PROMID and CLARINET trials [5,6]. Given these limitations, attention has increasingly shifted toward locoregional strategies. In particular, the hypervascular nature and predominant hepatic arterial supply of NELMs make them ideal candidates for intra-arterial liver-directed therapies [7]. Both the European Society for Medical Oncology (ESMO) and the European Neuroendocrine Tumor Society (ENETS) recommend such approaches in patients with high tumor burden, particularly for cytoreduction or following systemic therapy failure [4,8].

Among liver-directed approaches, selective internal radiation therapy (SIRT) using Yttrium-90 (Y-90) microspheres has emerged as a promising modality [9,10]. By delivering high-dose β-radiation selectively through tumor-feeding arteries, Y-90 microspheres lodge in the tumor microvasculature where emitted β-particles with limited tissue penetration induce DNA damage and vascular injury, enabling targeted tumor necrosis with minimal damage to surrounding liver tissue [11]. Y-90 SIRT has shown encouraging outcomes in tumor control, relief of hormone-related symptoms, and progression-free survival (PFS), with an acceptable safety profile [12,13,14,15].

Despite growing clinical use, current evidence for Y-90 SIRT in NELM is fragmented across small, retrospective studies with heterogeneous endpoints, lacking comprehensive synthesis to guide clinical practice. Evidence-based reference outcomes are needed to optimize treatment planning, patient selection, and sequencing decisions. This study provides an updated and comprehensive systematic review and meta-analysis, supplemented by institutional data, to establish benchmark outcomes and clarify the clinical role of Y-90 SIRT in the era of precision radionuclide therapy.

## 2. Materials and Methods

### 2.1. Institutional Case Series

#### 2.1.1. Study Design and Patients

We conducted a retrospective analysis, including patients with histologically confirmed NELM who received resin-based Y-90 SIRT in our institution from January 2019 to January 2024.

Demographic and tumor-related variables—including age, sex, Ki-67 index, liver tumor burden, extrahepatic disease, and hormone-related symptoms—were extracted from medical records. Biochemical markers (chromogranin A, serotonin, tryptophan, and 5-hydroxyindoleacetic acid) were also recorded. Liver tumor burden was visually estimated from contrast-enhanced imaging and categorized as <10%, 10–25%, or 25–50%. Tumor distribution was classified as solitary or multifocal. The study was approved by the institutional review board of the University Medical Center Groningen, and the need for informed consent was waived in accordance with the Dutch Act on Medical Scientific Research involving Human Beings (WMO).

#### 2.1.2. SIRT Procedure

The SIRT procedure followed our previously reported protocol [16], including 99mTc-MAA SPECT/CT simulation, selective hepatic arterial administration of Y-90 resin microspheres (SIR-Spheres^®^ ; Sirtex Medical Pty Ltd., St Leonards, NSW, Australia), and prophylactic embolization as needed. All patients received periprocedural octreotide infusion to prevent potential carcinoid crises and proton pump inhibitor for four weeks post-SIRT to prevent potential gastrointestinal side effects.

#### 2.1.3. Outcome Measures and Definitions

Primary outcomes included hepatic progression-free survival (HPFS), overall survival (OS), and progression-free survival (PFS); secondary outcomes included symptom improvement and treatment-related adverse events.

HPFS was defined as the time from treatment to radiologic progression within the liver, death, or last imaging follow-up. OS was measured from the first Y-90 SIRT session to death or last follow-up, and PFS from treatment to progression at any site or death.

Symptom improvement was defined as clinician- or patient-reported relief of endocrine-related symptoms (e.g., flushing, diarrhea, wheezing, fatigue) during the follow-up period. Adverse events were graded according to the Common Terminology Criteria for Adverse Events (CTCAE), version 5.0.

### 2.2. Systematic Review and Meta-Analysis

#### 2.2.1. Literature Search and Study Selection

A systematic search of PubMed and Embase was conducted from 1 January 2008 to 31 May 2025, following PRISMA 2020 guidelines and the Cochrane Handbook for Systematic Reviews of Interventions [17]. The PRISMA 2020 checklist is provided in Supplementary Table S1. The protocol was prospectively registered in PROSPERO (CRD420251129021).

Search terms included combinations of “neuroendocrine tumor,” “NET,” “liver metastases,” “neuroendocrine liver metastasis,” “NELM,” “radioembolization,” “transarterial radioembolization,” “TARE,” “yttrium-90,” and “selective internal radiation therapy” or “SIRT.” The detailed search strategy is provided in Supplementary Table S2. Additional articles were identified by manual screening of reference lists. No language restrictions were applied during the literature search. Non-English studies were screened based on English abstracts, and full texts were assessed when sufficient information was available.

Studies were considered eligible if they: (1) enrolled patients with histologically confirmed NELM; (2) involved treatment with Y-90 TARE or SIRT; and (3) reported at least one relevant clinical outcome (e.g., tumor response, survival, symptom improvement, or adverse events). Exclusion criteria were: (1) reviews, meta-analyses, conference abstracts, or case reports; (2) preclinical (animal or in vitro) studies; (3) studies not focused on NELM; (4) studies lacking relevant outcome data; and (5) duplicate patient cohorts.

#### 2.2.2. Data Extraction and Risk of Bias Assessment

Two independent reviewers screened titles, abstracts, and full texts for inclusion, and extracted data on study characteristics (design, sample size, country), patient demographics, tumor and treatment parameters, outcome definitions, and reported results. Discrepancies were resolved by consensus or adjudication by a third reviewer. Inter-reviewer agreement for study selection was quantified using Cohen’s kappa, with interpretation according to the Landis and Koch criteria.

Risk of bias in non-randomized studies was assessed using the ROBINS-I tool. Domains evaluated included confounding, selection bias, classification of interventions, deviations from intended interventions, missing data, measurement of outcomes, and selection of reported results.

#### 2.2.3. Outcome Measures

The primary and secondary outcomes were categorized into four domains: survival outcomes, tumor response, symptom improvement, and adverse events.

Survival outcomes included HPFS, OS, and PFS, which were defined consistently with the institutional case series. Survival rates were extracted at 1, 2, and 3 years for all endpoints, and additionally at 5 years for OS when available.

Tumor response was evaluated according to Response Evaluation Criteria in Solid Tumors (RECIST) or modified RECIST (mRECIST) criteria, as reported in the original studies. Objective response rate (ORR) was defined as the proportion of patients achieving complete or partial response, and disease control rate (DCR) as the proportion achieving complete response, partial response, or stable disease. Response data were extracted at the post-treatment imaging follow-up, typically 3–6 months after Y-90 SIRT. Given the availability of data and clinical relevance, a subgroup analysis of tumor response was conducted according to microsphere type (resin-based vs. glass-based), based on RECIST evaluation.

Symptom improvement was extracted as reported in the original studies. Adverse events were assessed according to the CTCAE, as reported in the original studies, and were stratified by type and severity grade. For each outcome, studies were included in the corresponding synthesis if they reported sufficient data for that specific endpoint; studies could contribute to more than one synthesis depending on the outcomes reported.

### 2.3. Statistical Methods

Institutional data were analyzed using STATA version 15.0 (StataCorp, College Station, TX, USA). Continuous variables were summarized as medians with interquartile ranges (IQRs); categorical variables as counts and percentages. Survival analyses (HPFS, OS, and PFS) were performed using the Kaplan–Meier method.

For the meta-analysis, all outcomes were synthesized using rate-based effect measures, including survival rates for survival outcomes and proportions for tumor response, symptom improvement, and adverse events. Pooled estimates were calculated using a random-effects model (DerSimonian and Laird) when heterogeneity was significant (I^2^ > 50%) and a fixed-effects model otherwise. Proportions were transformed using the Freeman–Tukey double arcsine method. Heterogeneity was assessed using Cochran’s Q and the I^2^ statistic. Results of individual studies and pooled estimates were presented using forest plots and summary tables. Publication bias was evaluated using funnel plots and Egger’s test (for ≥10 studies). Where applicable, the trim-and-fill method was applied. Leave-one-out sensitivity analyses were performed to test robustness. All meta-analyses were conducted in R version 4.4.1 (R Foundation for Statistical Computing, Vienna, Austria).

## 3. Results

### 3.1. Results of the Institutional Case Series

#### 3.1.1. Patient Characteristics

Twelve patients with NELM underwent Y-90 resin microsphere SIRT at our institution (Table 1). Median age was 65 years (IQR: 60–68), and 67% were male. Most had multifocal liver metastases (92%) and 42% had extrahepatic disease. Liver tumor burden was <25% in 58% of patients and 25–50% in 42%.

Most tumors were Grade 1 or 2 (42% each), with a median Ki-67 index of 10% (IQR: 3.5–17%). Primary tumors arose from the gastrointestinal tract (50%), pancreas (25%), or lung (8%), with two unknown sites. Half of the patients had prior primary tumor resection.

Hormone-related symptoms were reported in 7 of 12 patients (58%). Serotonin-secreting NETs were identified in 8 of 12 patients (66.7%).

#### 3.1.2. Treatment Parameters

Table 2 summarizes the treatment-related details. Seven patients (58%) received sequential lobar treatment, while three (25%) underwent single-lobar therapy and two (17%) received selective segmental infusion. The median pulmonary shunt fraction was 6.6% (IQR: 4.0–11%). The median administered activity of Y-90 was 1.5 GBq (IQR: 1.2–1.7). The median tumor dose was 160 Gy (IQR: 120–365), and the non-tumoral liver dose was 40 Gy (IQR: 30–45). The median treated liver volume was 1400 mL (IQR: 625–1500).

Pre-SIRT treatment included octreotide (83%), lanreotide (17%), everolimus (8.3%), PRRT (8.3%), thermal ablation (8.3%), and chemotherapy (8.3%). One patient was treatment-naïve. Post-SIRT, 75% of patients resumed octreotide.

#### 3.1.3. Clinical Outcomes

**Figure 1:**
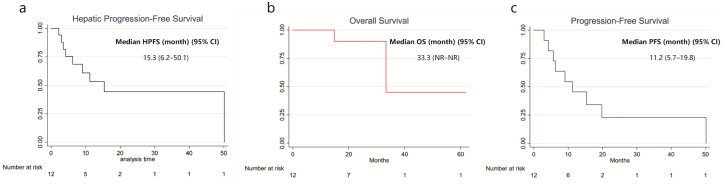
Kaplan–Meier Curve for Survival in Patients Receiving Y-90 SIRT with NELM. (**a**) Hepatic Progression-free survival; (**b**) Overall survival; (**c**) Progression-free survival.

**Figure 2:**
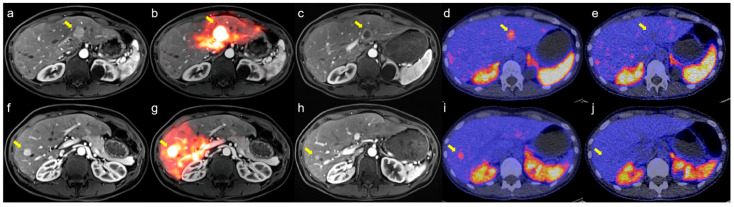
Representative case demonstrating treatment response after sequential bilobar Y-90 SIRT for neuroendocrine liver metastases (NELM). (**a**,**f**) Baseline contrast-enhanced MRI before SIRT showing arterial-enhancing metastases in segment 2 (**a**, arrow) and segment 8 (**f**, arrow); (**d**,**i**) Baseline [^68^Ga]Ga-DOTATOC PET/CT showing intense somatostatin receptor (SSTR) uptake corresponding to the MRI-visible lesions (arrows); (**b**,**g**) The patient subsequently underwent sequential bilobar Y-90 SIRT, with arrows indicating the treated lesions; (**c**,**h**) Four-month follow-up MRI demonstrates reduction in lesion size and decreased arterial enhancement in the treated metastases (arrows), consistent with treatment response; (**e**,**j**) Follow-up [^68^Ga]Ga-DOTATOC PET/CT shows markedly reduced SSTR uptake in treated lesions (arrows), with no evidence of new SSTR-avid disease.

With a median follow-up of 26 months, the median HPFS was 15.3 months (95% CI: 6.2–50.1), OS was 33.3 months (95% CI: NR–NR), and PFS was 11.2 months (95% CI: 5.7–19.8). Kaplan–Meier survival curves are presented in Figure 1. Symptom improvement following SIRT was observed in 5 of 7 evaluable patients (71.4%, Table 2). The most frequently reported treatment-related adverse events were abdominal pain (50%), nausea (25%), vomiting (25%), and fatigue (16.7%). Less common events included fever, diarrhea, dyspnea, hyperglycemia, hypertension, and abdominal infection (each in 8.3%), all of which were self-limiting. All adverse events were mild (CTCAE grade 1–2), and no grade ≥ 3 toxicity was observed (Table 3). A representative case example is presented in Figure 2.

### 3.2. Results of the Systematic Review and Meta-Analysis

#### 3.2.1. Study Characteristics

An overview of the included studies is provided in Table 4. A total of 43 studies comprising 2221 patients with NELM treated with Y-90 SIRT were included, including one retrospective study from our institution. The study selection and screening process is detailed in the PRISMA flow diagram (Supplementary Figure S1). Among the 43 studies, 35 were retrospective (26 single-center, 9 multicenter) and 8 were prospective (6 single-center, 2 multicenter). Most studies were conducted in the United States (*n* = 18) and Europe (*n* = 20), with others from Australia, Turkey, and Canada. The median sample size was 42 (range, 6–244).

The primary tumor was most commonly gastrointestinal (median 47%), followed by pancreatic (28%) and pulmonary (7%). Hormone-related symptoms were reported in 15 studies (median prevalence: 44%), and tumor grade or Ki-67 were available in 30 studies using varied classification systems.

Liver tumor burden was reported in 24 studies, with 18 using the <25%, 25–50%, >50% stratification; most patients had <25% involvement. Extrahepatic disease was reported in 28 studies. Treatment details and dosimetric parameters are summarized in Supplementary Table S3.

#### 3.2.2. Risk of Bias Assessment

**Figure 3:**
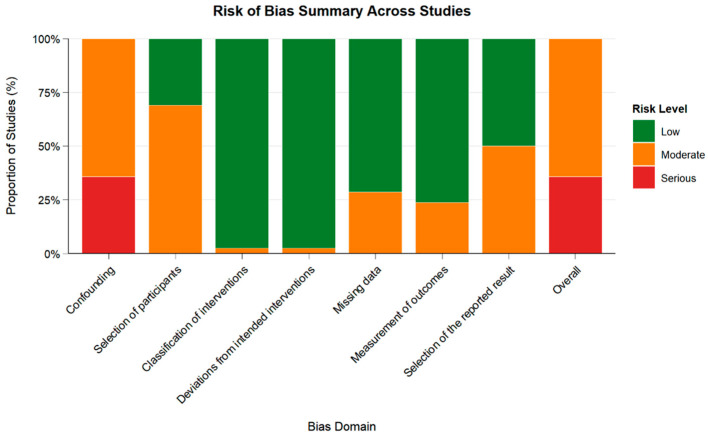
Summary of Risk of Bias Judgments Across Domains Using ROBINS-I. Green: Low risk; Orange: Moderate risk; Red: Serious risk. Risk of bias was assessed for 42 studies using the ROBINS-I tool.

Inter-reviewer agreement was high (κ = 0.78), consistent with substantial agreement. Risk of bias was assessed using the ROBINS-I tool. Of the 42 studies included, 64% were judged to have moderate risk, and 36% had serious risk of bias. The most common concerns were related to confounding and selection of participants. A full breakdown is available in Supplementary Table S4 and Figure 3.

#### 3.2.3. Survival Outcomes

HPFS was reported in 10 studies, with 1-, 2-, and 3-year rates available in 7, 6, and 4 studies, respectively. The pooled HPFS estimates were 64% (95% CI: 58–69%) at 1 year, 41% (95% CI: 35–47%) at 2 years, and 29% (95% CI: 21–37%) at 3 years.

OS was reported in 32 studies. Pooled 1-, 2-, 3-, and 5-year OS rates, based on data from 25, 24, 22, and 9 studies, respectively, were 82% (95% CI: 78–87%), 66% (95% CI: 61–72%), 52% (95% CI: 46–58%), and 34% (95% CI: 25–44%).

PFS data were available in 14 studies, with 1-, 2-, and 3-year rates reported in 6, 6, and 5 studies. The pooled rates were 63% (95% CI: 43–81%), 50% (95% CI: 32–67%), and 35% (95% CI: 19–53%), respectively.

**Figure 4:**
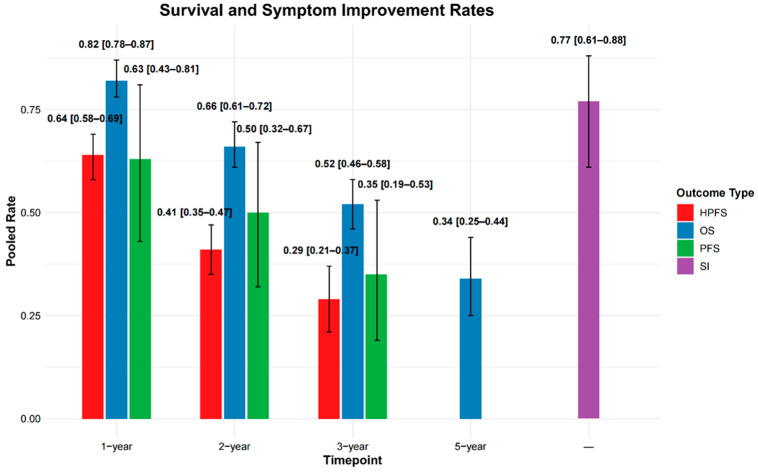
Pooled rates of hepatic progression-free survival (HPFS), overall survival (OS), and progression-free survival (PFS) are shown at 1-, 2-, and 3-year timepoints. OS is additionally reported at 5 years, and symptom improvement (SI) is presented without a specified timepoint. Error bars represent 95% confidence intervals (CIs).

Full study-level data are presented in Supplementary Table S5, with pooled estimates summarized in Supplementary Table S6 and Figure 4.

#### 3.2.4. Tumor Response

Thirty-four studies reported tumor response (Supplementary Table S5), primarily using RECIST criteria (*n* = 29), with three studies reporting mRECIST criteria, and two studies reporting both.

Under RECIST criteria (30–31 studies), the pooled ORR was 40% (95% CI: 32–48%) and disease control rate (DCR) was 87% (95% CI: 83–89%). Complete response (CR), partial response (PR), stable disease (SD), and progressive disease (PD) rates were 5%, 35%, 48%, and 13%, respectively.

Under mRECIST criteria (5 studies), pooled ORR and DCR were 56% (95% CI: 40–70%) and 91% (95% CI: 83–96%), respectively. CR, PR, SD, and PD rates were 8%, 46%, 36%, and 7%.

**Figure 5:**
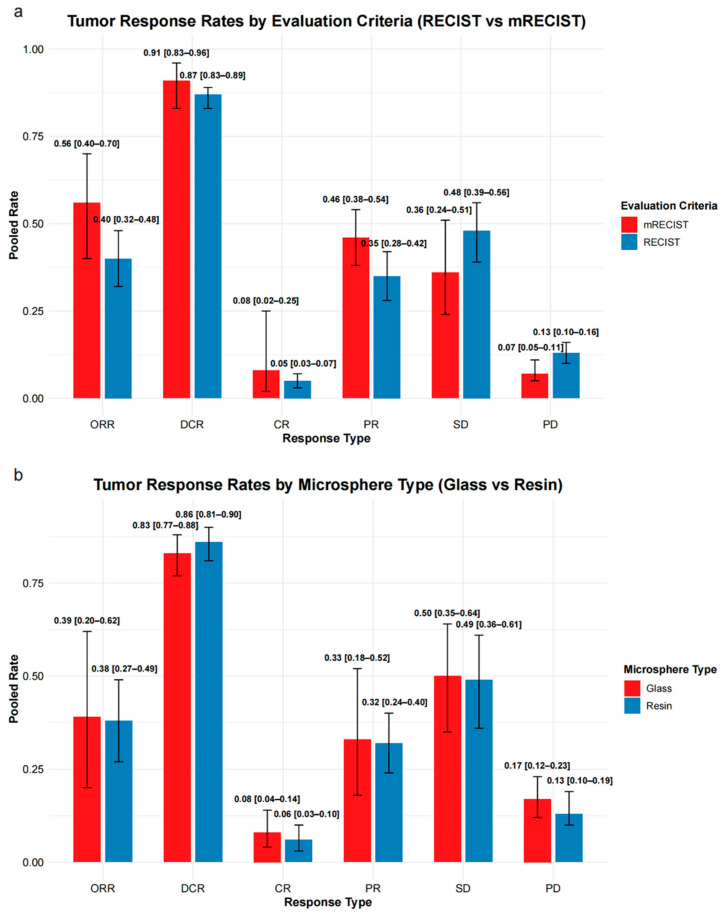
Summary of tumor response rates stratified by evaluation criteria and microsphere type. (**a**) Pooled response rates based on mRECIST and RECIST criteria across six response types: overall response rate (ORR), disease control rate (DCR), complete response (CR), partial response (PR), stable disease (SD), and progressive disease (PD). (**b**) Pooled response rates comparing glass and resin microspheres, evaluated using RECIST-based response categories. Error bars represent 95% confidence intervals (CIs).

Full pooled estimates are presented in Supplementary Table S7 and Figure 5a.

#### 3.2.5. Subgroup Analysis of Tumor Response by Microsphere Type

A subgroup analysis using RECIST criteria was conducted to compare tumor response rates between resin and glass microspheres (Supplementary Table S8 and Figure 5b).

For resin microspheres (17–18 studies), the pooled ORR was 38% (95% CI: 27–49%) and DCR was 86% (95% CI: 81–90%). CR, PR, SD, and PD rates were 6%, 32%, 49%, and 13%, respectively.

For glass microspheres (7 studies), the pooled ORR was 39% (95% CI: 20–62%) and DCR was 83% (95% CI: 77–88%), with CR, PR, SD, and PD rates of 8%, 33%, 50%, and 17%, respectively.

Individual response categories were broadly comparable between groups, with overlapping confidence intervals.

#### 3.2.6. Symptom Improvement

Symptom improvement data were reported in 12 studies (Supplementary Table S5). The pooled symptom improvement rate was 77% (95% CI: 61–88%) (Supplementary Table S6 and Figure 4). Individual study rates varied widely, ranging from 23% (Zuckerman et al., 2019 [45]) to 100% (Filippi et al., 2016 [36]). Four studies reported symptom improvement rates exceeding 90%.

#### 3.2.7. Adverse Events

**Figure 6:**
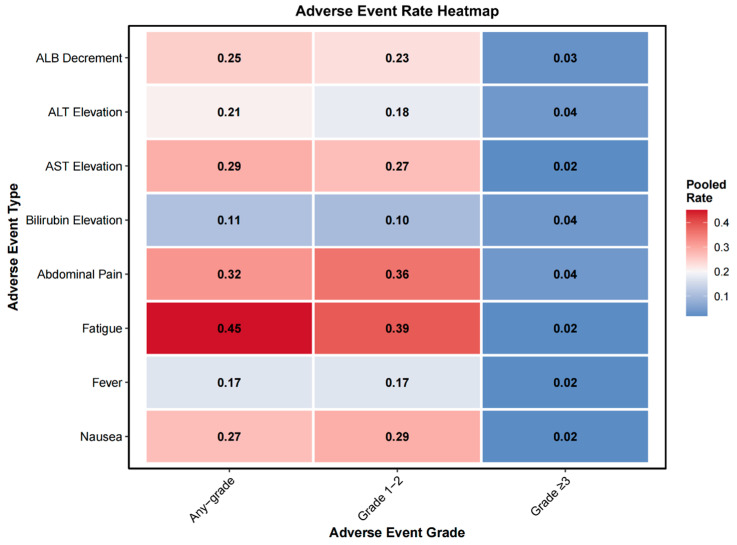
Heatmap of pooled adverse event rates stratified by severity grade. Pooled rates of eight common adverse events are presented across three severity categories: any grade, grade 1–2, and grade 3 or higher. Events include albumin (ALB) decrement, alanine aminotransferase (ALT) elevation, aspartate aminotransferase (AST) elevation, bilirubin elevation, abdominal pain, fatigue, fever, and nausea. Color intensity increases with the pooled rate, with darker red indicating higher event frequencies.

Adverse event data were reported in 4 to 11 studies depending on the toxicity type (Supplementary Table S9). The pooled meta-analytic results are summarized in Supplementary Table S10 and Figure 6. Grade ≥ 3 events were infrequent across all categories. The most commonly reported Grade ≥ 3 toxicities included bilirubin elevation (4%, 95% CI: 2–6%), abdominal pain (4%, 95% CI: 3–6%), and ALT elevation (4%, 95% CI: 2–7%). Other Grade ≥ 3 events occurred in ≤3% of patients, including fatigue (2%, 95% CI: 0–3%), nausea (2%, 95% CI: 1–4%), AST elevation (2%, 95% CI: 1–5%), ALB decrement (3%, 95% CI: 1–7%), and fever (2%, 95% CI: 1–5%).

#### 3.2.8. Assessment of Heterogeneity, Publication Bias, and Robustness

We assessed heterogeneity, publication bias, and robustness of pooled estimates. Heterogeneity varied across endpoints: OS showed persistent moderate between-study heterogeneity across timepoints, HPFS demonstrated low heterogeneity at later timepoints, whereas PFS showed substantial heterogeneity throughout. Funnel plots and Egger’s tests indicated possible small-study effects for DCR, CR, and PD; trim-and-fill analyses suggested a small impact on DCR but a potentially substantial impact on absolute CR and PD estimates. Leave-one-out analyses confirmed that no single study significantly altered pooled outcomes, suggesting that the observed asymmetry was not driven by individual influential studies but may reflect underlying small-study effects or selective reporting. Full results of heterogeneity assessment, publication bias evaluation, and sensitivity analyses are provided in the Supplementary Text and Supplementary Figures S2–S27.

## 4. Discussion

This systematic review and meta-analysis, integrating 43 studies comprising 2221 patients, provides an updated and comprehensive evaluation of Y-90 SIRT in NELM. By systematically assessing tumor response, survival, symptom control, safety, and prognostic factors, this study extends the evidence beyond prior smaller or single-endpoint analyses. The pooled outcomes offer reference values that may help guide clinical decision-making and inform future guideline recommendations.

### 4.1. Clinical Efficacy

Y-90 SIRT is associated with favorable efficacy in patients with NELM. In our meta-analysis, pooled OS rates were 82%, 66%, 52%, and 34% at 1, 2, 3, and 5 years, respectively. Corresponding HPFS rates were 64%, 41%, and 29%, and PFS rates were 63%, 50%, and 35% at 1, 2, and 3 years. The pooled ORR and DCR were 40% and 87% by RECIST, and 56% and 91% by mRECIST, respectively. The pooled symptom improvement rate was 77%, suggesting meaningful clinical benefit, particularly for patients with functional tumors.

Due to the absence of prospective head-to-head trials, the relative efficacy of Y-90 SIRT compared to other liver-directed therapies remains incompletely defined. Yang et al. [59] reviewed 37 studies (1575 patients) and reported 1-, 2-, 3-, and 5-year OS rates of 84.7%, 63%, 46%, and 50.5% for SIRT versus 75%, 66%, 48%, and 30.5% for TACE. The ORR was 63.1% for SIRT versus 58.4% for TACE (RECIST criteria), with symptom improvement rates of 85% and 88.5%, respectively. Kanabar et al. [60] analyzed 101 studies (5545 patients) and reported higher symptom improvement rates for SIRT (77.4%) compared to TACE (47.2%) and TAE (60%). These indirect comparisons suggest comparable survival with potentially superior tumor control and symptom improvement for SIRT.

However, direct comparative studies show mixed results. Egger et al. [47] retrospectively analyzed 248 NELM patients (51 SIRT, 197 TACE). Despite higher tumor aggressiveness in the SIRT group (G2-G3: 39.4% vs. 24.4%, *p* = 0.012) and superior short-term disease control for TACE (96% vs. 83%, *p* < 0.01), median OS (35.9 vs. 50.1 months, *p* = 0.3) and PFS (15.9 vs. 19.9 months, *p* = 0.37) were similar. SIRT demonstrated treatment convenience advantages, with 92% of procedures performed in the outpatient setting compared to 99% of TACE patients requiring at least one overnight hospitalization. Ngo et al. [61] meta-analyzed 28 studies (1713 patients) and found superior OS for TACE (HR 1.92, 95% CI 1.14–3.25, *p* = 0.014), but no difference in hepatic tumor response rates. In functional tumor patients, OS was similar between modalities (HR 1.55, *p* = 0.27).

### 4.2. Safety

Regarding safety, our meta-pooled analysis showed that the most common adverse events were fatigue (45%), abdominal pain (32%), nausea (27%), and elevated AST levels (29%), most of which were grade 1–2 and self-limiting. The pooled incidence of grade ≥ 3 events for each toxicity type was low, with none exceeding 5%. Due to inconsistent reporting across studies, we were unable to calculate an overall pooled incidence of grade ≥ 3 toxicity. A review reported CTCAE grade ≥ 3 toxicity rates ranging from 0% to 12.9% (median = 2.1%) from SIRT, whereas reported rates for TACE ranged from 0% to 25% (median = 5%) [59]. The head-to-head comparison by Egger et al. [47] also found no significant differences between the two modalities in grade III/IV complications (SIRT 5.9% vs. TACE 9.2%, *p* = 0.58), or 90-day mortality.

While short-term toxicity appears acceptable, long-term hepatotoxicity warrants attention in this population. Unlike hepatocellular carcinoma, patients with NELM often have prolonged survival, making late-onset toxicities (e.g., radiation-induced liver injury) clinically relevant, although the absence of cirrhosis in most patients may mitigate the risk of decompensation [15,62]. Several long-term retrospective series have reported persistent biochemical abnormalities and imaging findings consistent with chronic liver injury, including cirrhosis-like morphology and signs of portal hypertension [42,63].

The incidence and severity of these late effects appear to vary by treatment approach and extent. Tomozawa et al. [42] reported that among 52 NET patients with >1-year follow-up after Y-90 resin microsphere treatment, 29% developed cirrhosis-like morphology or imaging evidence of portal hypertension; notably, these changes occurred more frequently in patients receiving bilobar treatment compared to unilobar treatment. Corroborating these findings, Su et al. [63] observed that among NELM patients, whole-liver Y-90 glass microsphere radioembolization resulted in higher rates of cirrhosis-like morphology (56% vs. 27%), ascites (41% vs. 13%), and varices (15% vs. 7%) compared to unilobar treatment at mean 4.1-year follow-up, demonstrating greater radiation-induced hepatic changes with extensive treatment volumes.

When directly compared to TACE, delayed hepatotoxicity occurred in 29% of NET patients treated with Y-90 SIRT compared to 22% with TACE, with SIRT-related toxicities being more severe and manifesting as clinical hepatic decompensation, while TACE toxicities were primarily laboratory derangements [62]. Prospective studies with extended follow-up are needed to further delineate dose–toxicity relationships and late hepatic sequelae in this relatively indolent tumor population.

### 4.3. Prognostic Factors and Patient Stratification

Several key prognostic factors have been identified that influence SIRT outcomes in NELM patients. Tumor burden is one of the significant predictors of survival [64]. Multiple studies have confirmed tumor burden’s prognostic value using different thresholds. Braat et al. found hepatic tumor burden ≥ 75% was associated with significantly worse survival [43], while Saxena et al. reported that low hepatic tumor burden (<25%) was a favorable prognostic factor (*p* = 0.022) [23]. Ingenerf et al. demonstrated through multivariate analysis that hepatic tumor burden > 10% independently predicted worse OS (HR 30.0, *p* = 0.012) and hepatic PFS (HR 11.3, *p* = 0.01) [55]. This suggests that early intervention when liver disease is still limited may improve outcomes.

Treatment timing significantly impacts outcomes. Patients receiving Y-90 SIRT as second-line therapy had median OS of 44.8 months versus 30.6 months in salvage settings (*p* = 0.078) [51]. More importantly, hepatic and global PFS were significantly worse in heavily pretreated patients compared with second-line therapy (*p* = 0.011 and *p* = 0.010) [51], suggesting earlier integration of SIRT may optimize outcomes.

Tumor grade has shown inconsistent prognostic value across studies. In a multicenter cohort of 244 patients, grade was associated with overall survival but not with radiologic response after SIRT, with disease control rates > 90% across G1–G3 tumors despite significant OS differences (3.1, 2.4, and 0.9 years; *p* < 0.001) [43]. In contrast, the RESiN registry (*n* = 170) found no significant differences in OS or PFS by grade [52]. Ingenerf et al. identified patients with Ki-67 > 5% showing significantly longer OS (73.3 vs. 26.1 months, *p* = 0.054) and hepatic PFS (HR 10.6, *p* = 0.02) [55]. Tsang et al. reported that lower Ki-67 (≤2%) was associated with longer survival after Y-90, whereas high Ki-67 (>20%) predicted poorer outcomes [48].

Extrahepatic disease shows conflicting prognostic impact. Some studies identify it as a significant negative factor: Braat et al. reported worse OS (HR 1.7, *p* = 0.04) [43], and Saxena et al. found absence of extrahepatic disease strongly predicted survival (*p* < 0.001) [23]. However, other large studies found no significant impact: Schaarschmidt et al. (297 patients, *p* = 0.335) [51] and the RESiN registry (170 patients with 45% having extrahepatic disease) [52]. When liver disease remains dominant, SIRT may still provide benefit in selected patients with limited extrahepatic disease.

Primary tumor site shows no significant prognostic impact in SIRT-treated patients. The RESiN registry (*n* = 170) found no significant differences in OS or PFS by tumor origin (*p* > 0.1), despite pancreatic primaries showing the longest median OS (42 months) [52]. Peker et al. similarly reported primary NET site was not a significant prognostic factor (*p* = 0.335) [32]. Performance status is a critical prognostic factor: ECOG PS 0 independently predicted better survival (*p* = 0.0001) [26], with ECOG ≥ 2 consistently showing significantly worse OS and PFS [52].

Emerging evidence suggests [^68^Ga]Ga-DOTATATE PET/CT parameters have prognostic value: high baseline tumor SUVmax and high tumor-to-liver SUV ratios are associated with longer PFS and OS after SIRT [55]. Baseline chromogranin A > 1330 ng/mL was associated with shorter hepatic PFS (HR 7.1, *p* = 0.02) but not OS [55].

Delivery technique also impacts outcomes. Radiation segmentectomy—delivering ablative doses (>200 Gy, with median dose of 235.3 Gy) to 1–2 hepatic segments targeting small liver volumes (100–200 mL)—achieved 83% objective response by RECIST with no treated lesion progression and median OS of 69.4 months in NELM patients, demonstrating favorable toxicity (6% Grade 3 events) [58]. This parenchymal-sparing approach may benefit selected patients with oligometastatic liver-dominant disease, particularly those with limited tumor burden. However, most previous studies used simplified dosimetry and lobar administration rather than personalized, selective approaches (Supplementary Table S3). Notably, lesion-level studies demonstrate dose–response relationships with thresholds ≥ 150 Gy for glass and ~190 Gy for resin microspheres [53,65], highlighting the potential value of personalized dosimetry in optimizing SIRT efficacy.

These factors may help inform multidisciplinary patient selection, with optimal candidates likely to have limited tumor burden, G1-G2 histology, good performance status, liver-dominant disease, and earlier treatment line positioning. SIRT is a liver-directed locoregional therapy that does not directly treat extrahepatic disease [66]. Optimal patient selection should prioritize those with liver-limited or liver-dominant disease, where control of hepatic tumor burden is the primary therapeutic objective.

### 4.4. Refining Response Assessment: From RECIST to Whole-Liver Evaluation

Current response assessment following Y-90 SIRT for NELM primarily relies on size-based RECIST or enhancement-based mRECIST criteria. In our meta-analysis, mRECIST demonstrated a numerically higher pooled objective response rate (56%) than RECIST (40%), likely reflecting its greater sensitivity to tumor necrosis—one of the main effects of SIRT.

However, few studies have directly compared or validated the prognostic performance of RECIST versus mRECIST in the setting of Y-90 SIRT for NELM. Most prior investigations on these criteria have focused on HCC or NELM treated with TAE/TACE, where their ability to predict long-term outcomes remains questionable [67]. This limitation is even more relevant in NELM, where liver involvement is typically multifocal and heterogeneous [66]. Both RECIST and mRECIST assess only a few target lesions and fail to capture total viable tumor burden across the liver [67].

Emerging approaches such as liver enhancing tumor burden (LETB), which assess volumetric and enhancement-based metrics across the whole liver, have demonstrated superior prognostic performance compared to RECIST and mRECIST in NELM patients undergoing intra-arterial therapies [68]—though not yet validated for SIRT. These findings support the potential value of whole-liver, mechanism-aligned response assessment criteria that better align with radioembolization effects and support clinical decision-making and outcome prediction.

### 4.5. Comparative Efficacy of Resin and Glass Microspheres

To our knowledge, this is the first meta-analysis to report separately pooled tumor response outcomes for resin and glass microspheres in patients with NELM. Despite distinct physical characteristics and radioactivity delivery profiles [69], subgroup analyses demonstrated comparable tumor response rates: pooled ORR was 0.38 (95% CI: 0.27–0.49) for resin versus 0.39 (95% CI: 0.20–0.62) for glass microspheres, DCR was 0.86 versus 0.83, and CR rates were 0.06 versus 0.08, respectively. These overlapping results suggest no statistically significant difference in tumor response between microsphere types based on available data. However, these findings warrant cautious interpretation given the limited number of glass microsphere studies and lack of long-term outcome data (survival and safety), potentially compromising the strength of comparative conclusions. Microsphere selection should be individualized based on patient characteristics and institutional experience, until evidence of high-quality head-to-head comparisons are available.

### 4.6. Prospects for Combination Therapy and Clinical Translation

Although this study did not include subgroup analyses of combination regimens, accumulating evidence highlights the synergistic potential of Y-90 SIRT with systemic therapies. Soulen et al. [56] reported a median OS of 130 months with a Capecitabine and temozolomide (CapTem) plus SIRT, while Kim et al. [70] observed a median OS of 46.3 months, PFS of 18.6 months, and an ORR of 46% using a combination of octreotide, everolimus, and SIRT. Sequential PRRT followed by Y-90 SIRT has demonstrated promising outcomes, with disease control rates of approximately 91% and median OS of 29–42 months, along with an acceptable safety profile and rare radioembolization-induced liver disease [25,46].

Several agents may enhance the therapeutic impact of Y-90 SIRT through radiosensitization or complementary biological effects. mTOR inhibitors such as everolimus have been shown to increase radiosensitivity in preclinical models by inhibiting radiation-induced angiogenesis and DNA repair [71,72]. Somatostatin analogs (SSAs) have demonstrated antiproliferative effects, reduce proangiogenic signaling, and help control hormone-related symptoms [73,74]. CapTem disrupts DNA synthesis and repair, potentially amplifying the cytotoxic effect of radioembolization [75]. PRRT delivers systemic radiation targeting somatostatin receptors highly expressed on tumor cells, which may complement the locoregional effect of Y-90 SIRT [46].

These combination and sequential strategies provide a flexible approach to managing both liver-dominant and systemic disease. Y-90 SIRT offers strong locoregional control, while selected systemic agents may expand therapeutic reach. Future studies should refine combination regimens, optimize treatment sequencing, and tailor patient selection based on tumor characteristics, disease burden, and predictive biomarkers, with the goal of maximizing efficacy and minimizing toxicity. Although a prior trial combining SIRT with SSA (NCT02859064) was terminated due to slow accrual, other prospective studies—such as SIRT combined with immunotherapy (NCT03457948)—remain ongoing.

### 4.7. Limitations

This study has several limitations that warrant cautious interpretation. First, most included studies were single-arm retrospective series; survival outcomes were synthesized as pooled proportions at fixed timepoints rather than time-to-event analyses and should be interpreted as descriptive benchmarks. Second, most studies had moderate to serious risk of bias. Publication bias affected tumor response outcomes (DCR, CR, PD) but not survival. Trim-and-fill analyses indicated minimal DCR impact but potential CR underestimation (5% to 15%), warranting cautious interpretation. Third, substantial clinical and methodological heterogeneity existed across studies regarding patient characteristics, treatment background, outcome definitions, and reporting standards. The lack of stratified data in previous studies prevented meaningful subgroup analyses (e.g., by disease stage, tumor grade, or primary tumor site), limiting further insight into which patients may benefit most from SIRT. Finally, incomplete reporting and inconsistent follow-up intervals across previous studies may have affected the precision of pooled estimates.

## 5. Conclusions

Y-90 SIRT is associated with promising survival outcomes, high disease control rates, and meaningful symptom improvement in patients with NELM, with an acceptable safety profile. This meta-analysis of 2221 patients across 43 studies provides benchmark outcomes supporting the role of Y-90 SIRT as a liver-directed therapy in NELM. Future studies should focus on comparative effectiveness, optimal treatment sequencing, and combination strategies to further refine its clinical application.

## Figures and Tables

**Table 1:** Baseline characteristics of NELM patients receiving Y-90 SIRT.

Characteristics	Number (%)/Median (IQR)
Patients number	12
Age (y)	65 (60–68)
Sex, No. (%)	
Male	8 (66.7)
Female	4 (33.3)
Extrahepatic Metastasis No. (%)	
Absence	7 (58.3)
Presence	5 (41.7)
Maximum tumor diameter (mm)	66 (53–71)
Pattern of tumor manifestation, No. (%)	
Solitary	1 (8.3)
Multifocal	11 (91.7)
Primary tumor resection	
No	6 (50)
Yes	6 (50)
Estimated relative liver tumor burden, No. (%)	
<10%	2 (16.6)
≥10%~<25%	5 (41.7)
≥25%~<50%	5 (41.7)
Grade, No. (%)	
1	5 (41.7)
2	5 (41.7)
3	1 (8.3)
Unknown	1 (8.3)
Ki-67 Index (%)	10.1 (3.5–16.7)
Hormone-related symptoms, No. (%)	
Absence	5 (41.7)
Presence	7 (58.3)
Primary tumor site, No. (%)	
Lung	1 (8.3)
Pancreas	3 (25)
Gut	6 (50)
Unknown	2 (16.7)
Baseline chromogranin A level (µg/L)	738 (535–1857)
Baseline Serotonin level (nmol/10^9^ platelets)	18 (4–25)
Baseline Tryptophan level (µmol/L)	52.0 (44.5–60.0)
Baseline 5-HIAA level (nmol/L)	320 (99–801)
SIRT sessions	
1	5 (41.7)
2	7 (58.3)

Abbreviations: Y-90 SIRT: Yttrium-90 selective internal radiation therapy, NELM: Neuroendocrine liver metastases, IQR: interquartile range, 5-HIAA: 5-hydroxyindoleacetic acid.

**Table 2:** Treatment-related details for Y-90 SIRT.

Characteristics	Number (%)/Median (IQR)
Treatment, No. (%)	
Sequential lobar	7 (58.3)
Lobar	3 (25)
Whole liver	0 (0)
Selective	2 (16.7)
Pulmonary shunt fraction (%)	6.6 (4.0–10.8)
Administered Y-90 activity (GBq)	1.5 (1.2–1.7)
Dose to the tumor (Gy)	160 (120–365)
Dose to the non-tumor liver (Gy)	40 (30–45)
Treated liver volume (mL)	1400 (625–1500)
Pre-SIRT treatment, No. (%) *	
Octreotide	10 (83.3)
Lanreotide	2 (16.6)
Everolimus	1 (8.3)
Peptide Receptor Radionuclide Therapy	1 (8.3)
Thermal ablation	1 (8.3)
Naïve	1 (8.3)
Post-SIRT treatment, No. (%) *	
Octreotide	9 (75)
Lanreotide	1 (8.3)
Everolimus	2 (16.6)
Peptide Receptor Radionuclide Therapy	1 (8.3)
Capecitabine/Temozolomide	1 (8.3)
None	2 (16.6)
Symptom improvement, No. (%)	
Absence	2 (28.6)
Presence	5 (71.4)

Abbreviations: Y-90 SIRT: Yttrium-90 selective internal radiation therapy, IQR: interquartile range, GBq: gigabecquerel, Gy: gray. * Percentages for pre- and post-SIRT treatments may not sum to 100%, as patients could receive multiple treatments.

**Table 3:** Adverse events for Y-90 SIRT.

Adverse Events *	Patients, *n* (%)
Abdominal Pain	6 (50)
Nausea	3 (25)
Vomiting	3 (25)
Fatigue	2 (16.7)
Fever	1 (8.3)
Abdominal Infection	1 (8.3)
Diarrhea	1 (8.3)
Dyspnea	1 (8.3)
Hyperglycemia	1 (8.3)
Hypertension	1 (8.3)

* Percentages may not sum to 100%, as patients could experience more than one adverse event.

**Table 4:** Baseline characteristics of NELM patients receiving Y-90 SIRT Baseline Characteristics of Included Studies.

Study (Author, Year)	Country	Study Design	Study Period	Sample Size (*n*)	Primary Site (%) GI/Pancreas/Pulmonary/Other/Unknown	Prevalence of Hormone-Related Symptoms (%)	Grade (%) * G1/G2/G3/Unknown	Liver Tumor Burden (%) <25%/25–50%/>50%	Extrahepatic Metastases (%)	Extracted Outcomes
Kennedy et al., 2008 [18]	USA	RM	NR	148	68/19/4/2/7	NR	NR	NR	NR	AE, OS
King et al., 2008 [19]	Australia	PS	2003–2005	34	44.1/23.5/2.9/5.9/23.5	NR	NR	NR	58.8	OS, SI, TR
Rhee et al., 2008 [20]	USA	PM	2001–2006	42	Carcinoid (*n* = 31), Pancreatic islet cell (*n* = 11)	NR	NR	NR	NR	TR
Kalinowski et al., 2009 [21]	Germany	PS	2004–2007	9	55.6/33.3/11.1/0/0	NR	NR	NR	22	OS, TR
Cao et al., 2010 [22]	Australia	RM	2003–2008	58	46.6/24.1/1.7/3.4/24.1	NR	NR	31.1/46.6/10.3	43	OS, TR
Saxena et al., 2010 [23]	Australia	PS	2003–2009	48	46.0/31.0/2.0/4.0/15.0	NR	Well-differentiated: 63%, Moderately- differentiated: 21%, Poorly differentiated: 17%	25.4/37.5/27.1	47.9	OS, TR
Lacin et al., 2011 [24]	Turkey	PS	2008–2009	13	30.8/23.1/0.0/0.0/46.1	NR	NR	NR	38.5	OS, TR
Ezziddin et al., 2012 [25]	Germany	RS	NR	23	Pancreas (60.9), non-pancreatic (39.1)	22	74(G1)/26(G2 + G3)	13/39/48	61	OS, TR, SI
Memon et al., 2012 [26]	USA	RS	2003–2007	40	30.0/22.5/2.5/10.0/35.0	62.5	NR	80/15/5	35	OS, SI
Paprottka et al., 2012 [14]	Germany	RS	NR	42	66.7/21.4/2.4/0.0/9.5	90.5	NR	19/67/14	NR	AE, SI, TR
Shaheen et al., 2012 [27]	Canada	RS	2006–2009	25	28.0/52.0/8.0/0.0/12.0	NR	WHO Stage 2: 56%, Stage 3: 12%, Missing: 32%	<33%: 44%, 33–66%: 20%, >66%: 36%	28	OS, TR
Benson et al., 2013 [28]	USA	PM	2007–2009	43	NR	NR	NR	NR	NR	HPFS, OS, PFS, TR
Ozkan et al., 2013 [29]	Turkey	RS	2006–2011	6	34.0/33.0/0.0/0.0/33.0	100	NR	NR	NR	SI, TR
Sommer et al., 2013 [30]	Germany	RS	2006–2011	45	68.0/0.0/0.0/0.0/31.0	NR	27/44/7/22	<10%: 31.1; 10–50%: 42.2; >50%: 26.7	NR	HPFS
Engelman et al., 2014 [31]	USA	RS	2001–2011	12	NR	NR	Low grade	NR	NR	SI
Peker et al., 2015 [32]	Turkey	RS	2008–2013	30	37.0/23.0/7.0/0.0/33.0	NR	NR	37/27/37	30	OS, TR
Ebeling Barbier et al., 2016 [33]	Sweden	RS	2005–2014	40	83.0/10.0/7.0/0.0/0.0	NR	50/37/10/3	NR	70	OS, TR
Fan et al., 2016 [34]	USA	RS	2004–2012	38	34.0/37.0/5.0/0.0/11.0	NR	Well-differentiated: 45%, Moderately/Poorly differentiated: 16%, Unknown: 39%	<33%: 58%; 33–66%: 16%; >66%: 11%	39	TR
Fidelman et al., 2016 [35]	USA	PS	2010–2013	11	63.6/27.3/9.1/0/0	54.5	NR	36/64/0	18	HPFS, PFS, SI, TR
Filippi et al., 2016 [36]	Italy	RS	NR	15	80.0/13.3/6.7/0.0/0.0	27	G1-2	100/0/0	20	SI, TR
Ludwig et al., 2016 [37]	USA	PS	2006–2012	44	15.9/38.6/6.8/11.4/27.3	NR	NR	NR	50	OS
Singla et al., 2016 [38]	USA	RS	2001–2014	44	54.5/25.0/0.0/20.5/0.0	38.6	60.7/39.3/0/0	79.5/15.9/4.5	NR	OS
Chen et al., 2017 [39]	USA	RM	2004–2015	64	47.0/40.0/5.0/0.0/8.0	NR	50/39/11/0	≤50%: 81%, >50%: 19%	45	AE, HPFS, OS
Do Minh et al., 2017 [40]	Germany	RS	2000–2014	44	70.5/29.5/0.0/0.0/0.0	56.8	84.1/13.6/2.3/0	≤50%: 79.5%, >50%: 20.5%	31.8	HPFS, OS, TR
Jia et al., 2017 [41]	USA	RS	2006–2015	36	41.7/22.2/2.8/5.6/30.6	44.4	66.7(G1 + G2)/33.3(G3)	25/33.3/41.7	44.4	AE, OS, SI, TR
Tomozawa et al., 2018 [42]	USA	RS	2007–2015	93	NR	NR	Well-differentiated: 60.2%, Moderately/Poorly differentiated: 16.1%, Unknown: 23.7%	38.7/33.3/28	34.4	AE, TR
Braat et al., 2019 [43]	Netherlands	RM	2004–2016	244	44.7/31.2/5.3/4.9/13.9	60	20.5/28.3/11.1/40.1	27.5/25.4/46.9	66	AE, SI, TR
Frilling et al., 2019 [44]	UK	RS	2007–2017	24	70.8/25.0/0.0/4.2/0.0	62.5	45.8/41.7/0/12.5	NR	37.5	OS, PFS, TR
Zuckerman et al., 2019 [45]	USA	RS	2009–2015	59	37.3/30.5/11.9/3.4/16.9	37.3	39/37.3/13.6/5.1	NR	35.6	AE, HPFS, OS, PFS, SI, TR
Braat et al., 2020 [46]	Netherlands	RM	NR	44	41.0/40.0/7.0/5.0/7.0	5	30/50/7/13	20.9/20.9/58.2	79	TR
Egger et al., 2020 [47]	USA	RM	2000–2018	51	49.1/31.4/3.9/0.0/15.7	23.5	60.6/24.2/15.2/0	NR	NR	OS, PFS, TR
Tsang et al., 2020 [48]	Canada	RM	2011–2017	49	47.0/31.0/0.0/8.0/14.0	NR	37/25/2/12	<33% (18%), 33–66% (65%), >66% (14%), Unknown (2%)	30.6	AE, OS, TR
Tudela-Lerma et al., 2021 [49]	Spain	RS	2006–2016	30	NR	NR	NR	NR	NR	TR
Ingenper et al., 2022 [50]	Germany	RS	2013–2017	43	51.2/27.9/14.0/2.3/4.7	NR	G1: 19 lesions; G2: 86 lesions; G3: 6 lesions; Unknown: 9 lesions (120 target liver lesions)	NR	NR	TR
Schaarschmidt et al., 2022 [51]	Germany	RM	2007–2019	230	NR	NR	NR	NR	NR	TR
Wong et al., 2022 [52]	USA	RM	2015–2020	170	69.0/24.0/0.0/0.0/7.0	NR	Well-differentiated: 70%, Moderately differentiated: 15%, Poorly differentiated: 15%	NR	48	AE, OS, PFS, TR
Ebbers et al., 2022 [53]	Netherlands	RS	NR	30	47.0/13.0/10.0/10.0/20.0	NR	30/40/13/17	67/33/0	NR	AE, OS, TR
Doyle et al., 2024 [54]	USA	RS	2013–2022	36	47.0/31.0/14.0/2.0/6.0	NR	42/38/3/17	NR	NR	TR
Ingenerf et al., 2024 [55]	Germany	RS	2012–2017	47	49.0/28.0/15.0/2.0/6.0	NR	19/75/4/2	70/21/9	72	OS, TR
Soulen et al., 2024 [56]	USA	RS	2013–2020	37	14.0/43.0/27.0/5.0/11.0	NR	G2	51/27/22	NR	AE, HPFS, OS, PFS, TR,
Briol et al., 2025 [57]	Belgium	RS	2011–2021	50	42.0/46.0/8.0/2.0/2.0	20	10/46/44/0	NR	46	AE, OS, TR, HPFS
Gordon et al., 2025 [58]	USA	RS	2009–2021	18	28/33/28/0/11	NR	39/44/17/0	83/17/0	67	AE, OS, PFS, TR
Our center	Netherlands	RS	2019–2024	12	50/25/8.3/0/16.7	58.3	41.7/41.7/8.3/8.3	58.3/41.7/0	41.7	AE, HPFS, OS, PFS, SI

Abbreviations: AE: Adverse Events, GI: Gastrointestinal, G1/G2/G3: Tumor Grade 1/2/3, HPFS: Hepatic Progression-Free Survival, NELM: Neuroendocrine liver metastases, NR: Not Reported, OS: Overall Survival, PM: Prospective Multicenter, PS: Prospective Single-center, Pulmonary: Pulmonary origin, RM: Retrospective Multicenter, RS: Retrospective Single-center, SI: Symptom Improvement, TR: Treatment Response. * Tumor grade was extracted as originally reported. Most studies used WHO/ENETS G1–G3 classification based on Ki-67 index; in studies reporting only differentiation status (well, moderate, poor) or alternative grading systems (e.g., WHO stage), this was recorded accordingly.

## Data Availability

All data contributing to the analysis of this manuscript is available from the corresponding author upon reasonable request.

## Supplementary Materials

The following supporting information can be downloaded at: https://www.mdpi.com/article/10.3390/diagnostics16010111/s1, Supplementary Text: Assessment of Study Heterogeneity, Publication Bias, and Robustness; Table S1: PRISMA 2020 Checklist; Table S2: Search Strategies; Table S3: SIRT Treatment and Dosimetric Parameters; Table S4: Summary of Risk of Bias Assessment Using ROBINS-I; Table S5: Clinical outcomes across individual studies evaluating Y-90 SIRT for NELM; Table S6: Pooled survival and symptom improvement rates after Y-90 SIRT for NELM; Table S7: Pooled tumor response rates after Y-90 SIRT for NELM; Table S8: Subgroup analysis of pooled tumor response rates by microsphere type (resin vs. glass) based on RECIST criteria; Table S9: Toxicity across individual studies evaluating Y-90 SIRT for NELM; Table S10: Pooled adverse events rates after Y-90 SIRT for NELM; Figure S1: PRISMA flow diagram of study selection; Figures S2–S27: Forest plots, funnel plots, and sensitivity analyses for survival outcomes, tumor response, symptom improvement, and adverse events.

## Abbreviations

The following abbreviations are used in this manuscript:

AE	Adverse Events
ALB	Albumin
ALT	Alanine Aminotransferase
AST	Aspartate Aminotransferase
CapTem	Capecitabine and Temozolomide
CI	Confidence Interval
CR	Complete Response
CT	Computed Tomography
CTCAE	Common Terminology Criteria for Adverse Events
DCR	Disease Control Rate
ECOG PS	Eastern Cooperative Oncology Group Performance Status
GBq	Gigabecquerel
Gy	Gray
HCC	Hepatocellular Carcinoma
HPFS	Hepatic Progression-Free Survival
HR	Hazard Ratio
IQR	Interquartile Range
mRECIST	Modified Response Evaluation Criteria in Solid Tumors
NELM	Neuroendocrine Liver Metastases
NET	Neuroendocrine Tumor
NR	Not Reported/Not Reached
ORR	Objective Response Rate
OS	Overall Survival
PD	Progressive Disease
PFS	Progression-Free Survival
PET/CT	Positron Emission Tomography/CT
PR	Partial Response
PRISMA	Preferred Reporting Items for Systematic Reviews and Meta-Analyses
ROBINS-I	Risk Of Bias In Non-randomized Studies of Interventions
SD	Stable Disease
SI	Symptom Improvement
SIRT	Selective Internal Radiation Therapy
SPECT/CT	Single-Photon Emission Computed Tomography/CT
SSA	Somatostatin Analog
SUVmax	Maximum Standardized Uptake Value
TACE	Transarterial Chemoembolization
TAE	Transarterial Embolization
TARE	Transarterial Radioembolization
WMO	Dutch Medical Research Involving Human Subjects Act
WHO	World Health Organization
Y-90	Yttrium-90

## References

[1] Yao J.C., Hassan M., Phan A., Dagohoy C., Leary C., Mares J.E., Abdalla E.K., Fleming J.B., Vauthey J.N., Rashid A. (2008). One hundred years after “carcinoid”: Epidemiology of and prognostic factors for neuroendocrine tumors in 35,825 cases in the United States. J. Clin. Oncol..

[2] Pavel M., Baudin E., Couvelard A., Krenning E., Oberg K., Steinmuller T., Anlauf M., Wiedenmann B., Salazar R., Barcelona Consensus Conference Participants (2012). ENETS Consensus Guidelines for the management of patients with liver and other distant metastases from neuroendocrine neoplasms of foregut, midgut, hindgut, and unknown primary. Neuroendocrinology.

[3] Dasari A., Shen C., Halperin D., Zhao B., Zhou S., Xu Y., Shih T., Yao J.C. (2017). Trends in the Incidence, Prevalence, and Survival Outcomes in Patients With Neuroendocrine Tumors in the United States. JAMA Oncol..

[4] Pavel M., O’Toole D., Costa F., Capdevila J., Gross D., Kianmanesh R., Krenning E., Knigge U., Salazar R., Pape U.F. (2016). ENETS Consensus Guidelines Update for the Management of Distant Metastatic Disease of Intestinal, Pancreatic, Bronchial Neuroendocrine Neoplasms (NEN) and NEN of Unknown Primary Site. Neuroendocrinology.

[5] Del Rivero J., Perez K., Kennedy E.B., Mittra E.S., Vijayvergia N., Arshad J., Basu S., Chauhan A., Dasari A.N., Bellizzi A.M. (2023). Systemic Therapy for Tumor Control in Metastatic Well-Differentiated Gastroenteropancreatic Neuroendocrine Tumors: ASCO Guideline. J. Clin. Oncol..

[6] Rinke A., Wittenberg M., Schade-Brittinger C., Aminossadati B., Ronicke E., Gress T.M., Muller H.H., Arnold R., PROMID Study Group (2017). Placebo-Controlled, Double-Blind, Prospective, Randomized Study on the Effect of Octreotide LAR in the Control of Tumor Growth in Patients with Metastatic Neuroendocrine Midgut Tumors (PROMID): Results of Long-Term Survival. Neuroendocrinology.

[7] Veldhuis W.B., Walter T., de Vries-Huizing D.M.V., Theysohn J., Barton S., Ekkelenkamp E.D., Lachachi B., de Jong R.J.G., van Golen L.W., Lanzafame H. (2025). PRRT plus holmium-166-SIRT (HEPAR PLuS) versus PRRT-only in patients with metastatic neuroendocrine tumors: A propensity-score matched analysis. J. Neuroendocrinol..

[8] Pavel M., Oberg K., Falconi M., Krenning E.P., Sundin A., Perren A., Berruti A., ESMO Guidelines Committee (2020). Gastroenteropancreatic neuroendocrine neoplasms: ESMO Clinical Practice Guidelines for diagnosis, treatment and follow-up. Ann. Oncol..

[9] Colquhoun S.D. (2018). Neuroendocrine tumors with hepatic metastases: A review of evolving treatment options. Liver Res..

[10] Alrfooh A., Patel A., Laroia S. (2021). Transarterial Radioembolization Agents: A Review of the Radionuclide Agents and the Carriers. Nucl. Med. Mol. Imaging.

[11] Weber M., Lam M., Chiesa C., Konijnenberg M., Cremonesi M., Flamen P., Gnesin S., Bodei L., Kracmerova T., Luster M. (2022). EANM procedure guideline for the treatment of liver cancer and liver metastases with intra-arterial radioactive compounds. Eur. J. Nucl. Med. Mol. Imaging.

[12] Garrou F., Sacchetti G.M., Leva L., Andreatta P., Brambilla M., Morbelli S., Carriero A. (2025). Transarterial radioembolization in neuroendocrine liver metastases 25 years later: A systematic review. Crit. Rev. Oncol. Hematol..

[13] Criss C.R., Makary M.S. (2024). Liver-Directed Locoregional Therapies for Neuroendocrine Liver Metastases: Recent Advances and Management. Curr. Oncol..

[14] Paprottka P.M., Hoffmann R.T., Haug A., Sommer W.H., Raessler F., Trumm C.G., Schmidt G.P., Ashoori N., Reiser M.F., Jakobs T.F. (2012). Radioembolization of symptomatic, unresectable neuroendocrine hepatic metastases using yttrium-90 microspheres. Cardiovasc. Interv. Radiol..

[15] Ramdhani K., Braat A. (2022). The Evolving Role of Radioembolization in the Treatment of Neuroendocrine Liver Metastases. Cancers.

[16] Veenstra E.B., Ruiter S.J.S., de Haas R.J., Bokkers R.P.H., de Jong K.P., Noordzij W. (2022). Post-treatment three-dimensional voxel-based dosimetry after Yttrium-90 resin microsphere radioembolization in HCC. EJNMMI Res..

[17] Page M.J., McKenzie J.E., Bossuyt P.M., Boutron I., Hoffmann T.C., Mulrow C.D., Shamseer L., Tetzlaff J.M., Akl E.A., Brennan S.E. (2021). The PRISMA 2020 statement: An updated guideline for reporting systematic reviews. BMJ Clin. Res. Ed..

[18] Kennedy A.S., Dezarn W.A., McNeillie P., Coldwell D., Nutting C., Carter D., Murthy R., Rose S., Warner R.R., Liu D. (2008). Radioembolization for unresectable neuroendocrine hepatic metastases using resin 90Y-microspheres: Early results in 148 patients. Am. J. Clin. Oncol..

[19] King J., Quinn R., Glenn D.M., Janssen J., Tong D., Liaw W., Morris D.L. (2008). Radioembolization with selective internal radiation microspheres for neuroendocrine liver metastases. Cancer.

[20] Rhee T.K., Lewandowski R.J., Liu D.M., Mulcahy M.F., Takahashi G., Hansen P.D., Benson A.B., Kennedy A.S., Omary R.A., Salem R. (2008). 90Y Radioembolization for metastatic neuroendocrine liver tumors: Preliminary results from a multi-institutional experience. Ann. Surg..

[21] Kalinowski M., Dressler M., Konig A., El-Sheik M., Rinke A., Hoffken H., Gress T.M., Arnold R., Klose K.J., Wagner H.J. (2009). Selective internal radiotherapy with Yttrium-90 microspheres for hepatic metastatic neuroendocrine tumors: A prospective single center study. Digestion.

[22] Cao C.Q., Yan T.D., Bester L., Liauw W., Morris D.L. (2010). Radioembolization with yttrium microspheres for neuroendocrine tumour liver metastases. Br. J. Surg..

[23] Saxena A., Chua T.C., Bester L., Kokandi A., Morris D.L. (2010). Factors predicting response and survival after yttrium-90 radioembolization of unresectable neuroendocrine tumor liver metastases: A critical appraisal of 48 cases. Ann. Surg..

[24] Lacin S., Oz I., Ozkan E., Kucuk O., Bilgic S. (2011). Intra-arterial treatment with 90yttrium microspheres in treatment-refractory and unresectable liver metastases of neuroendocrine tumors and the use of 111in-octreotide scintigraphy in the evaluation of treatment response. Cancer Biother. Radiopharm..

[25] Ezziddin S., Meyer C., Kahancova S., Haslerud T., Willinek W., Wilhelm K., Biersack H.J., Ahmadzadehfar H. (2012). 90Y Radioembolization after radiation exposure from peptide receptor radionuclide therapy. J. Nucl. Med. Off. Publ. Soc. Nucl. Med..

[26] Memon K., Lewandowski R.J., Mulcahy M.F., Riaz A., Ryu R.K., Sato K.T., Gupta R., Nikolaidis P., Miller F.H., Yaghmai V. (2012). Radioembolization for neuroendocrine liver metastases: Safety, imaging, and long-term outcomes. Int. J. Radiat. Oncol. Biol. Phys..

[27] Shaheen M., Hassanain M., Aljiffry M., Cabrera T., Chaudhury P., Simoneau E., Kongkaewpaisarn N., Salman A., Rivera J., Jamal M. (2012). Predictors of response to radio-embolization (TheraSphere(R)) treatment of neuroendocrine liver metastasis. HPB.

[28] Benson A.B., Geschwind J.F., Mulcahy M.F., Rilling W., Siskin G., Wiseman G., Cunningham J., Houghton B., Ross M., Memon K. (2013). Radioembolisation for liver metastases: Results from a prospective 151 patient multi-institutional phase II study. Eur. J. Cancer.

[29] Ozkan F. (2013). Transarterial Chemo and Radioembolization (Yttrium90) of Hepatic Metastasis of Neuroendocrine Tumors: Single Center Experience. Int. J. Hematol. Oncol..

[30] Sommer W.H., Ceelen F., Garcia-Albeniz X., Paprottka P.M., Auernhammer C.J., Armbruster M., Nikolaou K., Haug A.R., Reiser M.F., Theisen D. (2013). Defining predictors for long progression-free survival after radioembolisation of hepatic metastases of neuroendocrine origin. Eur. Radiol..

[31] Engelman E.S., Leon-Ferre R., Naraev B.G., Sharma N., Sun S., O’Dorisio T.M., Howe J., Button A., Zamba G., Halfdanarson T.R. (2014). Comparison of transarterial liver-directed therapies for low-grade metastatic neuroendocrine tumors in a single institution. Pancreas.

[32] Peker A., Cicek O., Soydal C., Kucuk N.O., Bilgic S. (2015). Radioembolization with yttrium-90 resin microspheres for neuroendocrine tumor liver metastases. Diagn. Interv. Radiol..

[33] Barbier C.E., Garske-Roman U., Sandstrom M., Nyman R., Granberg D. (2016). Selective internal radiation therapy in patients with progressive neuroendocrine liver metastases. Eur. J. Nucl. Med. Mol. Imaging.

[34] Fan K.Y., Wild A.T., Halappa V.G., Kumar R., Ellsworth S., Ziegler M., Garg T., Rosati L.M., Su Z., Hacker-Prietz A. (2016). Neuroendocrine tumor liver metastases treated with yttrium-90 radioembolization. Contemp. Clin. Trials.

[35] Fidelman N., Kerlan R.K., Hawkins R.A., Pampaloni M., Taylor A.G., Kohi M.P., Kolli K.P., Atreya C.E., Bergsland E.K., Kelley R.K. (2016). Radioembolization with (90)Y glass microspheres for the treatment of unresectable metastatic liver disease from chemotherapy-refractory gastrointestinal cancers: Final report of a prospective pilot study. J. Gastrointest. Oncol..

[36] Filippi L., Scopinaro F., Pelle G., Cianni R., Salvatori R., Schillaci O., Bagni O. (2016). Molecular response assessed by (68)Ga-DOTANOC and survival after (90)Y microsphere therapy in patients with liver metastases from neuroendocrine tumours. Eur. J. Nucl. Med. Mol. Imaging.

[37] Ludwig J.M., Ambinder E.M., Ghodadra A., Xing M., Prajapati H.J., Kim H.S. (2016). Lung Shunt Fraction prior to Yttrium-90 Radioembolization Predicts Survival in Patients with Neuroendocrine Liver Metastases: Single-Center Prospective Analysis. Cardiovasc. Interv. Radiol..

[38] Singla S., LeVea C.M., Pokuri V.K., Attwood K.M., Wach M.M., Tomaszewski G.M., Kuvshinoff B., Iyer R. (2016). Ki67 score as a potential predictor in the selection of liver-directed therapies for metastatic neuroendocrine tumors: A single institutional experience. J. Gastrointest. Oncol..

[39] Chen J.X., Rose S., White S.B., El-Haddad G., Fidelman N., Yarmohammadi H., Hwang W., Sze D.Y., Kothary N., Stashek K. (2017). Embolotherapy for Neuroendocrine Tumor Liver Metastases: Prognostic Factors for Hepatic Progression-Free Survival and Overall Survival. Cardiovasc. Interv. Radiol..

[40] Do Minh D., Chapiro J., Gorodetski B., Huang Q., Liu C., Smolka S., Savic L.J., Wainstejn D., Lin M., Schlachter T. (2017). Intra-arterial therapy of neuroendocrine tumour liver metastases: Comparing conventional TACE, drug-eluting beads TACE and yttrium-90 radioembolisation as treatment options using a propensity score analysis model. Eur. Radiol..

[41] Jia Z., Paz-Fumagalli R., Frey G., Sella D.M., McKinney J.M., Wang W. (2017). Single-institution experience of radioembolization with yttrium-90 microspheres for unresectable metastatic neuroendocrine liver tumors. J. Gastroenterol. Hepatol..

[42] Tomozawa Y., Jahangiri Y., Pathak P., Kolbeck K.J., Schenning R.C., Kaufman J.A., Farsad K. (2018). Long-Term Toxicity after Transarterial Radioembolization with Yttrium-90 Using Resin Microspheres for Neuroendocrine Tumor Liver Metastases. J. Vasc. Interv. Radiol. JVIR.

[43] Braat A., Kappadath S.C., Ahmadzadehfar H., Stothers C.L., Frilling A., Deroose C.M., Flamen P., Brown D.B., Sze D.Y., Mahvash A. (2019). Radioembolization with (90)Y Resin Microspheres of Neuroendocrine Liver Metastases: International Multicenter Study on Efficacy and Toxicity. Cardiovasc. Interv. Radiol..

[44] Frilling A., Clift A.K., Braat A., Alsafi A., Wasan H.S., Al-Nahhas A., Thomas R., Drymousis P., Habib N., Tait P.N. (2019). Radioembolisation with 90Y microspheres for neuroendocrine liver metastases: An institutional case series, systematic review and meta-analysis. HPB.

[45] Zuckerman D.A., Kennard R.F., Roy A., Parikh P.J., Weiner A.A. (2019). Outcomes and toxicity following Yttrium-90 radioembolization for hepatic metastases from neuroendocrine tumors-a single-institution experience. J. Gastrointest. Oncol..

[46] Braat A., Ahmadzadehfar H., Kappadath S.C., Stothers C.L., Frilling A., Deroose C.M., Flamen P., Brown D.B., Sze D.Y., Mahvash A. (2020). Radioembolization with (90)Y Resin Microspheres of Neuroendocrine Liver Metastases After Initial Peptide Receptor Radionuclide Therapy. Cardiovasc. Interv. Radiol..

[47] Egger M.E., Armstrong E., Martin R.C., Scoggins C.R., Philips P., Shah M., Konda B., Dillhoff M., Pawlik T.M., Cloyd J.M. (2020). Transarterial Chemoembolization vs Radioembolization for Neuroendocrine Liver Metastases: A Multi-Institutional Analysis. J. Am. Coll. Surg..

[48] Tsang E.S., Loree J.M., Davies J.M., Gill S., Liu D., Ho S., Renouf D.J., Lim H.J., Kennecke H.F. (2020). Efficacy and Prognostic Factors for Y-90 Radioembolization (Y-90) in Metastatic Neuroendocrine Tumors with Liver Metastases. Can. J. Gastroenterol. Hepatol..

[49] Tudela-Lerma M., Orcajo-Rincón J., Ramón-Botella E., Álvarez-Luque A., González-Leyte M., Rotger-Regi A., Velasco-Sánchez E., Colón-Rodríguez A. (2021). Efficacy and safety of Yttrium-90 radioembolization in the treatment of neuroendocrine liver metastases. Long-term monitoring and impact on survival. Rev. Esp. De Med. Nucl. E Imagen Mol..

[50] Katharina Ingenerf M., Karim H., Fink N., Ilhan H., Ricke J., Treitl K.M., Schmid-Tannwald C. (2022). Apparent diffusion coefficients (ADC) in response assessment of transarterial radioembolization (TARE) for liver metastases of neuroendocrine tumors (NET): A feasibility study. Acta Radiol..

[51] Schaarschmidt B.M., Wildgruber M., Kloeckner R., Nie J., Steinle V., Braat A., Lohoefer F., Kim H.S., Lahner H., Weber M. (2022). (90)Y Radioembolization in the Treatment of Neuroendocrine Neoplasms: Results of an International Multicenter Retrospective Study. J. Nucl. Med..

[52] Wong T.Y., Zhang K.S., Gandhi R.T., Collins Z.S., O’Hara R., Wang E.A., Vaheesan K., Matsuoka L., Sze D.Y., Kennedy A.S. (2022). Long-term outcomes following 90Y Radioembolization of neuroendocrine liver metastases: Evaluation of the radiation-emitting SIR-spheres in non-resectable liver tumor (RESiN) registry. BMC Cancer.

[53] Ebbers S.C., van Roekel C., Braat M., Barentsz M.W., Lam M., Braat A. (2022). Dose-response relationship after yttrium-90-radioembolization with glass microspheres in patients with neuroendocrine tumor liver metastases. Eur. J. Nucl. Med. Mol. Imaging.

[54] Doyle P.W., Workman C.S., Grice J.V., McGonigle T.W., Huang S., Borgmann A.J., Baker J.C., Taylor J.E., Brown D.B. (2024). Partition Dosimetry and Outcomes of Metastatic Neuroendocrine Tumors after Yttrium-90 Resin Microsphere Radioembolization. J. Vasc. Interv. Radiol..

[55] Ingenerf M., Grawe F., Winkelmann M., Karim H., Ruebenthaler J., Fabritius M.P., Ricke J., Seidensticker R., Auernhammer C.J., Zacherl M.J. (2024). Neuroendocrine liver metastases treated using transarterial radioembolization: Identification of prognostic parameters at 68Ga-DOTATATE PET/CT. Diagn. Interv. Imaging.

[56] Soulen M.C., Teitelbaum U.R., Mick R., Eads J., Mondschein J.I., Dagli M., van Houten D., Damjanov N., Schneider C., Cengel K. (2024). Integrated Capecitabine-Temozolomide with Radioembolization for Liver-Dominant G2 NETs: Long-Term Outcomes of a Single-Institution Retrospective Study. Cardiovasc. Interv. Radiol..

[57] Briol D., Ceratti A., Lhommel R., Annet L., Dragean C., Danse E., Trefois P., Van Den Eynde M., De Cuyper A., Goffette P. (2025). Selective internal radiation therapy for neuroendocrine liver metastases: Efficacy, safety and prognostic factors. A retrospective single institution study. Acta Gastroenterol. Belg.

[58] Gordon A.C., Savoor R., Kircher S.M., Kalyan A., Benson A.B., Hohlastos E., Desai K.R., Sato K., Salem R., Lewandowski R.J. (2025). Yttrium-90 Radiation Segmentectomy for Treatment of Neuroendocrine Liver Metastases. J. Vasc. Interv. Radiol. JVIR.

[59] Yang T.X., Chua T.C., Morris D.L. (2012). Radioembolization and chemoembolization for unresectable neuroendocrine liver metastases—A systematic review. Surg. Oncol..

[60] Kanabar R., Barriuso J., McNamara M.G., Mansoor W., Hubner R.A., Valle J.W., Lamarca A. (2021). Liver Embolisation for Patients with Neuroendocrine Neoplasms: Systematic Review. Neuroendocrinology.

[61] Ngo L., Elnahla A., Attia A.S., Hussein M., Toraih E.A., Kandil E., Killackey M. (2021). Chemoembolization Versus Radioembolization for Neuroendocrine Liver Metastases: A Meta-analysis Comparing Clinical Outcomes. Ann. Surg. Oncol..

[62] Currie B.M., Nadolski G., Mondschein J., Dagli M., Sudheendra D., Stavropoulos S.W., Soulen M.C. (2020). Chronic Hepatotoxicity in Patients with Metastatic Neuroendocrine Tumor: Transarterial Chemoembolization versus Transarterial Radioembolization. J. Vasc. Interv. Radiol. JVIR.

[63] Su Y.K., Mackey R.V., Riaz A., Gates V.L., Benson A.B., Miller F.H., Yaghmai V., Gabr A., Salem R., Lewandowski R.J. (2017). Long-Term Hepatotoxicity of Yttrium-90 Radioembolization as Treatment of Metastatic Neuroendocrine Tumor to the Liver. J. Vasc. Interv. Radiol. JVIR.

[64] Miszczuk M., Chapiro J., Do Minh D., van Breugel J.M.M., Smolka S., Rexha I., Tegel B., Lin M., Savic L.J., Hong K. (2022). Analysis of Tumor Burden as a Biomarker for Patient Survival with Neuroendocrine Tumor Liver Metastases Undergoing Intra-Arterial Therapies: A Single-Center Retrospective Analysis. Cardiovasc. Interv. Radiol..

[65] Chansanti O., Jahangiri Y., Matsui Y., Adachi A., Geeratikun Y., Kaufman J.A., Kolbeck K.J., Stevens J.S., Farsad K. (2017). Tumor Dose Response in Yttrium-90 Resin Microsphere Embolization for Neuroendocrine Liver Metastases: A Tumor-Specific Analysis with Dose Estimation Using SPECT-CT. J. Vasc. Interv. Radiol. JVIR.

[66] Rabei R., Fidelman N. (2023). Liver-Directed Therapy for Neuroendocrine Tumor Metastases in the Era of Peptide Receptor Radionuclide Therapy. Curr. Treat. Options Oncol..

[67] Sahu S., Schernthaner R., Ardon R., Chapiro J., Zhao Y., Sohn J.H., Fleckenstein F., Lin M., Geschwind J.F., Duran R. (2017). Imaging Biomarkers of Tumor Response in Neuroendocrine Liver Metastases Treated with Transarterial Chemoembolization: Can Enhancing Tumor Burden of the Whole Liver Help Predict Patient Survival?. Radiology.

[68] Assouline J., Cannella R., Porrello G., de Mestier L., Dioguardi Burgio M., Raynaud L., Hentic O., Cros J., Tselikas L., Ruszniewski P. (2023). Volumetric Enhancing Tumor Burden at CT to Predict Survival Outcomes in Patients with Neuroendocrine Liver Metastases after Intra-arterial Treatment. Radiol. Imaging Cancer.

[69] Van Der Gucht A., Jreige M., Denys A., Blanc-Durand P., Boubaker A., Pomoni A., Mitsakis P., Silva-Monteiro M., Gnesin S., Lalonde M.N. (2017). Resin Versus Glass Microspheres for (90)Y Transarterial Radioembolization: Comparing Survival in Unresectable Hepatocellular Carcinoma Using Pretreatment Partition Model Dosimetry. J. Nucl. Med. Off. Publ. Soc. Nucl. Med..

[70] Kim H.S., Shaib W.L., Zhang C., Nagaraju G.P., Wu C., Alese O.B., Chen Z., Brutcher E., Renfroe M., El-Rayes B.F. (2018). Phase 1b study of pasireotide, everolimus, and selective internal radioembolization therapy for unresectable neuroendocrine tumors with hepatic metastases. Cancer.

[71] Ekshyyan O., Rong Y., Rong X., Pattani K.M., Abreo F., Caldito G., Chang J.K., Ampil F., Glass J., Nathan C.O. (2009). Comparison of radiosensitizing effects of the mammalian target of rapamycin inhibitor CCI-779 to cisplatin in experimental models of head and neck squamous cell carcinoma. Mol. Cancer Ther..

[72] Exner S., Arrey G., Prasad V., Grötzinger C. (2020). mTOR Inhibitors as Radiosensitizers in Neuroendocrine Neoplasms. Front. Oncol..

[73] Yordanova A., Wicharz M.M., Mayer K., Brossart P., Gonzalez-Carmona M.A., Strassburg C.P., Fimmers R., Essler M., Ahmadzadehfar H. (2018). The Role of Adding Somatostatin Analogues to Peptide Receptor Radionuclide Therapy as a Combination and Maintenance Therapy. Clin. Cancer Res. Off. J. Am. Assoc. Cancer Res..

[74] Grozinsky-Glasberg S., Shimon I., Korbonits M., Grossman A.B. (2008). Somatostatin analogues in the control of neuroendocrine tumours: Efficacy and mechanisms. Endocr. Relat. Cancer.

[75] Cives M., Ghayouri M., Morse B., Brelsford M., Black M., Rizzo A., Meeker A., Strosberg J. (2016). Analysis of potential response predictors to capecitabine/temozolomide in metastatic pancreatic neuroendocrine tumors. Endocr. Relat. Cancer.

