# Supplementary material for: Yttrium-90 Selective Internal Radiation Therapy for Neuroendocrine Liver Metastases: An Institutional Case Series, Updated Systematic Review, and Meta-Analysis
Source: Diagnostics (Basel). 2025 Dec 29;16(1):111. doi: 10.3390/diagnostics16010111 (PMC12786313; doi:10.3390/diagnostics16010111)
Supplement: Supplementary file 1 [file diagnostics-16-00111-s001.zip › diagnostics-4026150-Supplementary Materials.pdf]

Supplementary Text – Assessment of Study Heterogeneity, Publication Bias, and Robustness 3

Supplementary Table S1. PRISMA 2020 Checklist 13

Supplementary Table S2. Search Strategies 16

Supplementary Table S3. SIRT Treatment and Dosimetric Parameters 17

Supplementary Table S4. Summary of Risk of Bias Assessment Using ROBINS-I 22

Supplementary Table S5. Clinical outcomes across individual studies evaluating Y-90 SIRT for NELM 24

Supplementary Table S6. Pooled survival and symptom improvement rates after Y-90 SIRT for NELM 27

Supplementary Table S7. Pooled tumor response rates after Y-90 SIRT for NELM 28

Supplementary Table S8. Subgroup analysis of pooled tumor response rates by microsphere type (resin vs glass) based on RECIST criteria 29

Supplementary Table S9. Toxicity across individual studies evaluating Y-90 SIRT for NELM 30

Supplementary Table S10. Pooled adverse events rates after Y-90 SIRT for NELM 32

Supplementary Figure S1. PRISMA flow diagram of study selection 34

Supplementary Figure S2. Forest plots of 1-, 2-, and 3-year hepatic progression-free survival (HPFS) rates 35

Supplementary Figure S3. Forest plots of 1-, 2-, 3-, and 5-year overall survival (OS) rates 36

Supplementary Figure S4. Forest plots funnel plots, and leave-one-out analyses at 1-, 2-, and 3-year progression-free survival (PFS) rates 37

Supplementary Figure S5. Forest plots (a–c), funnel plots (d–f), and leave-one-out sensitivity analyses (g–i) for objective response rate (ORR) based on RECIST criteria 38

Supplementary Figure S6. Forest plots (a–c), funnel plots (d–f), and leave-one-out sensitivity analyses (g–i) for partial response (PR) rate based on RECIST criteria 39

Supplementary Figure S7. Forest plots (a–c), funnel plots (d–f), and leave-one-out sensitivity analyses (g–i) for stable disease (SD) rate based on RECIST criteria 40

Supplementary Figure S8. Forest plots (a–c), funnel plots (d–f), and leave-one-out sensitivity analyses (g–i) for complete response (CR) rate based on RECIST criteria 41

Supplementary Figure S9. Forest plots (a–c), funnel plots (d–f), and leave-one-out sensitivity analyses (g–i) for progressive disease (PD) rate based on RECIST criteria 42

Supplementary Figure S10. Forest plots (a–c), funnel plots (d–f), and leave-one-out sensitivity analyses (g–i) for disease control rate (DCR) based on RECIST criteria 43

Supplementary Figure S11. Forest plots showing pooled proportions of tumor response outcomes assessed by mRECIST criteria 44

Supplementary Figure S12. Forest plots (a–c), funnel plots (d–f), and leave-one-out sensitivity analyses (g–i) for adverse events related to ALB decrease 45

Supplementary Figure S13. Forest plots (a–c), funnel plots (d–f), and leave-one-out sensitivity analyses (g–i) for adverse events related to ALT elevation 46

Supplementary Figure S14. Forest plots (a–c), funnel plots (d–f), and leave-one-out sensitivity analyses (g–i) for adverse events related to AST elevation 47

Supplementary Figure S15. Forest plots (a–c), funnel plots (d–f), and leave-one-out sensitivity analyses (g–i) for adverse events related to bilirubin elevation 48

Supplementary Figure S16. Forest plots (a–c), funnel plots (d–f), and leave-one-out sensitivity analyses (g–i) for adverse events related to abdominal pain 49

Supplementary Figure S17. Forest plots (a–c), funnel plots (d–f), and leave-one-out sensitivity analyses (g–i) for adverse events related to fatigue 50

Supplementary Figure S18. Forest plots (a–c), funnel plots (d–f), and leave-one-out sensitivity analyses (g–i) for adverse events related to fever 51

Supplementary Figure S19. Forest plots (a–c), funnel plots (d–f), and leave-one-out sensitivity analyses (g–i) for adverse events related to nausea 52

Supplementary Figure S20. Forest plot (a), funnel plot (b), and leave-one-out sensitivity analysis (c) for symptom improvement rate 53

Supplementary Figure S21. Funnel plots for hepatic progression-free survival (HPFS) rates 54

Supplementary Figure S22. Funnel plots for overall survival (OS) rates 55

Supplementary Figure S23. Funnel plots for tumor response outcomes assessed by mRECIST criteria 56

Supplementary Figure S24. Trim-and-fill analyses for tumor response outcomes assessed by mRECIST criteria 57

Supplementary Figure S25. Leave-one-out sensitivity analyses for hepatic progression-free survival (HPFS) 58

Supplementary Figure S26. Leave-one-out sensitivity analyses for overall survival (OS) 59

Supplementary Figure S27. Leave-one-out sensitivity analyses for tumor response outcomes assessed by mRECIST criteria 60

Supplementary Text – Assessment of Study Heterogeneity, Publication Bias, and Robustness

1. Heterogeneity Assessment

Heterogeneity, reflecting between-study variability, was assessed across all pooled outcomes.

1.1 Survival Outcomes

Heterogeneity in survival outcomes varied across endpoints and timepoints, with most pooled estimates demonstrating moderate to substantial between-study variability.

For hepatic progression-free survival (HPFS) (**Supplementary Figure S2**), moderate heterogeneity was observed at 1 year (7 studies; $I^2 = 45.0\%$, $\tau^2 = 0.0049$, $p = 0.0912$), whereas heterogeneity was absent at 2 years (6 studies; $I^2 = 0.0\%$, $\tau^2 = 0$, $p = 0.4829$) and remained low at 3 years (4 studies; $I^2 = 24.4\%$, $\tau^2 = 0.0025$, $p = 0.2652$).

For overall survival (OS) (**Supplementary Figure S3**), moderate heterogeneity was consistently observed across timepoints: 1 year (25 studies; $I^2 = 62.0\%$, $\tau^2 = 0.0096$, $p < 0.0001$), 2 years (24 studies; $I^2 = 58.3\%$, $\tau^2 = 0.0078$, $p = 0.0002$), 3 years (22 studies; $I^2 = 62.3\%$, $\tau^2 = 0.0096$, $p < 0.0001$), and 5 years (9 studies; $I^2 = 63.5\%$, $\tau^2 = 0.0103$, $p = 0.0051$), indicating persistent inter-study variability in OS estimates.

Substantial heterogeneity was detected in progression-free survival (PFS) at all timepoints (**Supplementary Figure S4**): 1 year (6 studies; $I^2 = 82.2\%$, $\tau^2 = 0.0231$, $p < 0.0001$), 2 years (6 studies; $I^2 = 82.4\%$, $\tau^2 = 0.0204$, $p < 0.0001$), and 3 years (5 studies; $I^2 = 73.4\%$, $\tau^2 = 0.0117$, $p = 0.0047$), reflecting high variability in PFS reporting across studies.

1.2 Tumor Response Outcomes

Tumor response outcomes demonstrated varying degrees of heterogeneity across evaluation criteria (RECIST vs. mRECIST) and response categories.

In RECIST-based analyses, substantial heterogeneity was observed for ORR ($I^2 = 85.1\%$, $\tau^2 = 0.7882$; **Supplementary Figure S5a**), PR ($I^2 = 82.2\%$, $\tau^2 = 0.5956$; **Supplementary Figure S6a**), and SD ($I^2 = 86.5\%$, $\tau^2 = 0.7264$; **Supplementary Figure S7a**), while CR and PD showed moderate levels (CR: $I^2 = 55.4\%$, $\tau^2 = 0.7192$; PD: $I^2 = 55.5\%$, $\tau^2 = 0.2561$; **Supplementary Figures S8a, S9a**). DCR had moderate heterogeneity ($I^2 = 54.4\%$, $\tau^2 = 0.2415$; **Supplementary Figure S10a**).

For mRECIST-based outcomes, all results are summarized in **Supplementary Figure S11**. Substantial heterogeneity was noted for CR ($I^2 = 85.6\%$, $\tau^2 = 1.7617$;

Supplementary Figure S11c) and SD (I^2 = 74.4%, τ^2 = 0.3683; **Supplementary Figure S11e**), whereas ORR (I^2 = 74.2%, τ^2 = 0.3826; **Supplementary Figure S11a**) and DCR (I^2 = 51.7%, τ^2 = 0.3256; **Supplementary Figure S11b**) exhibited moderate variability. PR heterogeneity was lower (I^2 = 43.5%, τ^2 = 0.0717; **Supplementary Figure S11d**), and PD demonstrated no heterogeneity (I^2 = 0.0%, τ^2 = 0; **Supplementary Figure S11f**).

Overall, heterogeneity appeared more pronounced for CR and SD under mRECIST, while RECIST-based analyses revealed particularly high variability in PR and SD. The absence of heterogeneity in mRECIST-defined PD suggests greater consistency in this endpoint.

1.3 Adverse Events

Heterogeneity in adverse events varied by toxicity type and grade.

For ALB decrement (**Supplementary Figure S12**), heterogeneity was low for any-grade events (I^2 = 14.3%, τ^2 = 0.0458, p = 0.3206) and absent for both Grade 1–2 (I^2 = 0.0%, τ^2 = 0, p = 0.5145) and Grade ≥3 events (I^2 = 0.0%, τ^2 = 0, p = 0.9324).

For ALT elevation (**Supplementary Figure S13**), substantial heterogeneity was observed for any-grade (I^2 = 83.6%, τ^2 = 1.1229, $p < 0.0001$) and Grade 1–2 events (I^2 = 79.4%, τ^2 = 1.0010, p = 0.0002), while Grade ≥3 events showed low heterogeneity (I^2 = 22.5%, τ^2 = 0.3363, p = 0.2576).

AST elevation also exhibited high heterogeneity at lower grades (**Supplementary Figure S14**)—any-grade (I^2 = 80.3%, τ^2 = 0.7864, p = 0.0001) and Grade 1–2 (I^2 = 79.3%, τ^2 = 0.8314, p = 0.0002)—but none at Grade ≥3 (I^2 = 0.0%, τ^2 = 0, p = 0.9886).

For bilirubin elevation, heterogeneity was minimal across all grades (**Supplementary Figure S15**): any-grade (I^2 = 0.0%, τ^2 = 0, p = 0.6127), Grade 1–2 (I^2 = 28.3%, τ^2 = 0.1561, p = 0.2223), and Grade ≥3 (I^2 = 0.4%, τ^2 = 0.0322, p = 0.4207).

Similarly, for abdominal pain, moderate heterogeneity was found in any-grade events (I^2 = 55.2%, τ^2 = 0.1200, p = 0.0224) and substantial in Grade 1–2 (I^2 = 76.4%, τ^2 = 0.4175, $p < 0.0001$), while Grade ≥3 events showed no heterogeneity (I^2 = 0.0%, τ^2 = 0, p = 0.8523) (**Supplementary Figure S16).**

In fatigue, substantial heterogeneity was detected for both any-grade (I^2 = 90.5%, τ^2 = 1.5624, $p < 0.0001$) and Grade 1–2 (I^2 = 89.0%, τ^2 = 1.9401, $p < 0.0001$), but was considerably lower for Grade ≥3 events (I^2 = 44.2%, τ^2 = 0.0038, p = 0.0735) (**Supplementary Figure S17**).

For fever, moderate heterogeneity was found in any-grade events (I^2 = 40.9%, $\tau^2 < 0.0001$, p = 0.1330), while Grade 1–2 events showed substantial heterogeneity (I^2 =

82.4%, $\tau^2 = 1.2103$, $p < 0.0001$). Grade ≥3 events were homogeneous ($I^2 = 0.0\%$, $\tau^2 = 0$, $p = 0.9854$). (**Supplementary Figure S18)**

Regarding nausea, heterogeneity was moderate for any-grade events ($I^2 = 49.5\%$, $\tau^2 = 0.1041$, $p = 0.0448$), lower for Grade 1–2 ($I^2 = 35.0\%$, $\tau^2 = 0.0728$, $p = 0.1379$), and absent for Grade ≥3 ($I^2 = 0.0\%$, $\tau^2 = 0$, $p = 0.9284$). (**Supplementary Figure S19)**

In general, heterogeneity tended to be low for Grade ≥3 events and more pronounced for lower-grade or more frequently reported adverse events.

1.4 Symptom Improvement

Substantial heterogeneity was found for symptom improvement ($I^2 = 75.0\%$, $\tau^2 = 1.1207$, $p < 0.0001$), potentially due to differences in symptom assessment or patient populations (**Supplementary Figure S20**).

2 Publication Bias Assessment

2.1 Funnel Plot Inspection

2.1.1 Survival Outcomes

HPFS outcomes: Funnel plots for 1-, 2-, and 3-year HPFS rates showed generally symmetric distributions, suggesting a low likelihood of publication bias across timepoints (**Supplementary Figures S21a–c**).

OS outcomes: Funnel plots for 1-, 2-, and 3-year OS rates appeared largely symmetric, indicating minimal risk of publication bias (**Supplementary Figures S22a–c**). The 5-year OS plot showed slight asymmetry, which may be attributable to the limited number of studies available (**Supplementary Figure S22d**).

PFS outcomes: Funnel plots for 1-, 2-, and 3-year PFS rates exhibited moderate asymmetry, especially given the small sample sizes involved. These patterns suggest potential small-study effects or reporting bias (**Supplementary Figure S4d–f**).

Funnel plots were used to assess publication bias across adverse events. Overall, varying degrees of asymmetry were observed, indicating the possibility of small-study effects or selective reporting.

2.1.2 Tumor Response Outcomes

Funnel plots revealed varying degrees of asymmetry across outcomes, suggestive of potential small-study effects or publication bias.

RECIST-based outcomes:

For ORR, mild asymmetry was observed, particularly among smaller studies (**Supplementary Figure S5d**).

The DCR funnel plot showed slight asymmetry, with a tendency toward studies clustering to the right of the mean (**Supplementary Figure S10d**).

CR demonstrated substantial asymmetry, indicating a higher risk of publication bias (**Supplementary Figure S8d**).

For PR, mild to moderate asymmetry was present, consistent with potential small-study effects (**Supplementary Figure S6d**).

In contrast, the SD plot appeared largely symmetric, suggesting minimal publication bias (**Supplementary Figure S7d**).

PD showed moderate asymmetry, though less pronounced than that seen in CR (**Supplementary Figure S9d**).

mRECIST-based outcomes:

The funnel plot for ORR appeared symmetric, with no clear indication of publication bias (**Supplementary Figure S23a**).

DCR showed mild asymmetry, though the overall distribution remained balanced (**Supplementary Figure S23b**).

CR revealed moderate asymmetry, suggestive of potential small-study influence (**Supplementary Figure S23c**).

Funnel plots for PR, SD, and PD were generally symmetric, indicating low likelihood of publication bias (**Supplementary Figures S23d–f**).

2.1.3 Symptom Improvement

The funnel plot for symptom improvement demonstrated mild asymmetry, with a greater number of smaller studies showing positive effects. This may reflect small-study effects or reporting bias favoring benefit (**Supplementary Figure S20b**).

2.1.4 Adverse Events

ALB Decrease: Funnel plots for any-grade and Grade 1–2 ALB decrease showed notable asymmetry, suggesting potential small-study effects. Grade ≥3 also displayed asymmetry, indicating possible publication bias. (**Supplementary Figures S12d–f)**

ALT Elevation: Moderate asymmetry was observed in any-grade and Grade 1–2 ALT events, with a cluster of studies on one side of the mean. Grade ≥3 presented substantial asymmetry, raising concerns for bias. (**Supplementary Figures S13d–f)**

AST Elevation: All grades exhibited asymmetry, especially in the Grade ≥3 group, suggesting a heightened risk of publication bias. (**Supplementary Figures S14d–f**)

Bilirubin Elevation: Mild asymmetry was present across all grades, with more pronounced effects at Grade ≥3 levels. However, the distribution remained relatively balanced. (**Supplementary Figures S15d–f**)

Abdominal Pain: Funnel plots for abdominal pain were relatively symmetric across all severity levels, implying minimal evidence of publication bias. (**Supplementary Figures S16d–f**)

Fatigue: Plots were mostly symmetric for any-grade and Grade 1–2 fatigue. Grade ≥3 showed a minor degree of asymmetry but did not indicate substantial bias. (**Supplementary Figures S17d–f**)

Fever: The funnel plots indicated moderate asymmetry for all grades, particularly for Grade ≥3 events, suggesting a risk of selective reporting. (**Supplementary Figures S18d–f**)

Nausea: Overall, the distribution of studies appeared relatively symmetric. Slight asymmetry was observed in the Grade ≥3 group, indicating a limited possibility of bias. (**Supplementary Figures S19d–f**)

2.2 Egger's Regression Test

Egger's regression test was applied to outcomes with ten or more contributing studies to statistically assess the presence of small-study effects or potential publication bias:

Overall Survival (OS): No significant asymmetry was detected in the 1-year OS rate ($t = 1.27$, $df = 23$, $p = 0.2184$; bias = 1.44, SE = 1.14), 2-year OS rate ($t = 0.37$, $df = 22$, $p = 0.7143$; bias = 0.45, SE = 1.21), or 3-year OS rate ($t = 0.83$, $df = 20$, $p = 0.4186$; bias = 1.03, SE = 1.25).

Overall Response Rate (ORR): No significant asymmetry was detected in the overall dataset ($t = 0.53$, $df = 28$, $p = 0.6028$; bias = 0.61, SE = 1.17), nor was any observed in the resin subgroup ($t = 0.72$, $df = 15$, $p = 0.4810$; bias = 1.36, SE = 1.89).

Disease Control Rate (DCR): Statistically significant asymmetry was identified in the overall dataset ($t = 2.23$, $df = 29$, $p = 0.0335$; bias = 1.19, SE = 0.53), indicating potential publication bias. In contrast, no significant asymmetry was detected in the resin subgroup ($t = 1.42$, $df = 16$, $p = 0.1760$; bias = 1.21, SE = 0.85), suggesting a low likelihood of bias in this subset.

Complete Response (CR): Statistically significant asymmetry was observed in both the overall dataset ($t = –4.01$, $df = 28$, $p = 0.0004$; bias = –1.90, SE = 0.47, $\tau^2 = 1.48$)

and the resin subgroup (t = –3.25, df = 15, p = 0.0054; bias = –2.19, SE = 0.67, τ^2 = 1.58), indicating potential publication bias.

Partial Response (PR): No significant asymmetry was detected in the overall dataset (t = 0.06, df = 28, p = 0.9551; bias = 0.06, SE = 1.10) or in the resin subgroup (t = 0.46, df = 15, p = 0.6499; bias = 0.76, SE = 1.64).

Stable Disease (SD): No significant asymmetry was detected in the overall dataset (t = –1.50, df = 28, p = 0.1446; bias = –1.82, SE = 1.21) or in the resin subgroup (t = –1.79, df = 15, p = 0.0941; bias = –3.49, SE = 1.95).

Progressive Disease (PD): Statistically significant asymmetry was observed in the overall dataset (t = –2.39, df = 29, p = 0.0238; bias = –1.25, SE = 0.52), whereas no significant asymmetry was found in the resin subgroup (t = –1.59, df = 16, p = 0.1311; bias = –1.32, SE = 0.83).

Symptom Improvement: No significant asymmetry was observed (t = 0.59, df = 10, p = 0.5659; bias = 0.61, SE = 1.02).

2.3 Trim-and-Fill Analysis

To assess potential publication bias, the trim-and-fill method was applied to outcomes with evidence of asymmetry in Egger's test. For Disease Control Rate (DCR), nine studies were imputed, slightly reducing the pooled estimate from 0.87 (95% CI: 0.83–0.89) to 0.85 (95% CI: 0.81–0.88). (**Supplementary Figures S24a**)

In the analysis of Complete Response (CR), nine potentially missing studies were added, substantially increasing the estimate from 0.05 (95% CI: 0.03–0.07) to 0.15 (95% CI: 0.12–0.19). (**Supplementary Figures S24b**)

In the CR subgroup using resin microspheres, seven studies were imputed, raising the pooled proportion from 0.06 (95% CI: 0.03–0.10) to 0.11 (95% CI: 0.06–0.18). (**Supplementary Figures S24c**)

For Progressive Disease (PD), thirteen missing studies were estimated, resulting in a decrease in the pooled estimate from 0.13 (95% CI: 0.10–0.19) to 0.09 (95% CI: 0.06–0.13). (**Supplementary Figures S24d**)

These results suggest that publication bias may have led to either underestimation or overestimation of certain outcomes, particularly CR and PD.

3 Sensitivity Analysis: Leave-One-Out Approach

Leave-one-out sensitivity analyses were conducted to assess the robustness of pooled estimates by sequentially omitting individual studies. Overall, the results indicated consistent and reliable findings across all outcomes.

3.1 Survival Outcomes

Hepatic Progression-Free Survival (HPFS):

The pooled estimates for 1-, 2-, and 3-year HPFS remained stable regardless of the study omitted (**Supplementary Figure S25a–c**). The 1-year HPFS estimate was consistently 0.64 (95% CI: 0.58–0.69). The 2-year estimate remained unchanged at 0.41 (95% CI: 0.35–0.47), with no notable heterogeneity. For the 3-year timepoint, the pooled rate ranged from 0.26 to 0.32, with an overall estimate of 0.29 (95% CI: 0.21–0.37). No single study had a disproportionate impact.

Overall Survival (OS):

Pooled OS estimates were robust across 1-, 2-, 3-, and 5-year timepoints (**Supplementary Figure S26a–d**). The 1-year rate remained at 0.82 (95% CI: 0.78–0.87), the 2-year at 0.66 (95% CI: 0.61–0.72), and the 3-year at 0.52 (95% CI: 0.46–0.58). The 5-year OS estimate was 0.34 (95% CI: 0.25–0.44), with only minor variation after omitting individual studies. No significant influence from any single study was observed.

Progression-Free Survival (PFS):

Pooled estimates for PFS were similarly stable across all timepoints (**Supplementary Figure S4g–i**). The 1-year PFS rate was 0.63 (95% CI: 0.43–0.81), the 2-year rate was 0.50 (95% CI: 0.32–0.67), and the 3-year rate was 0.35 (95% CI: 0.19–0.53). While moderate-to-high heterogeneity was present, no individual study substantially altered the results.

3.2 Tumor Response Outcomes

RECIST Criteria

Objective Response Rate (ORR): The leave-one-out analysis for ORR showed consistent results, with the pooled estimate holding at 0.40 (95% CI: 0.32–0.48) across all exclusions (**Supplementary Figure S5g**). This suggests strong robustness despite high heterogeneity (I^2 = 85.1%).

Disease Control Rate (DCR): DCR estimates remained stable at 0.87 (95% CI: 0.83–0.89) regardless of omitted studies (**Supplementary Figure S9g**). None of the individual studies significantly influenced the overall effect.

Complete Response (CR): For CR, the pooled estimate consistently remained at 0.05 (95% CI: 0.03–0.07) across all exclusions (**Supplementary Figure S8g**). Despite moderate heterogeneity, the robustness of the estimate was supported.

Partial Response (PR): The leave-one-out analysis of PR produced a stable pooled estimate of 0.35 (95% CI: 0.28–0.42) (**Supplementary Figure S6g**), with high heterogeneity (I^2 = 82.2%) but no dominant influence from individual studies.

Stable Disease (SD):SD estimates remained robust at 0.48 (95% CI: 0.39–0.56) throughout all iterations (**Supplementary Figure S7g**), with moderate to high heterogeneity (I^2 = 86.5%).

Progressive Disease (PD): The pooled PD rate consistently stayed at 0.13 (95% CI: 0.10–0.16) in all leave-one-out runs (**Supplementary Figure S9g**). No single study had a notable impact on the results, indicating good reliability.

mRECIST Criteria

Objective Response Rate (ORR): Leave-one-out analysis under mRECIST showed a stable pooled ORR of 0.56 (95% CI: 0.40–0.70), with only slight variability across excluded studies (**Supplementary Figure S27a**).

Disease Control Rate (DCR): DCR under mRECIST was highly consistent, with the pooled estimate holding at 0.91 (95% CI: 0.83–0.96) in all scenarios (**Supplementary Figure S27b**).

Complete Response (CR): The CR estimate was 0.08 (95% CI: 0.02–0.25), with moderate variability but no extreme influence from any single study (**Supplementary Figure S27c**).

Partial Response (PR): The pooled PR result remained stable at 0.46 (95% CI: 0.38–0.54), with moderate heterogeneity (**Supplementary Figure S27d**).

Stable Disease (SD): SD held at 0.36 (95% CI: 0.24–0.51) across all study omissions (**Supplementary Figure S27e**), with consistent results.

Progressive Disease (PD): The PD rate remained unchanged at 0.07 (95% CI: 0.05–0.11), with no heterogeneity observed (I^2 = 0%) and identical values across all leave-one-out runs (**Supplementary Figure S27f**).

3.3 Symptom Improvement

Leave-one-out analysis showed that the pooled symptom improvement rate remained stable at 0.77 (95% CI: 0.61–0.88), with estimates ranging narrowly (0.74–0.81) regardless of which study was omitted (**Supplementary Figure S20c**). No single study significantly influenced the overall effect, supporting the robustness of this outcome despite moderate heterogeneity (I^2 = 75.0%).

3.4 Adverse Events

The leave-one-out sensitivity analysis for adverse events indicated high consistency and robustness across all toxicity outcomes.

For ALB decreases adverse events, the pooled incidence was 25% (95% CI: 19%–32%) for any-grade, 23% (95% CI: 17%–30%) for grade 1–2, and 3% (95% CI: 1%–7%) for grade ≥3 events, with low heterogeneity for higher-grade toxicities. (**Supplementary Figure S12g-i**)

ALT elevations demonstrated pooled rates of 21% (95% CI: 9%–40%) for any-grade, 18% (95% CI: 8%–36%) for grade 1–2, and 4% (95% CI: 2%–7%) for grade ≥3 events. These estimates remained stable despite moderate-to-high heterogeneity. (**Supplementary Figure S13g-i**)

For AST elevations, estimates were 29% (95% CI: 16%–48%) for any-grade, 27% (95% CI: 14%–46%) for grade 1–2, and 2% (95% CI: 1%–5%) for grade ≥3 events. The results were consistent across all study omissions. (**Supplementary Figure S14g-i**)

Bilirubin Elevation were low across the board, with pooled proportions of 11% (95% CI: 8%–15%) for any-grade, 10% (95% CI: 6%–14%) for grade 1–2, and 4% (95% CI: 2%–6%) for grade ≥3. All values showed negligible heterogeneity (**Supplementary Figure S15g-i**).

In terms of abdominal pain, the rates were 32% (95% CI: 26%–40%) for any-grade, 36% (95% CI: 26%–48%) for grade 1–2, and 4% (95% CI: 3%–6%) for grade ≥3 events, with robust consistency (**Supplementary Figure S16g-i**).

Fatigue was one of the more common adverse events, with a pooled incidence of 45% (95% CI: 26%–66%) for any-grade and 39% (95% CI: 21%–61%) for grade 1–2. Grade ≥3 fatigue remained rare at 2% (95% CI: 0%–3%) (**Supplementary Figure S17g-i**).

For fever, consistent estimates were observed: 17% (95% CI: 12%–24%) for any-grade, 17% (95% CI: 7%–34%) for grade 1–2, and 2% (95% CI: 1%–5%) for grade ≥3 events. No influential studies were identified. (**Supplementary Figure S18g-i**)

Nausea also showed robust results, with pooled estimates of 27% (95% CI: 23%–31%) for any-grade, 29% (95% CI: 25%–34%) for grade 1–2, and just 1% (95% CI: 0%–2%) for grade ≥3. All results were consistent and unaffected by single-study omission. (**Supplementary Figure S19g-i**)

Collectively, the results of heterogeneity assessment, publication bias analysis, and leave-one-out sensitivity testing support the robustness and internal validity of the meta-analytic findings across efficacy and safety outcomes. While some variability was

observed across endpoints, no single study exerted a disproportionate influence, affirming the reliability of the pooled estimates.

Supplementary Table S1. PRISMA 2020 Checklist

Section and Topic	Item #	Checklist item	Location where item is reported
**TITLE**			
Title	1	Identify the report as a systematic review.	Title page
**ABSTRACT**			
Abstract	2	See the PRISMA 2020 for Abstracts checklist.	Abstract
**INTRODUCTION**			
Rationale	3	Describe the rationale for the review in the context of existing knowledge.	Introduction
Objectives	4	Provide an explicit statement of the objective(s) or question(s) the review addresses.	Introduction
**METHODS**			
Eligibility criteria	5	Specify the inclusion and exclusion criteria for the review and how studies were grouped for the syntheses.	Materials and Methods, 2.2.1.-2.2.2
Information sources	6	Specify all databases, registers, websites, organisations, reference lists and other sources searched or consulted to identify studies. Specify the date when each source was last searched or consulted.	Materials and Methods, 2.2.1.
Search strategy	7	Present the full search strategies for all databases, registers and websites, including any filters and limits used.	Materials and Methods, 2.2.1.
Selection process	8	Specify the methods used to decide whether a study met the inclusion criteria of the review, including how many reviewers screened each record and each report retrieved, whether they worked independently, and if applicable, details of automation tools used in the process.	Materials and Methods, 2.2.2.
Data collection process	9	Specify the methods used to collect data from reports, including how many reviewers collected data from each report, whether they worked independently, any processes for obtaining or confirming data from study investigators, and if applicable, details of automation tools used in the process.	Materials and Methods, 2.2.2.
Data items	10a	List and define all outcomes for which data were sought. Specify whether all results that were compatible with each outcome domain in each study were sought (e.g. for all measures, time points, analyses), and if not, the methods used to decide which results to collect.	Materials and Methods, 2.2.3.
	10b	List and define all other variables for which data were sought (e.g. participant and intervention characteristics, funding sources). Describe any assumptions made about any missing or unclear information.	Materials and Methods, 2.2.2.-2.2.3.
Study risk of bias assessment	11	Specify the methods used to assess risk of bias in the included studies, including details of the tool(s) used, how many reviewers assessed each study and whether they worked independently, and if applicable, details of automation tools used in the process.	Materials and Methods, 2.2.2.
Effect measures	12	Specify for each outcome the effect measure(s) (e.g. risk ratio, mean difference) used in the synthesis or presentation of results.	Materials and Methods, 2.3.
Synthesis methods	13a	Describe the processes used to decide which studies were eligible for each synthesis (e.g. tabulating the study intervention characteristics and comparing against the planned groups for each synthesis (item #5)).	Materials and Methods, 2.3.
	13b	Describe any methods required to prepare the data for presentation or synthesis, such as handling of missing summary statistics, or data conversions.	Materials and Methods, 2.2.1.; Results, 3.2.1
	13c	Describe any methods used to tabulate or visually display results of individual studies and syntheses.	Materials and Methods, 2.3.
	13d	Describe any methods used to synthesize results and provide a rationale for the choice(s). If meta-analysis was performed, describe the model(s), method(s) to identify the presence and extent of statistical heterogeneity, and software package(s) used.	Materials and Methods, 2.3.
	13e	Describe any methods used to explore possible causes of heterogeneity among study results (e.g. subgroup analysis, meta-regression).	Materials and

Section and Topic	Item #	Checklist item	Location where item is reported
			Methods, 2.2.3.
	13f	Describe any sensitivity analyses conducted to assess robustness of the synthesized results.	Materials and Methods, 2.3.
Reporting bias assessment	14	Describe any methods used to assess risk of bias due to missing results in a synthesis (arising from reporting biases).	Materials and Methods, 2.3.
Certainty assessment	15	Describe any methods used to assess certainty (or confidence) in the body of evidence for an outcome.	Not performed
**RESULTS**			
Study selection	16a	Describe the results of the search and selection process, from the number of records identified in the search to the number of studies included in the review, ideally using a flow diagram.	Results, 3.2.1.; Supplementary Figure S1.
	16b	Cite studies that might appear to meet the inclusion criteria, but which were excluded, and explain why they were excluded.	Results, 3.2.1.; Supplementary Figure S1.
Study characteristics	17	Cite each included study and present its characteristics.	Results, Table 4.; Supplementary Table S2.
Risk of bias in studies	18	Present assessments of risk of bias for each included study.	Results, 3.2.2., Figure 3.; Supplementary Table S3.
Results of individual studies	19	For all outcomes, present, for each study: (a) summary statistics for each group (where appropriate) and (b) an effect estimate and its precision (e.g. confidence/credible interval), ideally using structured tables or plots.	Supplementary Materials
Results of syntheses	20a	For each synthesis, briefly summarise the characteristics and risk of bias among contributing studies.	Results, 3.2.3.-3.2.8.; Supplementary Materials
	20b	Present results of all statistical syntheses conducted. If meta-analysis was done, present for each the summary estimate and its precision (e.g. confidence/credible interval) and measures of statistical heterogeneity. If comparing groups, describe the direction of the effect.	Results, 3.2.3.-3.2.8.; Supplementary Materials
	20c	Present results of all investigations of possible causes of heterogeneity among study results.	Results, 3.2.3.-3.2.8.; Supplementary Materials
	20d	Present results of all sensitivity analyses conducted to assess the robustness of the synthesized results.	Results, 3.2.3.-3.2.8.; Supplementary Materials
Reporting biases	21	Present assessments of risk of bias due to missing results (arising from reporting biases) for each synthesis assessed.	Results, 3.2.8.; Supplementary Materials
Certainty of evidence	22	Present assessments of certainty (or confidence) in the body of evidence for each outcome assessed.	Not performed
**DISCUSSION**			

Section and Topic	Item #	Checklist item	Location where item is reported
Discussion	23a	Provide a general interpretation of the results in the context of other evidence.	Discussion
	23b	Discuss any limitations of the evidence included in the review.	Discussion
	23c	Discuss any limitations of the review processes used.	Discussion
	23d	Discuss implications of the results for practice, policy, and future research.	Discussion
**OTHER INFORMATION**			
Registration and protocol	24a	Provide registration information for the review, including register name and registration number, or state that the review was not registered.	Materials and Methods, 2.2.1., PROSPERO CRD420251129021
	24b	Indicate where the review protocol can be accessed, or state that a protocol was not prepared.	Materials and Methods, 2.2.1.
	24c	Describe and explain any amendments to information provided at registration or in the protocol.	Not applicable
Support	25	Describe sources of financial or non-financial support for the review, and the role of the funders or sponsors in the review.	Title page, This research received no external funding.
Competing interests	26	Declare any competing interests of review authors.	Title page, The authors declare no conflicts of interest.
Availability of data, code and other materials	27	Report which of the following are publicly available and where they can be found: template data collection forms; data extracted from included studies; data used for all analyses; analytic code; any other materials used in the review.	Title page

Supplementary Table S2. Search Strategies

	**Search Strategy**
**PubMed**	(Neuroendocrine Tumors[Mesh] OR Neuroendocrine Neoplasms[Mesh]<br>OR neuroendocrine tumor*[tiab] OR neuroendocrine tumour*[tiab]<br>OR neuroendocrine neoplasm*[tiab] OR NET[tiab])<br>AND<br>(Liver Neoplasms[Mesh] OR Liver Metastases[Mesh]<br>OR liver metastas*[tiab] OR hepatic metastas*[tiab]<br>OR neuroendocrine liver metastas*[tiab] OR NELM[tiab])<br>AND<br>(Radioembolization[Mesh] OR Yttrium Radioisotopes[Mesh]<br>OR radioembol*[tiab] OR transarterial radioembolization[tiab]<br>OR selective internal radiation therap*[tiab]<br>OR SIRT[tiab] OR TARE[tiab]<br>OR yttrium-90[tiab] OR Y-90[tiab])
**Embase**	('neuroendocrine tumor'/exp OR 'neuroendocrine neoplasm'/exp<br>OR neuroendocrine tumor*:ti,ab OR neuroendocrine tumour*:ti,ab<br>OR neuroendocrine neoplasm*:ti,ab OR NET:ti,ab)<br>AND<br>('liver metastasis'/exp OR 'liver tumor'/exp<br>OR liver metastas*:ti,ab OR hepatic metastas*:ti,ab<br>OR neuroendocrine liver metastas*:ti,ab OR NELM:ti,ab)<br>AND<br>('radioembolization'/exp OR 'yttrium 90'/exp<br>OR radioembol*:ti,ab OR transarterial radioembolization:ti,ab<br>OR selective internal radiation therap*:ti,ab<br>OR SIRT:ti,ab OR TARE:ti,ab<br>OR yttrium-90:ti,ab OR Y-90:ti,ab)

Supplementary Table S3. SIRT Treatment and Dosimetric Parameters

Study (Author, Year)	Microsphere Type	SIRT Approach	Dose calculation model	Lung Shunt Fraction (%)	Activity Administered (GBq)	Absorbed Dose (Gy)	Non-Tumoral Liver Dose (Gy)	Post-SIRT Therapy
Kennedy et al., 2008	Resin	Lobar: 58.9%, Whole liver: 37.3%, Unknown: 3.8%	BSA	Median 4 (range 0-34.1)	Median 1.14 (range 0.33–3.30)	NR	NR	NR
King et al., 2008	Resin	Whole liver: 64%, Sequential bilobar: 14%, Unilobar: 22%	NR	Mean 6.3	Mean 1.99 ± 0.6 (range 0.92–2.8)	Mean 45.6	NR	NR
Rhee et al., 2008	Resin (47.6%), Glass (52.4%)	NR	Glass: MIRD; Resin: BSA	NR	NR	NR	NR	NR
Kalinowski et al., 2009	NR	Whole liver: 77.8%, Sequential bilobar: 22.2%	BSA	Mean 5.3 ± 2.4 (range 2.6-10)	2.1 ± 0.4 (range 1.45–2.6)	NR	NR	NR
Cao et al., 2010	Resin	Lobar: 94%, Lobe+segment: 6%	BSA	Mean 6.7 ± 5.8	Mean 1.8 ± 0.4	NR	NR	Concurrent chemo in 34 patients (5-FU); Post-SIRT chemo in 5 patients
Saxena et al., 2010	Resin	Whole liver: 40%, Sequential bilobar: 15%, Lobar: 43%, Selective: 2%	BSA	Mean 7.3 ± 6 (range 1–29)	Mean 1.94 ± 0.35 (range 0.92–2.9)	NR	NR	NR
Lacin et al., 2011	Resin	NR	BSA	NR	Mean Right lobe 1.4, Left lobe 1.3	NR	NR	NR
Ezziddin et al., 2012	Resin (91.3%), Glass (8.7%)	NR	BSA	NR	Mean 3.4 ± 2.1	NR	NR	NR
Memon et al., 2012	Glass	Lobar	NR	NR	Median 1.98 (range 0.18–5.02)<br>Mean 2.14 (95%CI 1.9-2.4)	Mean 115 (95%CI 106.6-122.9)<br>Median 113 (range 29-298.7)	NR	NR
Paprottka et al., 2012	Resin	Whole liver: 49%, Lobar: 49%, Segmental: 2%	BSA	Median 5.6 (range 1.9-11.2)	Median 1.57 (range 0.63–2.36),<br>Mean 1.63 ± 0.49	NR	NR	NR

Shaheen et al., 2012	Glass	Sequential lobar: 86%, Whole liver: 4.7%, Lobar: 9.3%	NR	NR	NR	Mean 141.1 (range 90-159)	NR	NR
Benson et al., 2013	Glass	NR	Partition model	NR	NR	Mean 116.2 ± 7.42	NR	NR
Ozkan et al., 2013	Resin	NR	NR	NR	Mean 1.4 (range 1.2–1.7)	NR	NR	NR
Sommer et al., 2013	Resin	NR	BSA	NR	Median 1.7 (range 0.4–4.5)	NR	NR	NR
Engelman et al., 2014	Resin	Lobar or whole liver	NR	NR	NR	NR	NR	Octreotide
Peker et al., 2015	Resin	Right: 40.5%, Left: 19.0%, Whole liver: 57.1%	BSA	NR	Mean 1.65±0.14 (range 1.4–2.0)	NR	NR	NR
Ebeling Barbier et al., 2016	Resin	Sequential lobar: 71%, Single lobar: 29%	BSA	NR	Right: Mean 1.30 (range 0.55-2), Left: 0.90 (range 0.1-1.4), Bilobar: 1.61 (range 1-2.1)	NR	NR	PRRT in 3 patients
Fan et al., 2016	Glass	Segmental: 88%, Whole liver: 10%, Unknown: 2%	BSA	NR	NR	Mean 109 ± 15 (range 57–128)	NR	TACE: 18%, Systemic therapy: 11%
Fidelman et al., 2016	Glass	NR	NR	Median 7.7 (range 3.3–15.5)	Median 2.5 (range 1.1–3.7)	Median 108 (range 77–120)	NR	Octreotide: 8; Everolimus: 4; CAPTEM: 4; Axitinib: 1; Sunitinib: 1; PRRT: 1; Bland TAE: 2; Repeat 90Y RE: 4; TACE: 1; Surgery: 2
Filippi et al., 2016	Resin	52% unilobar, 48% bilobar	BSA	NR	Mean 1.6 ± 0.19	NR	NR	NR
Ludwig et al., 2016	Resin	Whole liver (single: 56.1%, sequential: 13.5%), Right: 25.9%, Left: 3.3%, Selective: 0.8%	BSA	Median 7.5 (IQR 5.6-11)	NR	Median 42.1 (IQR 31.4–63.0)	NR	NR
Singla et al., 2016	NR	NR	NR	NR	NR	NR	NR	Sandostatin: 95.5%

Chen et al., 2017	Resin (67.2%), Glass (32.8%)	Unilobar: 51.6%, Sequential bilobar: 48.4%	NR	NR	NR	NR	NR	NR
Do Minh et al., 2017	Resin	Unilobar (n=8), Bilobar (n=23), Whole liver (n=5)	BSA	NR	Median 1.57 (IQR 1.14–2.50)	NR	NR	NR
Jia et al., 2017	Resin (80.6%), Glass (19.4%)	All unilobar initially (n=6); 1 received whole liver in 2 sessions	NR	Mean 5.3 ± 2.3	Mean 1.8 ± 0.7	NR	NR	NR
Tomozawa et al., 2018	Resin	NR	BSA	NR	Mean 1.92 ± 0.91	NR	NR	9 systemic therapies (everolimus: 7, capecitabine: 1, temozolomide: 1); 1 hepatic arterial infusion
Braat et al., 2019	Resin	NR	BSA (84.4%),50 Gy average liver absorbed dose (13.1%), Partition model (2.5%)	Median 5.6	Median 1.8 (range 0.4–5.5)	NR	NR	Somatostatin analogs: 36.5%, PRRT: 14.3%, Chemotherapy: 38.9%, TKIs/mTOR inhibitors: 35.2%
Frilling et al., 2019	Resin	NR	BSA	Median 6.5 (range 3.1-16.1)	Median 1.4 (range 0.5–2.4)	NR	NR	NR
Zuckerman et al., 2019	Resin (64%), Glass (46%)	NR	NR	Median 3 (range 1-19.6)	Resin: Mean 1.71 (range 0.47–2.25), Glass: Mean 5.43 (range 1.83–16.95)	NR	NR	NR
Braat et al., 2020	Resin	NR	BSA (97%)	Median 3.3 (0.9-33)	Median 1.67 (range 0.4–5.5)	NR	NR	NR
Egger et al., 2020	Glass	NR	NR	NR	NR	NR	NR	NR
Tsang et al., 2020	Resin (69%), Glass (29%), Unknown (2%)	NR	NR	NR	Median 2.2 (range 0.8–3.6)	NR	NR	NR
Tudela-Lerma et al., 2021	NR	Right: 25, Left: 5, Bilobar: 8	NR	NR	Mean 2.4 ± 1.3	NR	NR	NR
Ebbers et al., 2022	Glass	Lobar	Partition model	Median 4.3 (range 0.7-22.1)	Median 2.69 (range 0.703-9.45)	Median 120 (range 30-200)	NR	NR

Ingenper et al., 2022	Resin	Unilobar 38.6%, Bilobar 61.4%	BSA	NR	NR	NR	NR	NR
Schaarschmidt et al., 2022	Resin (46.8%), Glass (53.5%)	Unilobar (11), Sequential Bilobar (4)	Glass: MIRD; Resin: BSA	Mean 5.7 ± 5.6	Glass: Mean 3.78 ± 2.24, Resin: Mean 1.26 ± 0.59	NR	NR	NR
Wong et al., 2022	Resin	Unilobar 76.9%, bilobar 23.1%	BSA (91%)	NR	Unilobar: Median1.3 (IQR: 0.9-1.5), Bilobar: Median1.9 (IQR: 1.7-2.2)	NR	NR	NR
Doyle et al., 2024	Resin	Whole liver (n=8), Sequential lobar (n=10), Unilobar (n=5)	Partition model	NR	Median 1.0 (IQR: 0.80-1.30)	Median 101 (IQR 71-150)	NR	NR
Ingenerf et al., 2024	Resin	Whole liver: 44.9%, Sequential whole-liver: 19.6%, Unilobar: 19.6%, selected: 0.4%	BSA	Mean 4.8 ± 1.9 (range 2-11.7)	Median 0.796 (range 0.208-1.573)	NR	NR	PRRT: 57%, Biotherapy: 59%, Chemotherapy: 25%, Everolimus: 19%, Liver-directed: 21%
Soulen et al., 2024	Resin	NR	BSA	Median 3 (range 0.5-14), Mean 4.5	Mean 1.77 (range 0.78-2.74)	NR	NR	CapTem (capecitabine + temozolomide) :100%, PRRT:19%, Chemotherapy:16%, Embolization:16%, Resection/ablation:14%, 2nd CapTemY90:11%, Targeted agent:11%, Radiation:11%
Briol et al., 2025	Resin (80%), Glass (20%)	NR	BSA	NR	Median 1.70 (range: 1.57-1.83)	NR	NR	NR
Gordon et al., 2025	Glass	Segmental	MIRD	NR	NR	Median 235.3 (95%CI 265.0–504.5)	NR	Chemotherapy: 22%, PRRT: 33%
Our center	Resin	Sequential lobar: 58.3%, lobar: 25%, Selective 16.6%	Partition model	Median 6.6 (IQR 4-10.8)	Median 1.5 (IQR 1.2-1.7)	Median 160 (IQR 120-365)	Median 40 (IQR 30-45)	CapTem (capecitabine + temozolomide):8.3%, PRRT:8.3%, Chemotherapy:16%, Everolimus:16.6%, Somatostatin analogs:83.3%

Abbreviations:

BSA: Body Surface Area (dosimetry model), CI: Confidence Interval, GBq: Gigabecquerel, Gy: Gray (unit of absorbed radiation dose), NR: Not Reported, SIRT: Selective internal radiation therapy.

Supplementary Table S4. Summary of Risk of Bias Assessment Using ROBINS-I

Study	Confounding	Selection of participants	Classification of interventions	Deviations from intended interventions	Missing data	Measurement of outcomes	Selection of the reported result	Overall
Kennedy et al., 2008	Serious	Moderate	Low	Low	Moderate	Moderate	Moderate	Serious
King et al., 2008	Serious	Low	Low	Low	Low	Low	Low	Serious
Rhee et al., 2008	Moderate	Moderate	Low	Low	Low	Low	Low	Moderate
Kalinowski et al., 2009	Moderate	Moderate	Low	Low	Low	Moderate	Moderate	Moderate
Cao et al., 2010	Moderate	Moderate	Low	Low	Low	Low	Moderate	Moderate
Saxena et al., 2010	Moderate	Moderate	Low	Low	Low	Low	Low	Moderate
Lacin et al., 2011	Serious	Moderate	Low	Low	Low	Moderate	Moderate	Serious
Ezziddin et al., 2012	Moderate	Low	Low	Low	Low	Low	Low to Moderate	Moderate
Memon et al., 2012	Serious	Moderate	Low	Low	Low	Low	Low	Serious
Paprottka et al., 2012	Serious	Moderate	Low	Low	Low	Moderate	Moderate	Serious
Shaheen et al., 2012	Serious	Moderate	Low	Low	Low	Moderate	Moderate	Serious
Benson et al., 2013	Moderate	Low	Low	Low	Low	Low	Low	Low
Ozkan et al., 2013	Serious	Moderate	Low	Low	Low	Moderate	Moderate	Serious
Sommer et al., 2013	Moderate	Low	Low	Low	Moderate	Low	Low	Moderate
Engelman et al., 2014	Serious	Moderate	Low	Low	Low	Moderate	Moderate	Serious
Peker et al., 2015	Serious	Moderate	Low	Low	Low	Moderate	Moderate	Serious
Fan et al., 2016	Moderate	Low	Low	Low	Low	Low	Low to Moderate	Moderate
Fidelman et al., 2016	Moderate	Moderate	Low	Low	Low	Low	Low	Moderate
Filippi et al., 2016	Serious	Moderate	Low	Low	Low	Moderate	Low	Serious
Ebeling Barbier et al., 2016	Serious	Moderate	Low	Low	Low	Moderate	Low	Serious
Ludwig et al., 2016	Moderate	Low	Low	Low	Low	Low	Moderate	Moderate
Singla et al., 2016	Serious	Moderate	Moderate	Low	Moderate	Low	Moderate	Serious

Chen et al., 2017	Moderate	Moderate	Low	Moderate	Moderate	Low	Low	Moderate
Do Minh et al., 2017	Moderate	Low	Low	Low	Low	Low	Low	Moderate
Jia et al., 2017	Serious	Moderate	Low	Low	Moderate	Low	Moderate	Serious
Tomozawa et al., 2018	Moderate	Moderate	Low	Low	Moderate	Low	Low	Moderate
Braat et al., 2019	Moderate	Low	Low	Low	Moderate	Low	Moderate	Moderate
Frilling et al., 2019	Moderate	Moderate	Low	Low	Low	Low	Low	Moderate
Zuckerman et al., 2019	Moderate	Low	Low	Low	Moderate	Low	Moderate	Moderate
Braat et al., 2020	Serious	Moderate	Low	Low	Moderate	Low	Moderate	Serious
Egger et al., 2020	Moderate	Moderate	Low	Low	Moderate	Low	Low	Moderate
Tsang et al., 2020	Moderate	Low	Low	Low	Low	Low	Low	Moderate
Tudela-Lerma et al., 2021	Serious	Moderate	Low	Low	Moderate	Low	Moderate	Serious
Ebbers et al., 2022	Moderate	Low	Low	Low	Low	Low	Low	Moderate
Ingenper et al., 2022	Moderate	Moderate	Low	Low	Low	Low	Low	Moderate
Schaarschmidt et al., 2022	Moderate	Moderate	Low	Low	Low	Low	Low	Moderate
Wong et al., 2022	Moderate	Moderate	Low	Low	Low	Low	Low	Moderate
Doyle et al., 2024	Moderate	Moderate	Low	Low	Low	Low	Low	Moderate
Ingenerf et al., 2024	Moderate	Moderate	Low	Low	Low	Low	Low	Moderate
Soulen et al., 2024	Moderate	Moderate	Low	Low	Low	Low	Low	Moderate
Briol et al., 2025	Moderate	Low	Low	Low	Moderate	Low	Moderate	Moderate
Gordon et al., 2025	Moderate	Low	Low	Low	Low	Low	Moderate	Moderate

Abbreviations:

BSA: Body Surface Area (dosimetry model), CI: Confidence Interval, GBq: Gigabecquerel, Gy: Gray (unit of absorbed radiation dose), NR: Not Reported, SIRT: Selective internal radiation therapy.

Supplementary Table S5. Clinical outcomes across individual studies evaluating Y-90 SIRT for NELM

Study (Author, Year)	Response Criteria	Sample Size (n)*	ORR	DCR	CR	PR	SD	PD	Symptom Improvement (%)	Median HPFS (months)	Median OS (months)	Median PFS (months)
Kennedy et al., 2008	WHO & RECIST	148	NR	NR	NR	NR	NR	NR	NR	NR	70	NR
King et al., 2008	RECIST	33	17	22	6	11	5	11	48	NR	mean 27.6 ± 2.3	NR
Rhee et al., 2008	RECIST	29	15	27	0	15	12	2	NR	NR	NR	NR
Kalinowski et al., 2009	RECIST	9	6	9	0	6	3	0	NR	NR	NR	NR
Cao et al., 2010	RECIST	51	20	34	6	14	14	17	NR	NR	36 (range 1–61)	NR
Saxena et al., 2010	RECIST	48	26	37	7	19	11	11	NR	NR	35 (range 5–63)	NR
Lacin et al., 2011	RECIST	10	5	9	1	4	4	1	NR	NR	20	7.25
Ezziddin et al., 2012	RECIST	23	7	21	0	7	14	2	80	NR	29 (95% CI 4–54)	NR
Memon et al., 2012	WHO & EASL	40	NR	NR	NR	NR	NR	NR	84	NR	34.4 (range 1.1–75.5)	NR
Paprottka et al., 2012	RECIST	40	9	39	0	9	30	1	94.7	NR	NR	NR
Shaheen et al., 2012	mRECIST	25	NR	NR	NR	15	NR	NR	NR	NR	35.3	NR
Benson et al., 2013	RECIST	43	9	40	0	9	31	3	NR	17.9 (95CI% 13.6–NC)	NR	NR
Ozkan et al., 2013	RECIST	6	NR	4	NR	NR	NR	2	75	7.5	14.5	7.5
Sommer et al., 2013	RECIST	45	NR	NR	NR	NR	NR	NR	NR	NR	NR	24.2 (95% CI 12.42-31.69)
Engelman et al., 2014	NR	12	NR	NR	NR	NR	NR	NR	83.3	NR	26.8	NR
Peker et al., 2015	RECIST	29	13	24	1	12	11	5	NR	NR	39.0 (95%CI 12.6–65.4)	NR

Ebeling Barbier et al., 2016	mRECIST	54	29	51	0	29	22	3	NR	NR	24.7 (range 3-117)	NR
Fan et al., 2016	RECIST	35	9	30	3	6	21	5	NR	NR	29.2 (95% CI 18.0–40.4)	NR
Fidelman et al., 2016	RECIST	11	8	11	0	8	3	0	100	19 (range 4.0-48.0)	NR	8.9 (range 1.0–48.0)
Filippi et al., 2016	mRECIST	15	7	15	0	7	8	0	100	NR	33 (95%CI 26.9–35)	27 (95%CI 21–32)
Ludwig et al., 2016	NR	44	NR	NR	NR	NR	NR	NR	NR	NR	27.4 (95% CI 12.73–55.23)	NR
Singla et al., 2016	NR	44	NR	NR	NR	NR	NR	NR	NR	NR	66.8 (range 54.4–113.1)	NR
Chen et al., 2017	RECIST	64	NR	NR	NR	NR	NR	NR	NR	15.7	48.2	NR
Do Minh et al., 2017	RECIST	36	0	32	0	0	32	4	NR	11.2	23.6	NR
Jia et al., 2017	RECIST	36	19	32	0	19	13	4	93.8	NR	41 (25.5-56.5)	NR
Tomozawa et al., 2018	RECIST	52	13	48	0	13	35	4	NR	NR	28.0 (95% CI 16.2–39.8)	NR
Braat et al., 2019	RECIST/mRECIST	244/126	38/54	223/115	4/10	34/44	185/61	21/11	79	NR	31.2 (95% CI 26.4–36)	NR
Frilling et al., 2019	RECIST	24	14	21	1	13	7	1	NR	NR	57 (95% CI 41–NC)	41 (95% CI 31–NC)
Zuckerman et al., 2019	mRECIST	52	30	48	3	27	18	4	22.7	18 (95% CI 13–27)	31 (95% CI 27–NC)	13 (95% CI: 8–19)
Braat et al., 2020	RECIST	44	7	40	1	6	33	4	NR	NR	42 (95% CI 18–61)	NR
Egger et al., 2020	RECIST	46	11	38	2	9	27	8	NR	NR	35.9	19.9
Tsang et al., 2020	RECIST	49	26	42	0	26	16	6	NR	NR	27.2 (95% CI 8-46.5)	NR
Tudela-Lerma et al., 2021	RECIST	30	23	30	0	23	7	0	NR	NR	NR	NR
Ebbers et al., 2022	RECIST	31	4	21	0	4	17	10	NR	NR	19.3 (95% CI 12–34.3)	NR

Ingenper et al., 2022	RECIST	43	13	37	0	13	24	6	NR	NR	NR	NR
Schaarschmidt et al., 2022	RECIST	218	95	192	5	90	97	26	NR	NR	mean 38.9 ± 33.0	NR
Wong et al., 2022	RECIST	99	44	83	5	39	39	16	NR	NR	33 (95% CI 25–NR)	25 (95% CI 22–35)
Doyle et al., 2024	RECIST/mRECIST	36/33	27/28	35/32	7/13	20/15	8/4	1/1	NR	NR	NR	NR
Ingenerf et al., 2024	RECIST	47	6	41	0	6	35	6	NR	28.3 (95% CI 18.2-38.5)	49.6 (95% CI 18.2-81)	13.1 (95% CI 10.3-15.9)
Soulen et al., 2024	RECIST	32	23	32	4	19	9	0	NR	35 (95% CI 21–45)	41 (95% CI 24–87)	36 (95% CI 20–39)
Briol et al., 2025	RECIST	36	8	33	0	8	25	3	NR	14	NR	3.91 (95% CI 1.3–11.4)
Gordon et al., 2025	RECIST	18	15	18	4	11	3	0	NR	NR	69.4 (95CI% 23.1-99.4)	12.2 (95% CI 4.6–28.8)
Our center	NR	NR	NR	NR	NR	NR	NR	NR	71.4	15.3 (95% CI 6.2–50.1)	33.3 (95 CI% NC-NC)	11.2 (95% CI 5.7-19.8)

Abbreviations:

CI: Confidence Interval, CR: Complete Response, DC: Disease Control, EASL: European Association for the Study of the Liver, HPFS: Hepatic Progression-Free Survival, mRECIST: Modified Response Evaluation Criteria in Solid Tumors, NC: Not Reach, NELM: Neuroendocrine liver metastases, NR: Not Reported, OR: Objective Response, OS: Overall Survival, PD: Progressive Disease, PFS: Progression-Free Survival, PR: Partial Response, RECIST: Response Evaluation Criteria in Solid Tumors, SD: Stable Disease, WHO: World Health Organization.

* In some studies, the number refers to patients assessed for tumor response, which may be fewer than the total enrolled.

Supplementary Table S6. Pooled survival and symptom improvement rates after Y-90 SIRT for NELM

Outcome Type	Timepoint	No. of Studies	Pooled Rate	95% CI
HPFS	1-year	7	0.64	0.58–0.69
	2-year	6	0.41	0.35–0.47
	3-year	4	0.29	0.21–0.37
OS	1-year	25	0.82	0.78–0.87
	2-year	24	0.66	0.61–0.72
	3-year	22	0.52	0.46–0.58
	5-year	9	0.34	0.25–0.44
PFS	1-year	6	0.63	0.43–0.81
	2-year	6	0.50	0.32–0.67
	3-year	5	0.35	0.19–0.53
Symptom Improvement	—	12	0.77	0.61–0.88

Abbreviations:

CI: Confidence Interval, HPFS: Hepatic Progression-Free Survival, NELM: Neuroendocrine liver metastases, OS: Overall Survival, PFS: Progression-Free Survival.

Supplementary Table S7. Pooled tumor response rates after Y-90 SIRT for NELM

Response Type	Evaluation Criteria	No. of Studies	Pooled Rate	95% CI
ORR	RECIST	30	0.40	0.32–0.48
DCR		31	0.87	0.83–0.89
CR		30	0.05	0.03–0.07
PR		30	0.35	0.28–0.42
SD		30	0.48	0.39–0.56
PD		31	0.13	0.10–0.16
ORR	mRECIST	5	0.56	0.40–0.70
DCR		5	0.91	0.83–0.96
CR		5	0.08	0.02–0.25
PR		5	0.46	0.38–0.54
SD		5	0.36	0.24–0.51
PD		5	0.07	0.05–0.11

Abbreviations:

CI: Confidence Interval, CR: Complete Response, DCR: Disease Control Rate, mRECIST: Modified Response Evaluation Criteria in Solid Tumors, NELM: Neuroendocrine liver metastases, ORR: Objective Response Rate, PD: Progressive Disease, PR: Partial Response, RECIST: Response Evaluation Criteria in Solid Tumors, SD: Stable Disease, SIRT: Selective internal Radiation Therapy.

Supplementary Table S8. Subgroup analysis of pooled tumor response rates by microsphere type (resin vs glass) based on RECIST criteria

Response Type	Evaluation Criteria	Microsphere Type	No. of Studies	Pooled Rate	95% CI
ORR	RECIST	Resin	17	0.38	0.27–0.49
	RECIST	Glass	7	0.39	0.20–0.62
DCR	RECIST	Resin	18	0.86	0.81–0.90
	RECIST	Glass	7	0.83	0.77–0.88
CR	RECIST	Resin	17	0.06	0.03–0.10
	RECIST	Glass	7	0.08	0.04–0.14
PR	RECIST	Resin	17	0.32	0.24–0.40
	RECIST	Glass	7	0.33	0.18–0.52
SD	RECIST	Resin	17	0.49	0.36–0.61
	RECIST	Glass	7	0.50	0.35–0.64
PD	RECIST	Resin	18	0.13	0.10–0.19
	RECIST	Glass	7	0.17	0.12–0.23

Abbreviations:

CI: Confidence Interval, CR: Complete Response, DCR: Disease Control Rate, ORR: Objective Response Rate, PD: Progressive Disease, PR: Partial Response, RECIST: Response Evaluation Criteria in Solid Tumors, SD: Stable Disease.

Supplementary Table S9. Toxicity across individual studies evaluating Y-90 SIRT for NELM

Study (Author, Year)	Sample Size (n)	Any grade								Grade 1-2								Grade ≥3							
		ALB	ALT	AST	Bilirubin	Abdominal Pain	Fatigue	Fever	Nausea	ALB	ALT	AST	Bilirubin	Abdominal Pain	Fatigue	Fever	Nausea	ALB	ALT	AST	Bilirubin	Abdominal Pain	Fatigue	Fever	Nausea
Kennedy et al., 2008	185	/	/	/	/	/	/	/	/	/	/	/	/	/	/	/	/	/	/	/	/	5	12	/	6
Paprottka et al., 2012	42	/	/	/	/	/	/	/	/	/	/	/	/	29	16	24	14	/	/	/	/	/	/	/	/
Chen et al., 2017	67	/	27	28	10	26	36	13	22	/	22	27	10	24	35	12	21	/	5	1	0	/	0	0	1
Jia et al., 2017	36	/	/	/	/	10	31	8	16	/	/	/	/	10	31	8	15	/	/	/	/	/	1	1	1
Tomozawa et al., 2018	52	12	5	5	2	/	/	/	/	11	5	4	2	/	/	/	/	1	0	1	0	/	/	/	/
Braat et al., 2019	244	/	/	/	/	66	69	/	56	/	/	/	/	/	/	/	/	/	/	/	/	/	/	/	/
Zuckerman et al., 2019	59	19	25	28	6	20	7	/	14	17	22	26	3	16	7	/	13	2	3	2	3	/	0	/	1
Egger et al., 2020	/	/	/	/	/	/	/	/	/	/	/	/	/	19	/	/	/	/	/	/	/	/	/	/	/
Tsang et al., 2020	49	/	3	13	5	/	/	/	/	/	2	12	5	/	2	/	/	/	1	1	0	/	/	/	/

Ebbers et al., 2022	35	6	13	17	4	19	23	7	13	6	12	16	1	/	23	7	13	0	1	1	3	/	0	0	0
Wong et al., 2022	170	/	/	/	/	/	/	/	/	/	/	/	/	/	/	/	/	/	1	/	5	/	/	/	1
Soulen et al., 2024	37	/	/	/	/	7	18	/	10	/	/	/	/	7	17	/	10	/	/	/	/	0	1	/	0
Briol et al., 2025	50	/	/	/	/	12	11	1	6	/	/	/	/	12	11	1	6	/	/	/	/	0	0	0	0
Gordon et al., 2025	18	3	1	0	2	5	15	1	5	3	1	0	2	5	14	1	5	0	0	0	0	0	1	0	0
Our center	12	/	/	/	/	6	2	1	3	/	/	/	/	6	2	1	3	/	/	/	/	0	0	0	0

Abbreviations:

ALB: Albumin, ALT: Alanine Aminotransferase, AST: Aspartate Aminotransferase, NELM: Neuroendocrine liver metastases.

"/" = Not reported.

Supplementary Table S10. Pooled adverse events rates after Y-90 SIRT for NELM

Outcome Type	Grade	No. of Studies	Pooled Rate	95% CI
	Any-grade	4	0.25	0.19–0.32
ALB Decrement	Grade 1-2	4	0.23	0.17–0.30
	Grade ≥3	4	0.03	0.01–0.07
	Any-grade	6	0.21	0.09–0.40
ALT Elevation	Grade 1-2	6	0.18	0.08–0.36
	Grade ≥3	7	0.04	0.02–0.07
	Any-grade	6	0.29	0.16–0.48
AST Elevation	Grade 1-2	6	0.27	0.14–0.46
	Grade ≥3	6	0.02	0.01–0.05
	Any-grade	6	0.11	0.08–0.15
Bilirubin Elevation	Grade 1-2	6	0.1	0.06–0.14
	Grade ≥3	7	0.04	0.02–0.06
	Any-grade	9	0.32	0.26–0.40
Abdominal Pain	Grade 1-2	9	0.36	0.26–0.48
	Grade ≥3	11	0.04	0.03–0.06
	Any-grade	9	0.45	0.26–0.66
Fatigue	Grade 1-2	10	0.39	0.21–0.61
	Grade ≥3	9	0.02	0.00–0.03
	Any-grade	6	0.17	0.12–0.24
Fever	Grade 1-2	7	0.17	0.07–0.34
	Grade ≥3	6	0.02	0.01–0.05
	Any-grade	9	0.27	0.23–0.31
Nausea	Grade 1-2	9	0.29	0.25–0.34
	Grade ≥3	10	0.02	0.01–0.04

Abbreviations:

ALB: Albumin, ALT: Alanine Aminotransferase, AST: Aspartate Aminotransferase, NELM: Neuroendocrine liver metastases.

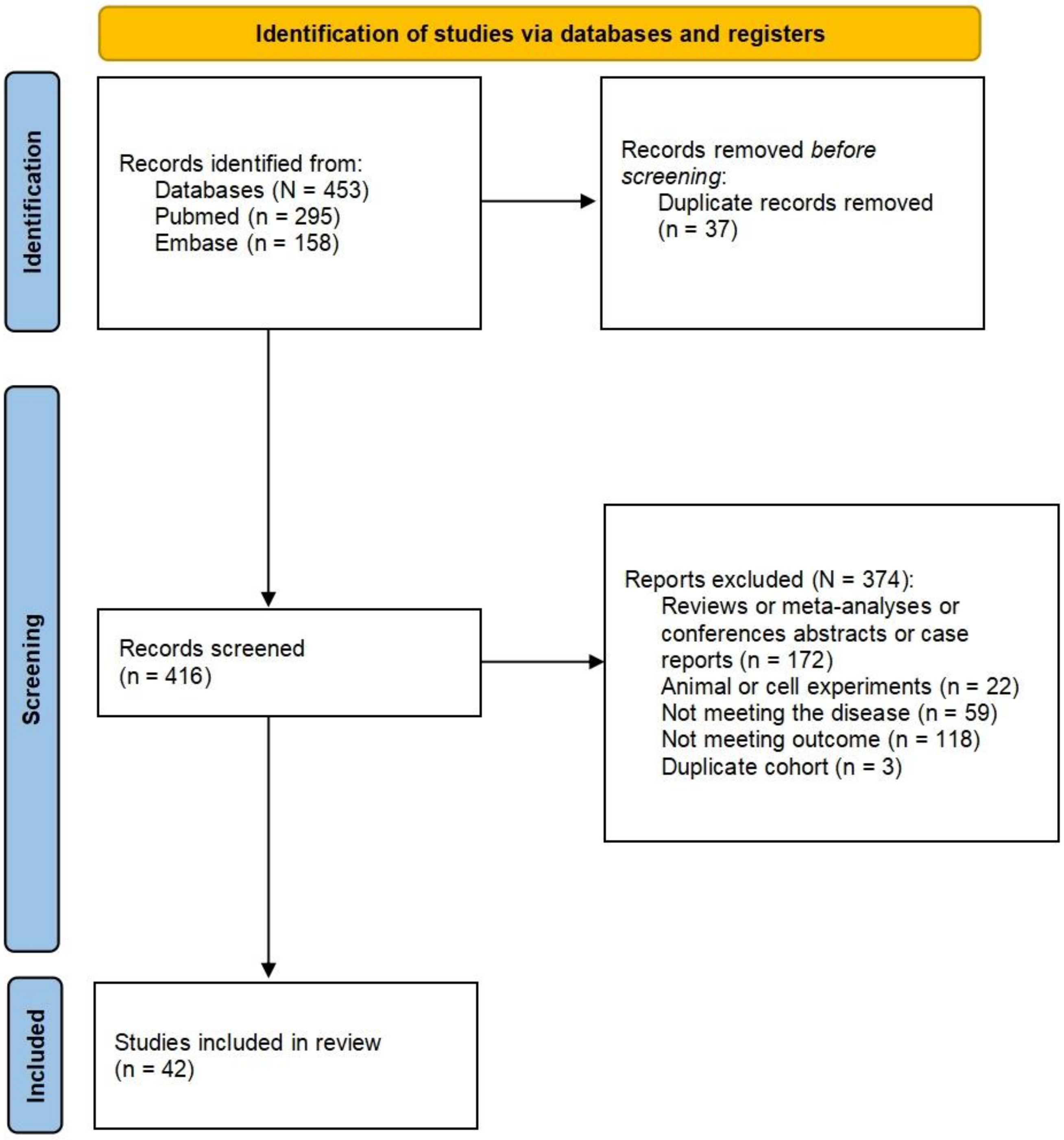

Supplementary Figure S1. PRISMA flow diagram of study selection

From: Page MJ, McKenzie JE, Bossuyt PM, Boutron I, Hoffmann TC, Mulrow CD, et al. The PRISMA 2020 statement: an updated guideline for reporting systematic reviews. BMJ 2021;372:n71. doi: 10.1136/bmj.n71

For more information, visit: http://www.prisma-statement.org/

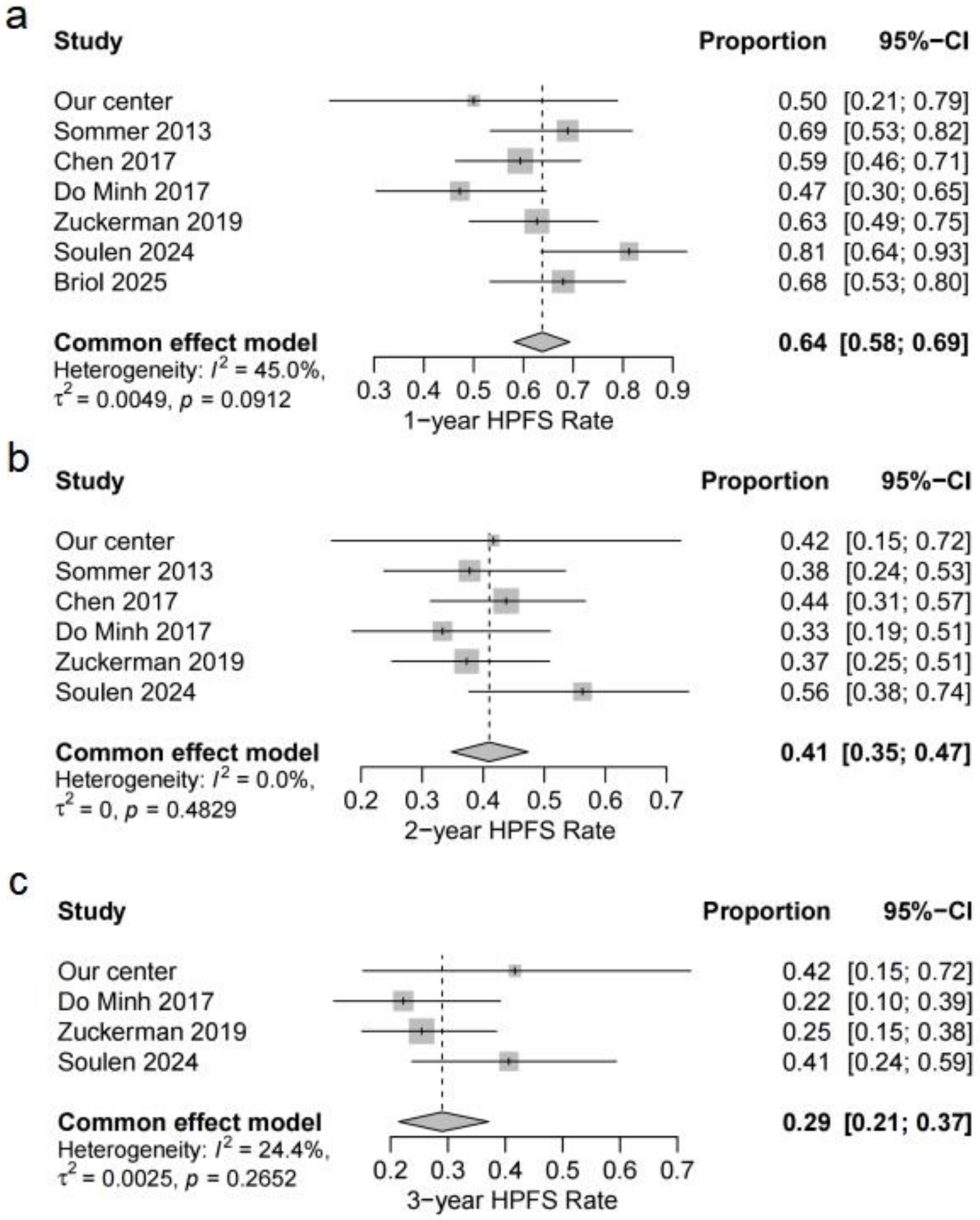

Supplementary Figure S2. Forest plots of 1-, 2-, and 3-year hepatic progression-free survival (HPFS) rates

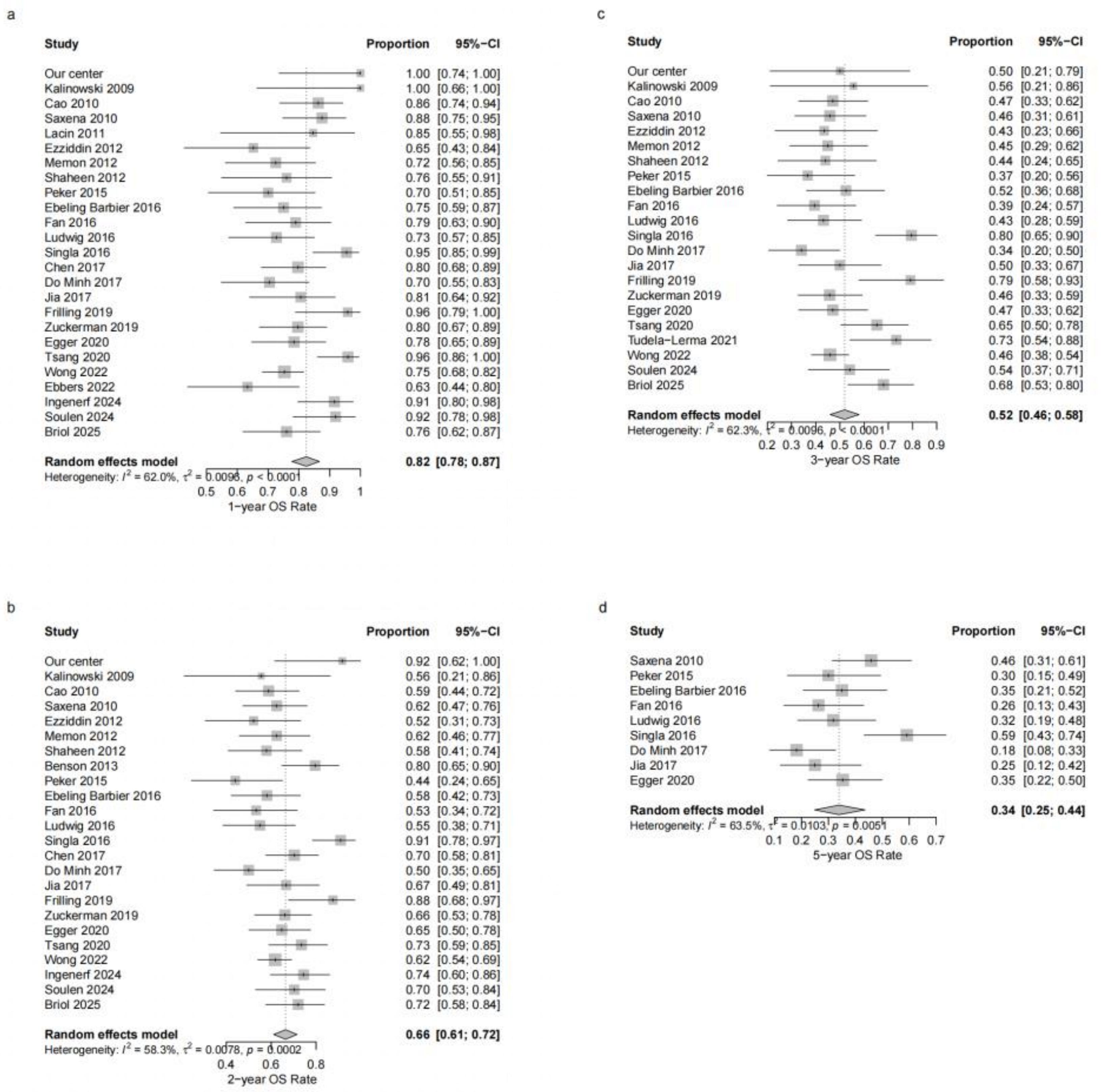

Supplementary Figure S3. Forest plots of 1-, 2-, 3-, and 5-year overall survival (OS) rates

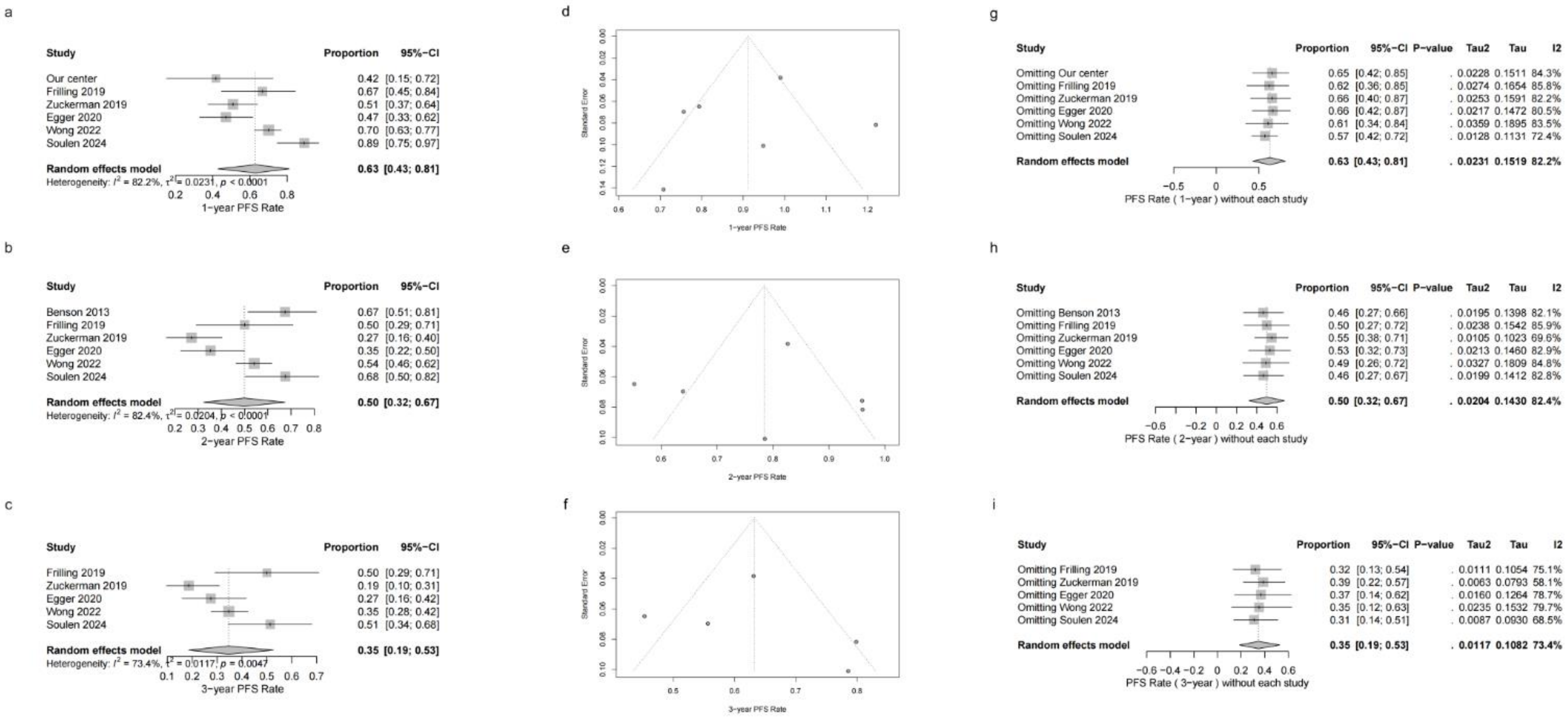

Supplementary Figure S4. Forest plots funnel plots, and leave-one-out analyses at 1-, 2-, and 3-year progression-free survival (PFS) rates

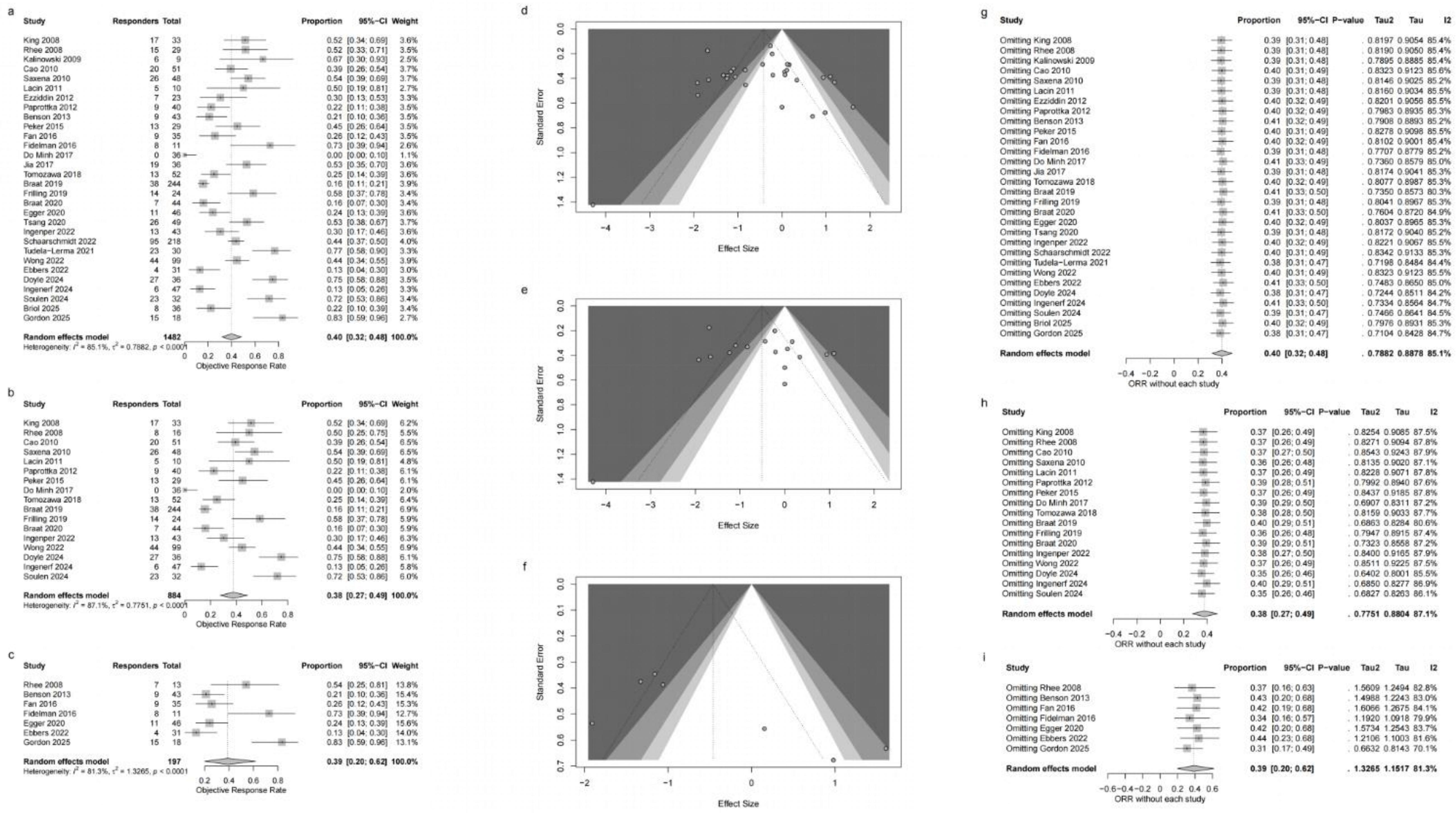

Supplementary Figure S5. Forest plots (a–c), funnel plots (d–f), and leave-one-out sensitivity analyses (g–i) for objective response rate (ORR) based on RECIST criteria

Panels represent the total study group (a, d, g), resin microsphere subgroup (b, e, h), and glass microsphere subgroup (c, f, i).

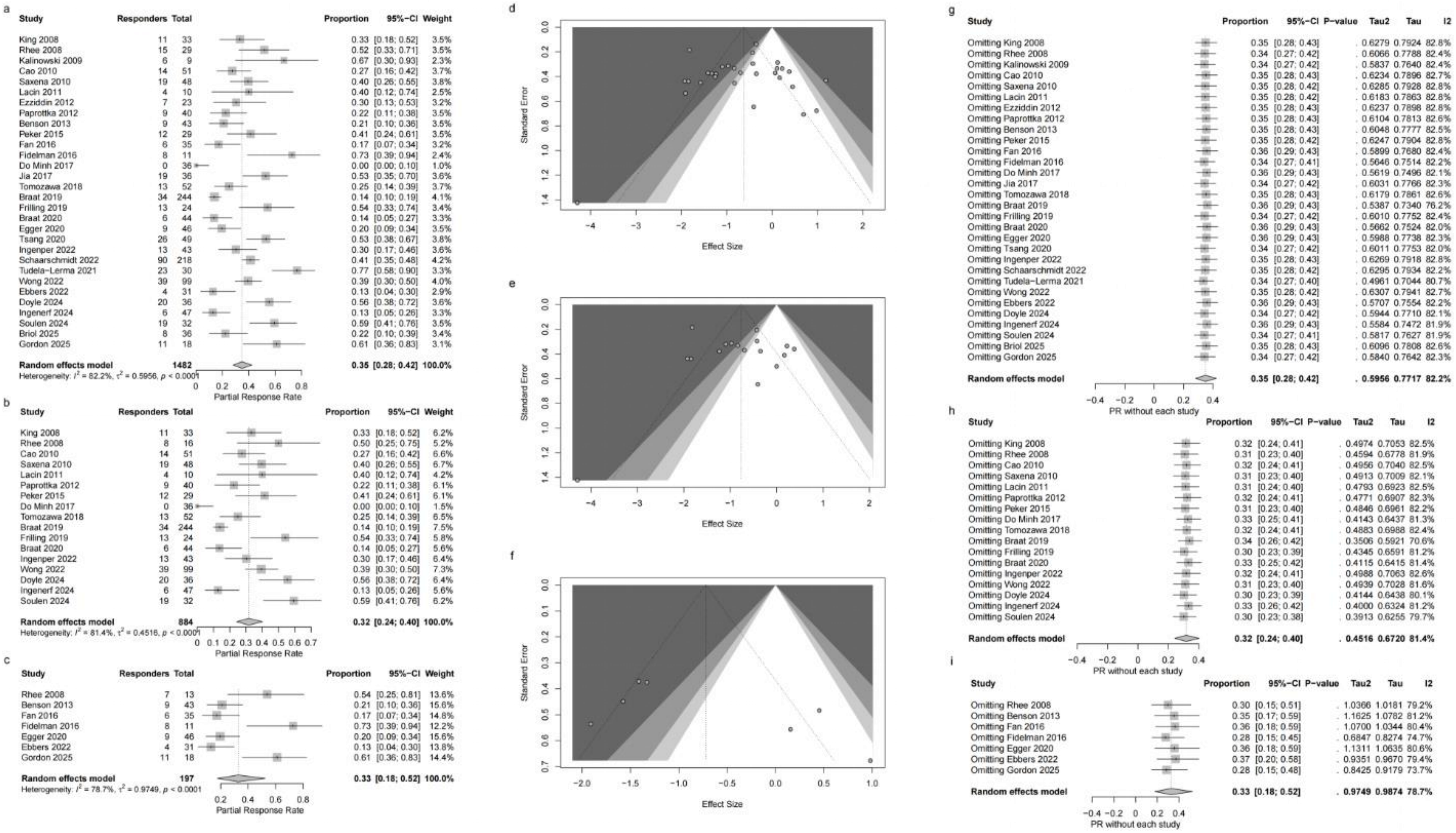

Supplementary Figure S6. Forest plots (a–c), funnel plots (d–f), and leave-one-out sensitivity analyses (g–i) for partial response (PR) rate based on RECIST criteria

Panels represent the total study group (a, d, g), resin microsphere subgroup (b, e, h), and glass microsphere subgroup (c, f, i).

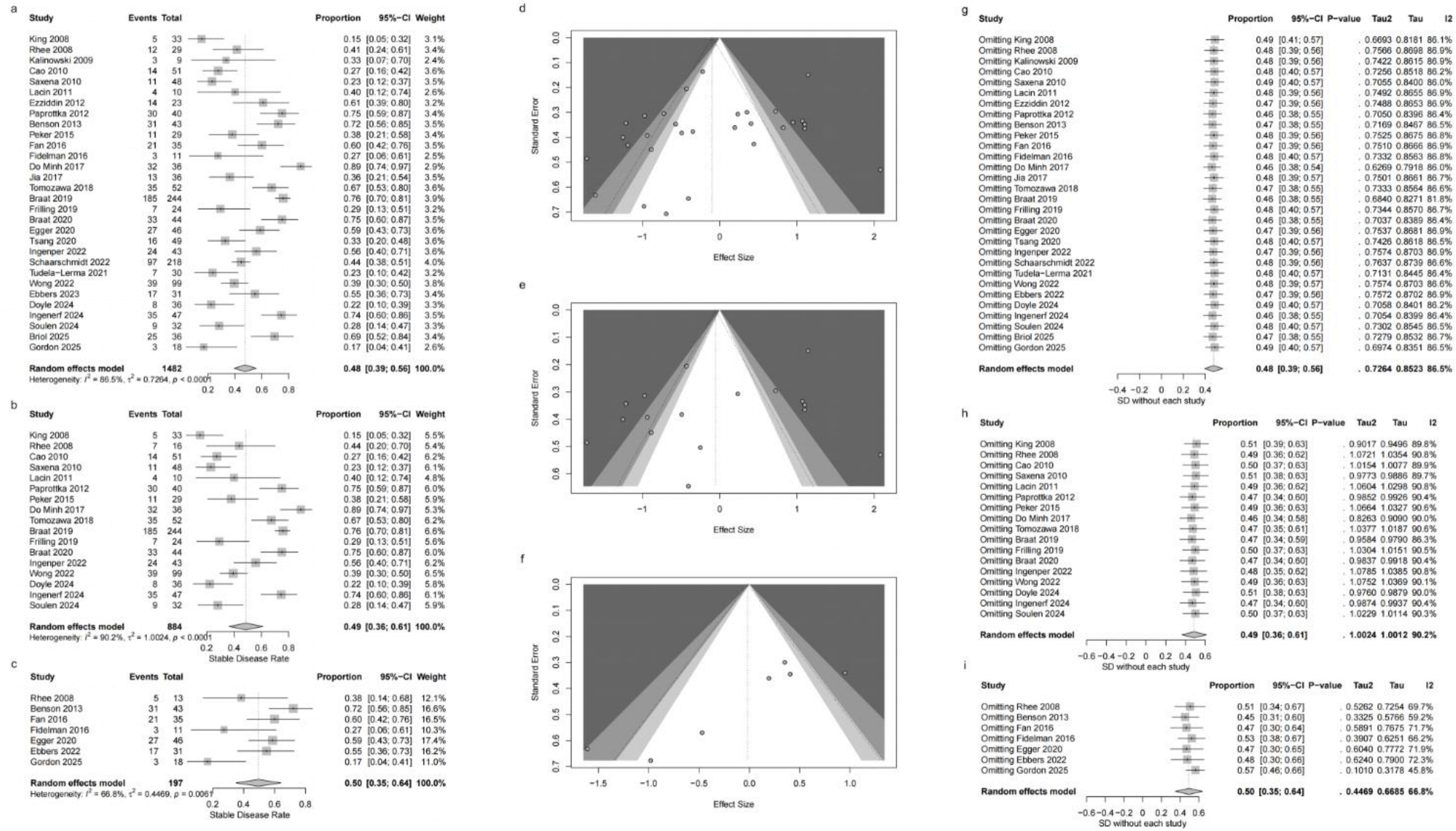

Supplementary Figure S7. Forest plots (a–c), funnel plots (d–f), and leave-one-out sensitivity analyses (g–i) for stable disease (SD) rate based on RECIST criteria

Panels represent the total study group (a, d, g), resin microsphere subgroup (b, e, h), and glass microsphere subgroup (c, f, i).

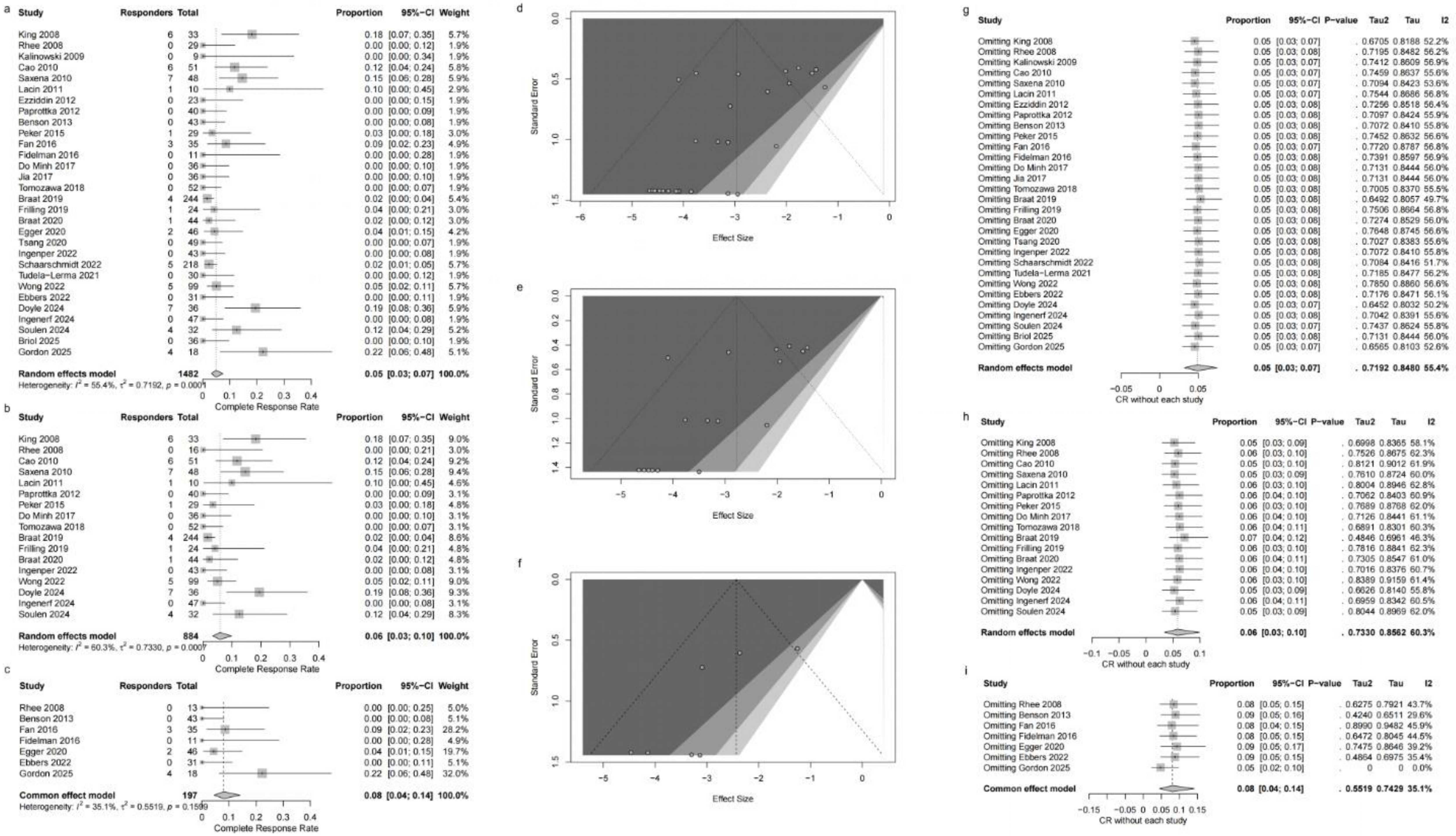

Supplementary Figure S8. Forest plots (a–c), funnel plots (d–f), and leave-one-out sensitivity analyses (g–i) for complete response (CR) rate based on RECIST criteria

Panels represent the total study group (a, d, g), resin microsphere subgroup (b, e, h), and glass microsphere subgroup (c, f, i).

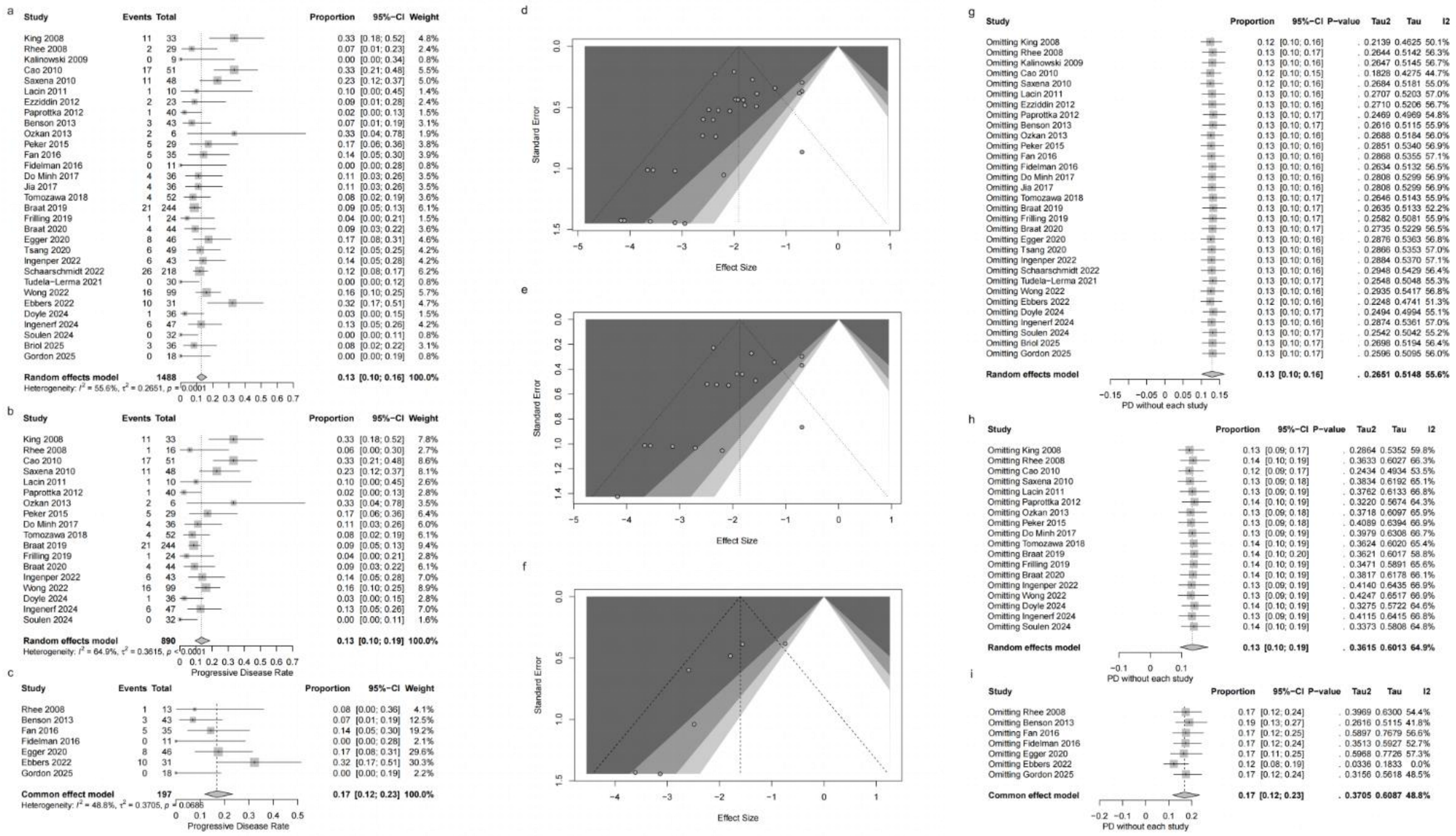

Supplementary Figure S9. Forest plots (a–c), funnel plots (d–f), and leave-one-out sensitivity analyses (g–i) for progressive disease (PD) rate based on RECIST criteria

Panels represent the total study group (a, d, g), resin microsphere subgroup (b, e, h), and glass microsphere subgroup (c, f, i).

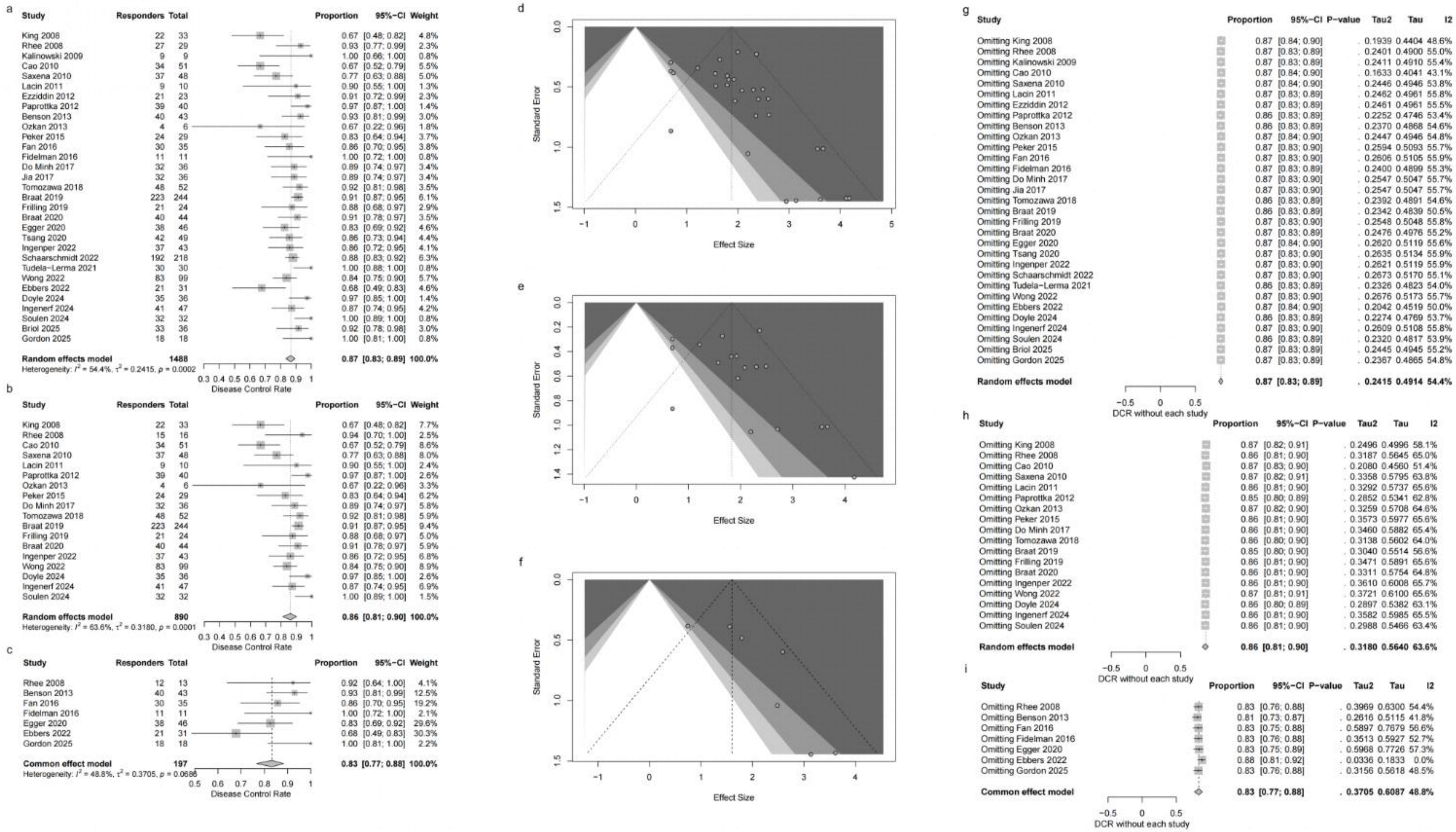

Supplementary Figure S10. Forest plots (a–c), funnel plots (d–f), and leave-one-out sensitivity analyses (g–i) for disease control rate (DCR) based on RECIST criteria

Panels represent the total study group (a, d, g), resin microsphere subgroup (b, e, h), and glass microsphere subgroup (c, f, i).

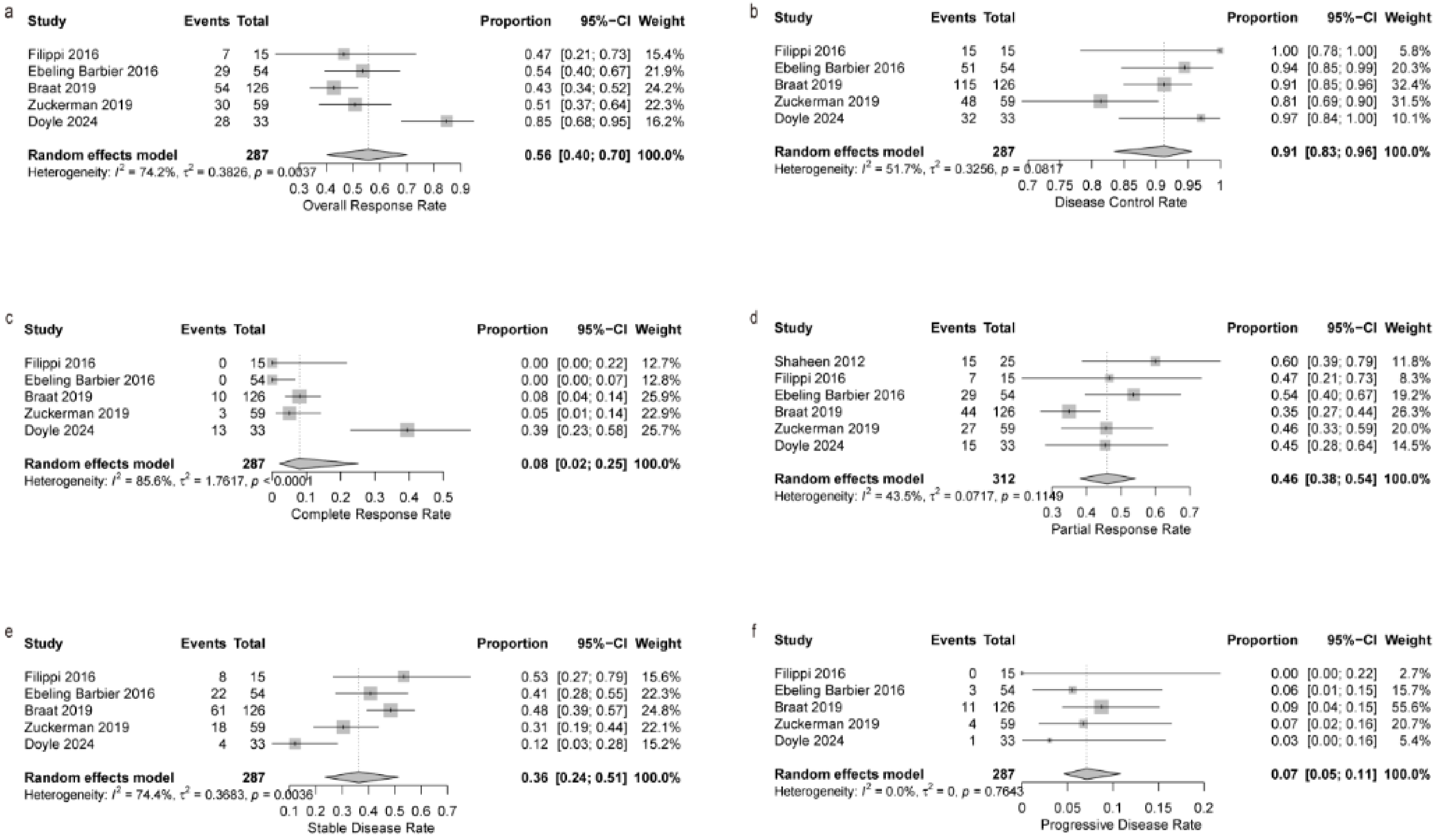

Supplementary Figure S11. Forest plots showing pooled proportions of tumor response outcomes assessed by mRECIST criteria

(a) objective response rate, (b) disease control rate, (c) complete response, (d) partial response, (e) stable disease, and (f) progressive disease

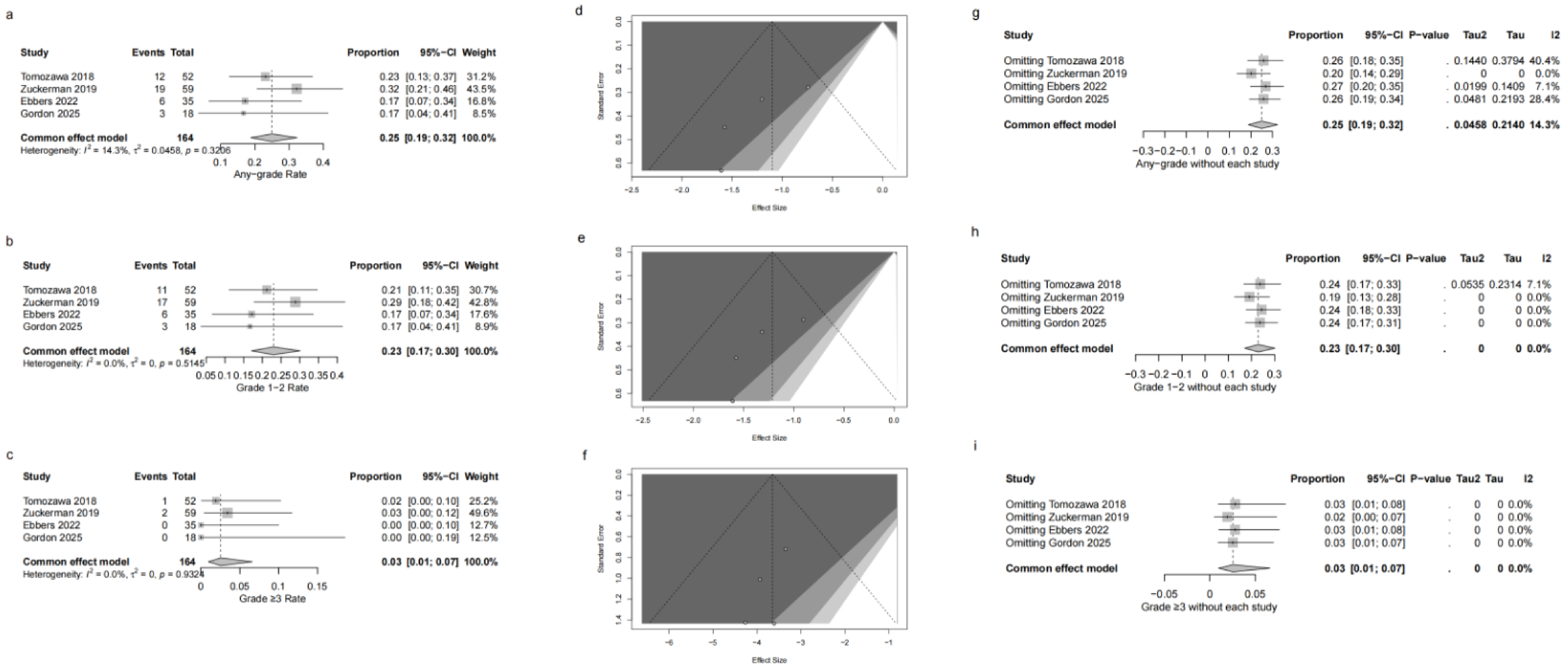

Supplementary Figure S12. Forest plots (a–c), funnel plots (d–f), and leave-one-out sensitivity analyses (g–i) for adverse events related to ALB decrease

(a, d, g) any-grade events, (b, e, h) grade 1–2 events, and (c, f, i) grade ≥3 events

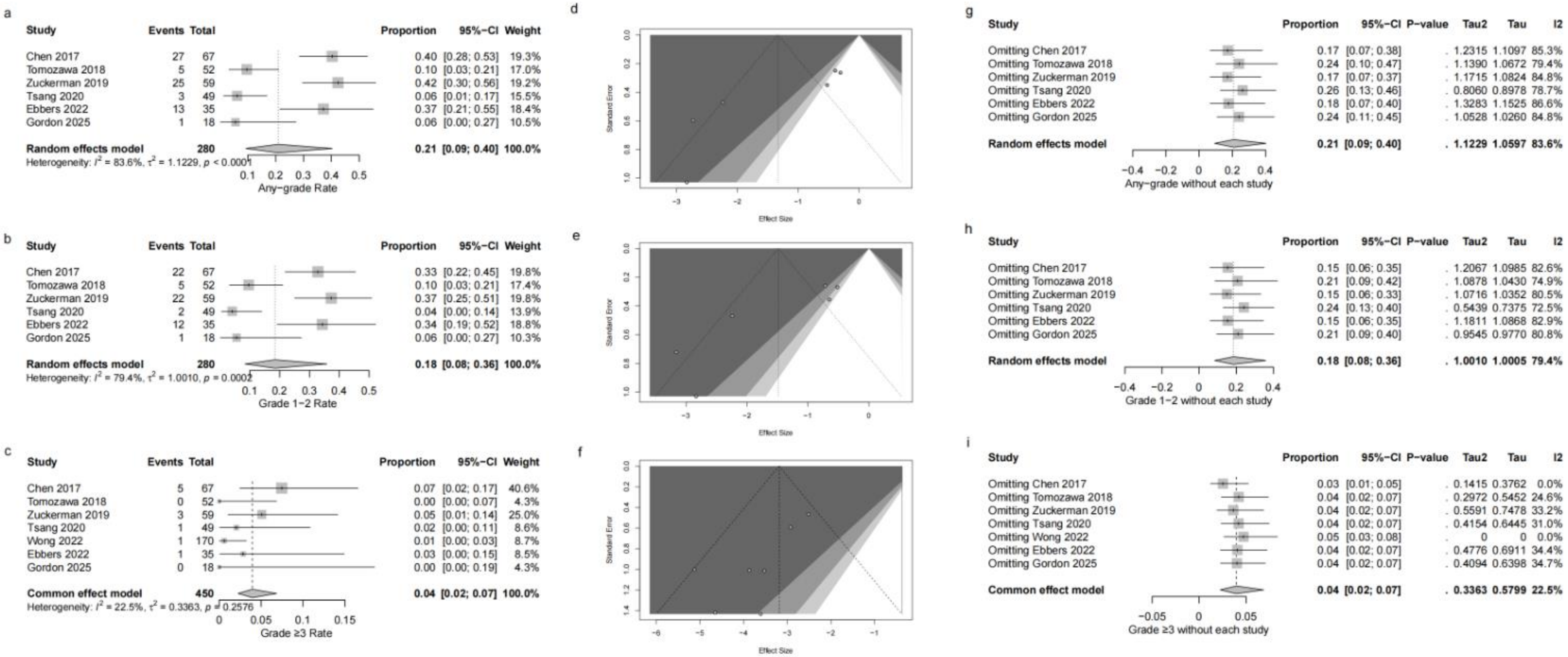

Supplementary Figure S13. Forest plots (a–c), funnel plots (d–f), and leave-one-out sensitivity analyses (g–i) for adverse events related to ALT elevation

(a, d, g) any-grade events, (b, e, h) grade 1–2 events, and (c, f, i) grade ≥3 events

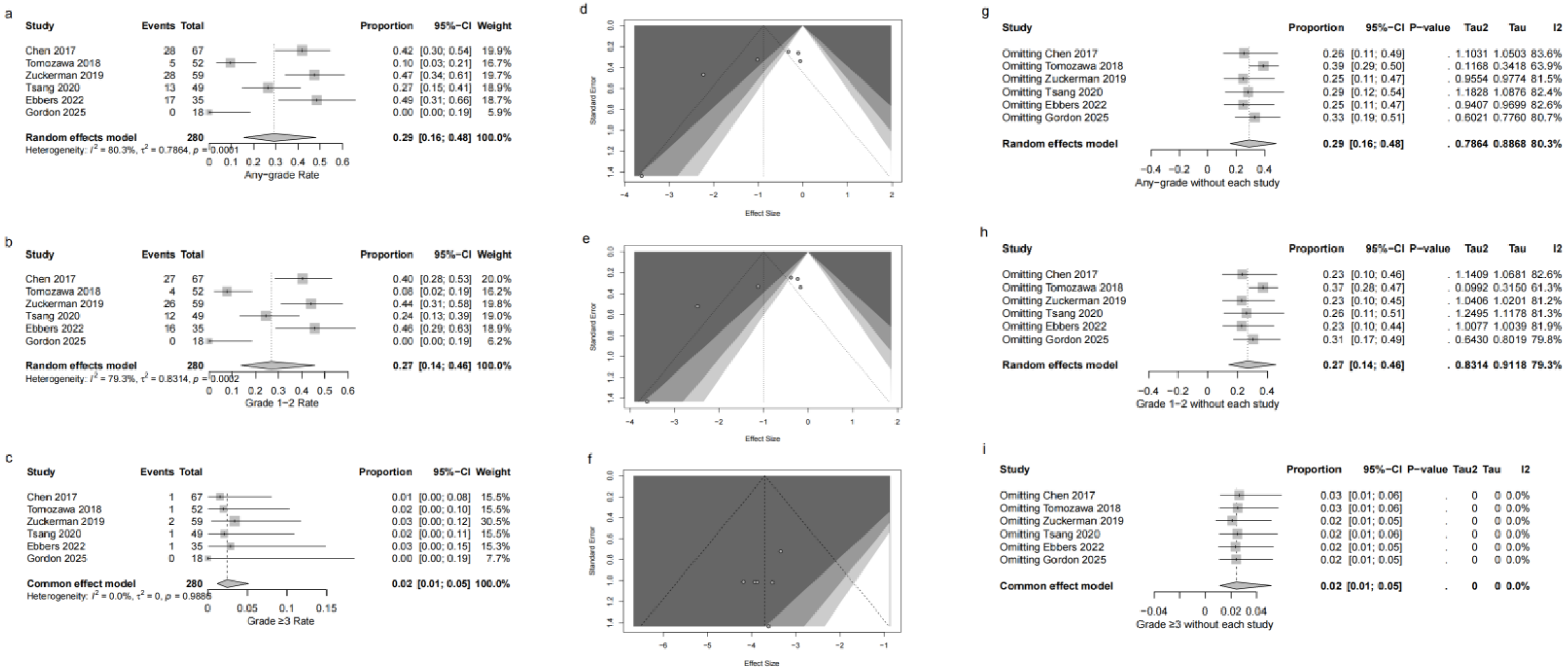

Supplementary Figure S14. Forest plots (a–c), funnel plots (d–f), and leave-one-out sensitivity analyses (g–i) for adverse events related to AST elevation

(a, d, g) any-grade events, (b, e, h) grade 1–2 events, and (c, f, i) grade ≥3 events

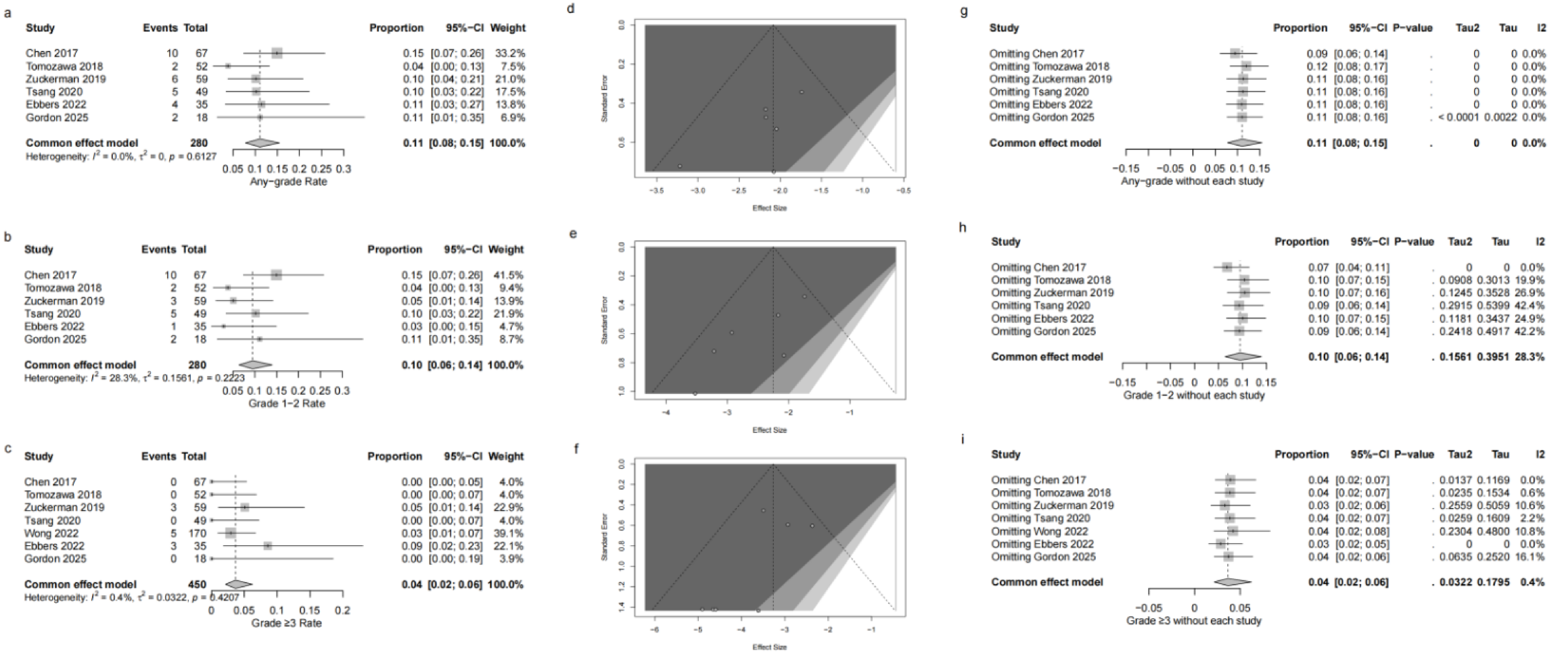

Supplementary Figure S15. Forest plots (a–c), funnel plots (d–f), and leave-one-out sensitivity analyses (g–i) for adverse events related to bilirubin elevation

(a, d, g) any-grade events, (b, e, h) grade 1–2 events, and (c, f, i) grade ≥3 events

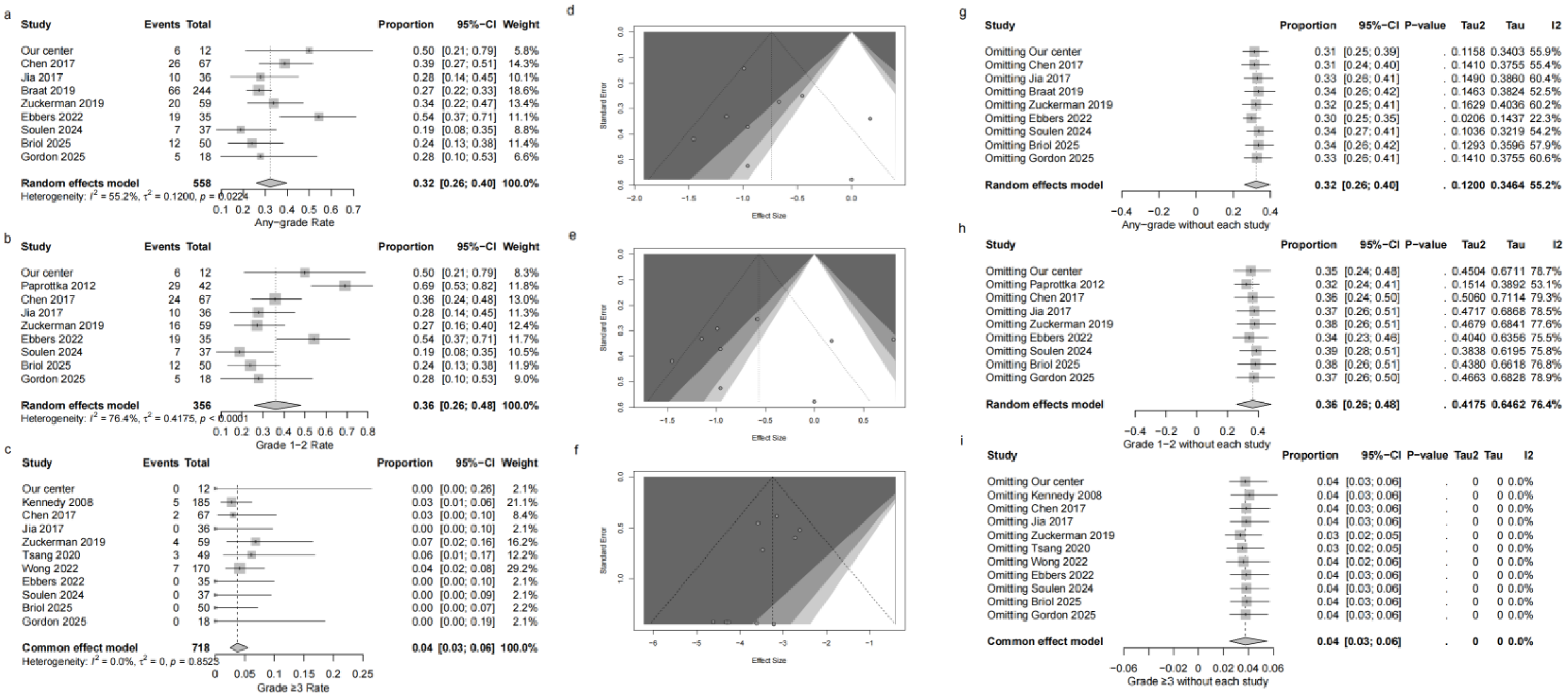

Supplementary Figure S16. Forest plots (a–c), funnel plots (d–f), and leave-one-out sensitivity analyses (g–i) for adverse events related to abdominal pain

(a, d, g) any-grade events, (b, e, h) grade 1–2 events, and (c, f, i) grade ≥3 events

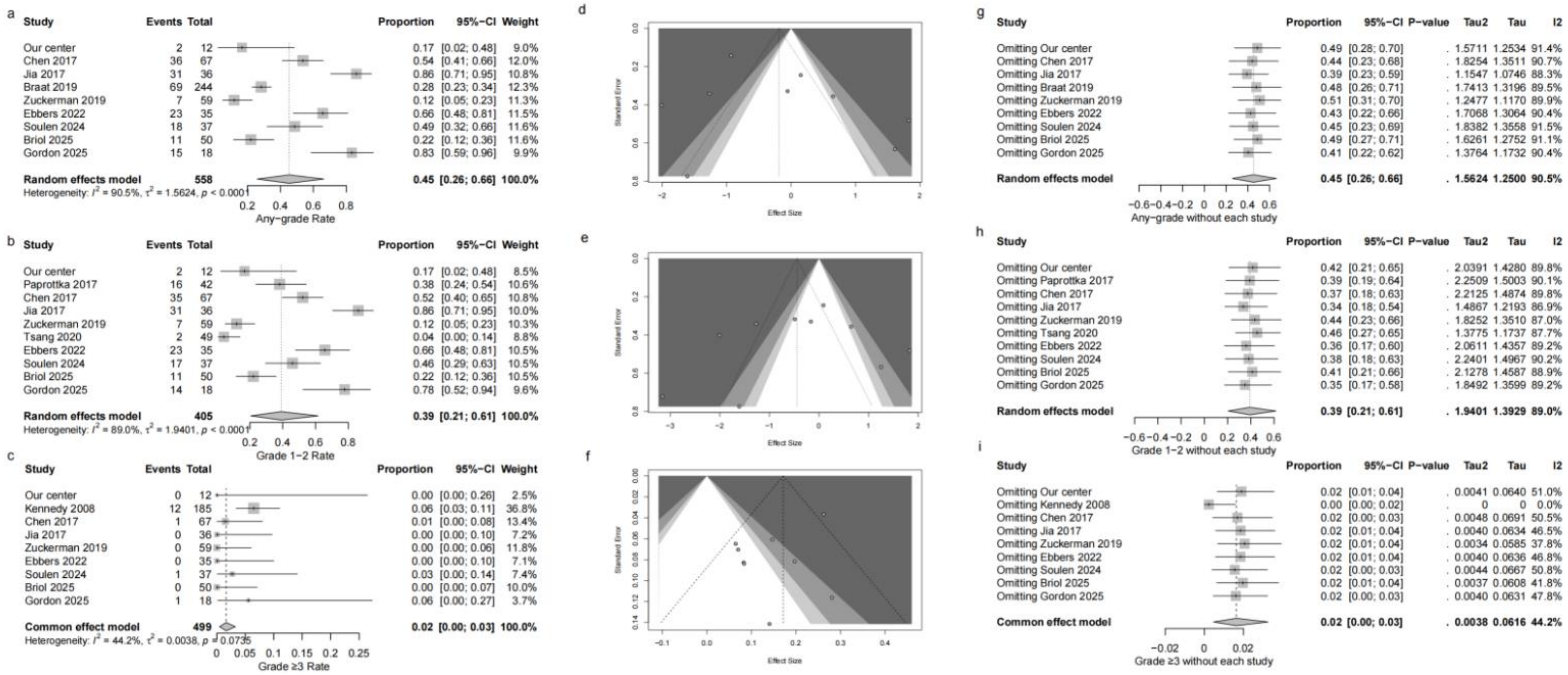

Supplementary Figure S17. Forest plots (a–c), funnel plots (d–f), and leave-one-out sensitivity analyses (g–i) for adverse events related to fatigue

(a, d, g) any-grade events, (b, e, h) grade 1–2 events, and (c, f, i) grade ≥3 events

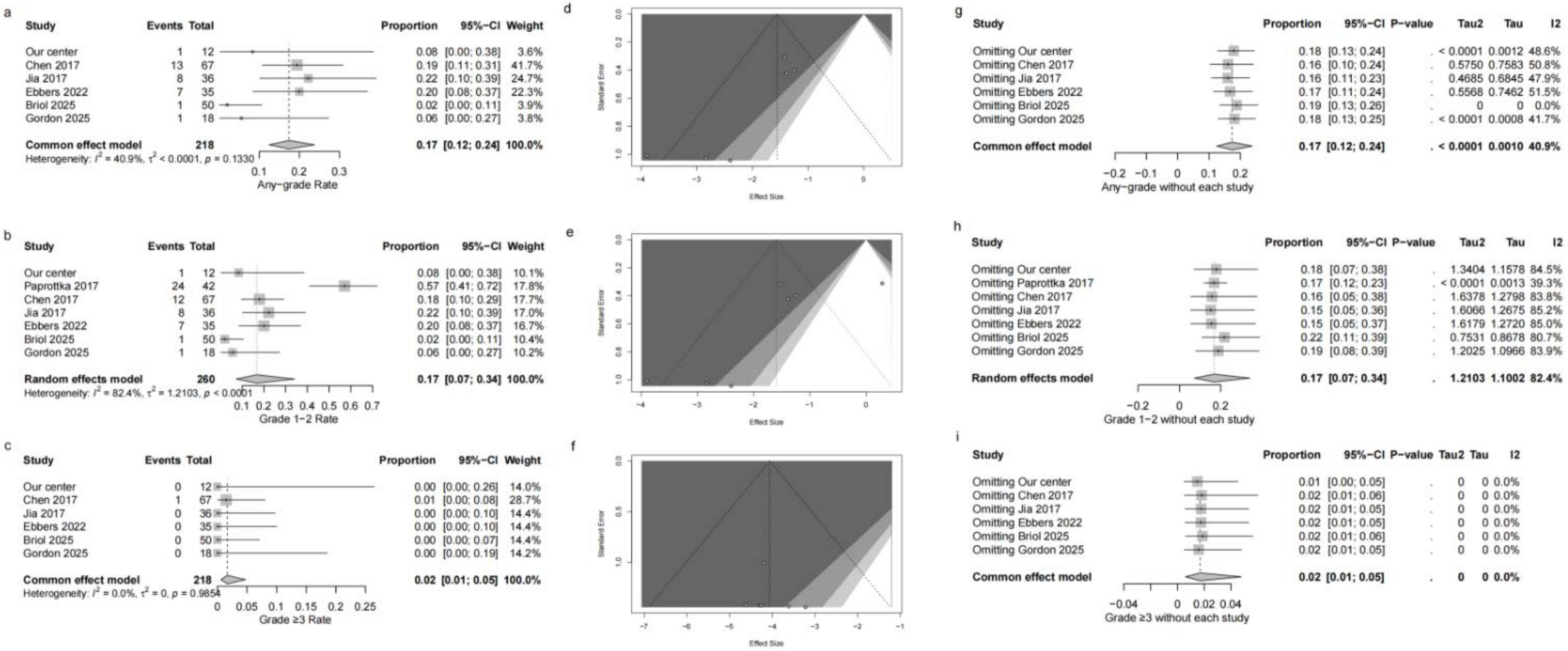

Supplementary Figure S18. Forest plots (a–c), funnel plots (d–f), and leave-one-out sensitivity analyses (g–i) for adverse events related to fever

(a, d, g) any-grade events, (b, e, h) grade 1–2 events, and (c, f, i) grade ≥3 events

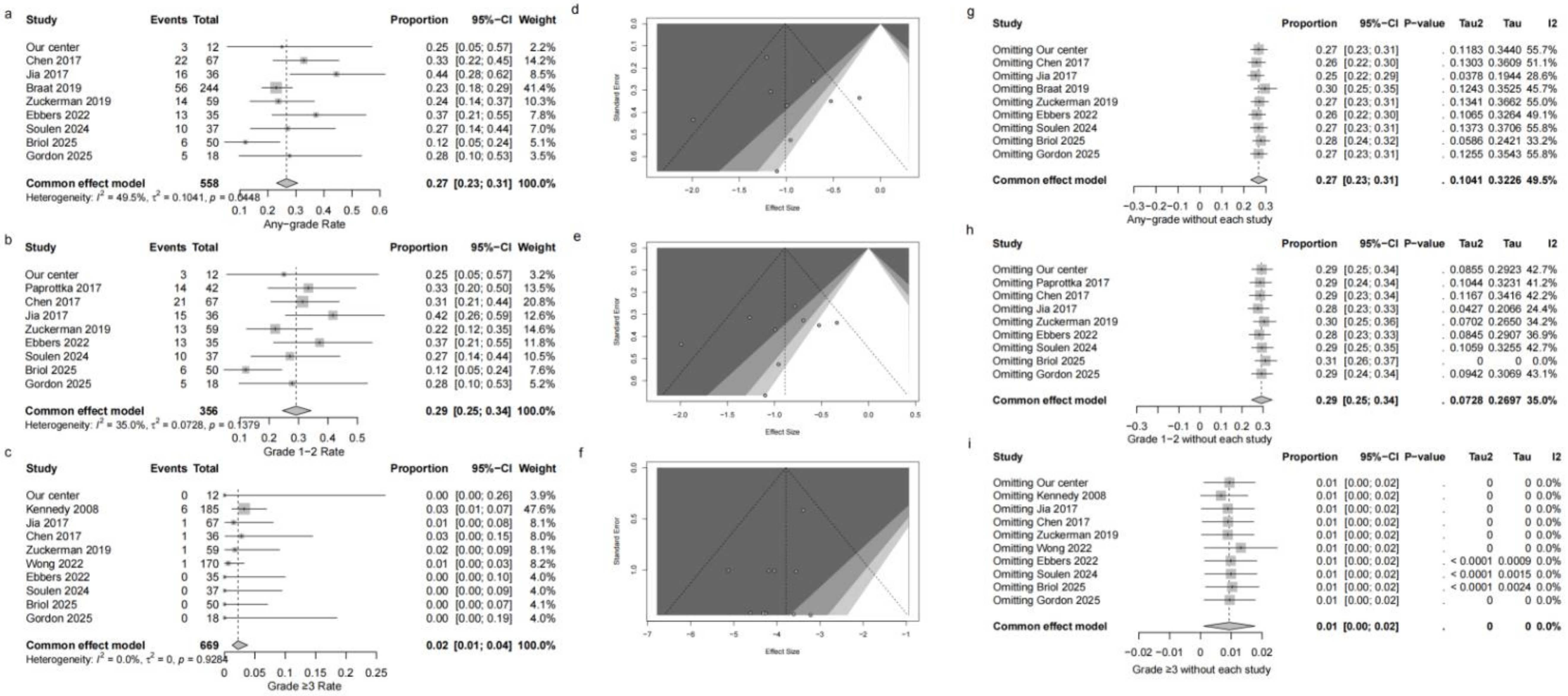

Supplementary Figure S19. Forest plots (a–c), funnel plots (d–f), and leave-one-out sensitivity analyses (g–i) for adverse events related to nausea

(a, d, g) any-grade events, (b, e, h) grade 1–2 events, and (c, f, i) grade ≥3 events

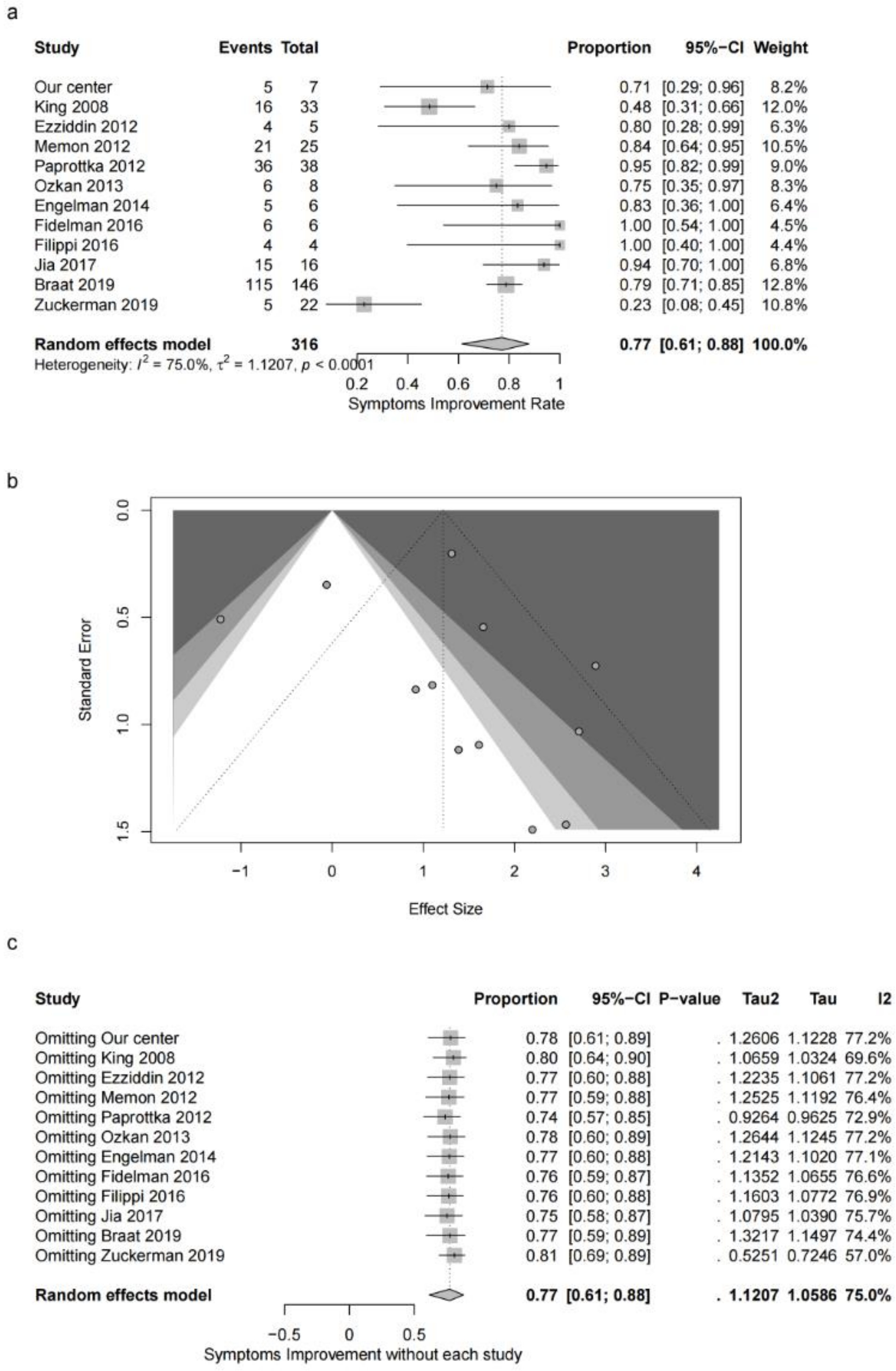

Supplementary Figure S20. Forest plot (a), funnel plot (b), and leave-one-out sensitivity analysis (c) for symptom improvement rate

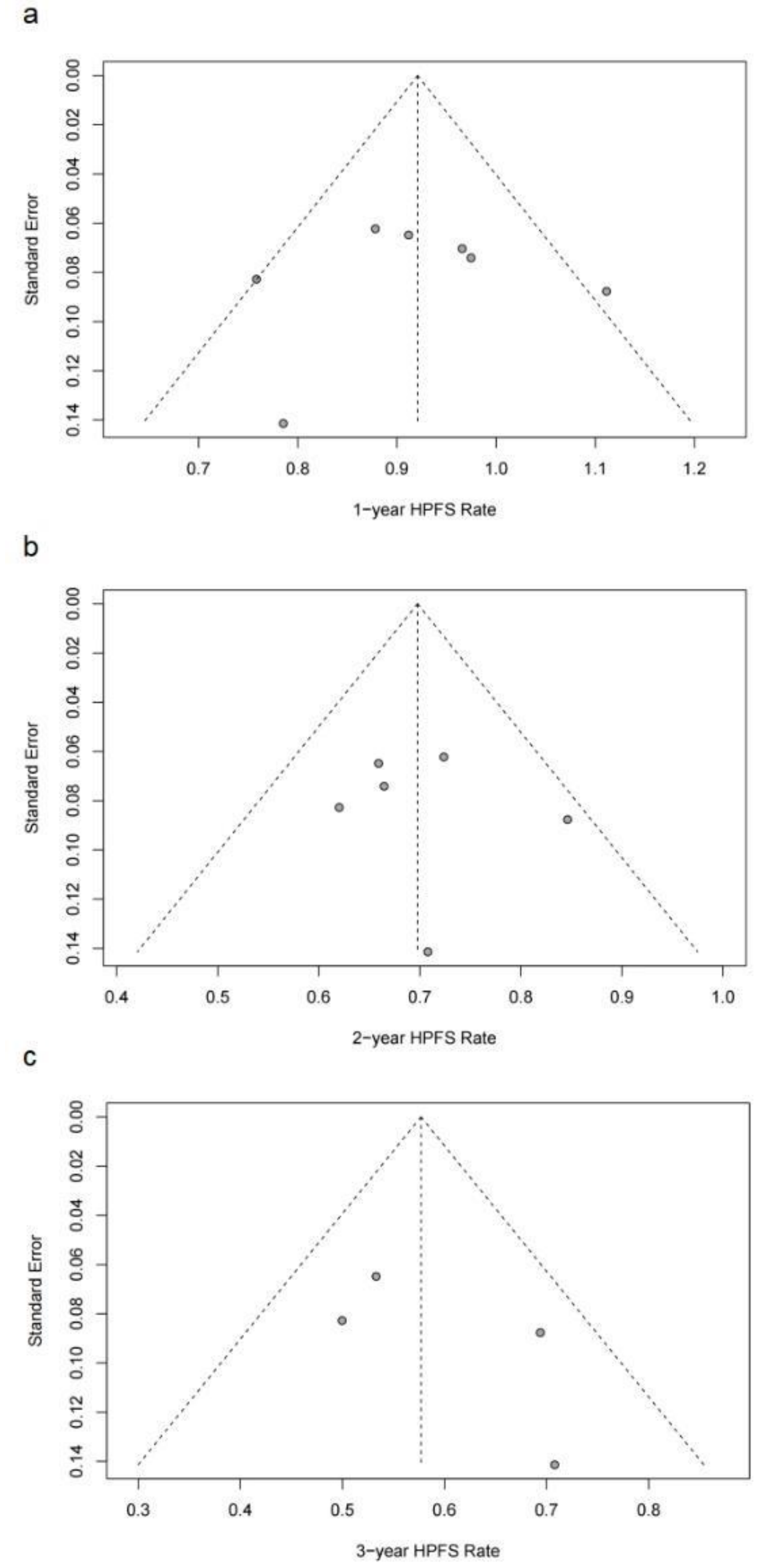

Supplementary Figure S21. Funnel plots for hepatic progression-free survival (HPFS) rates

(a) 1-year HPFS, (b) 2-year HPFS, and (c) 3-year HPFS

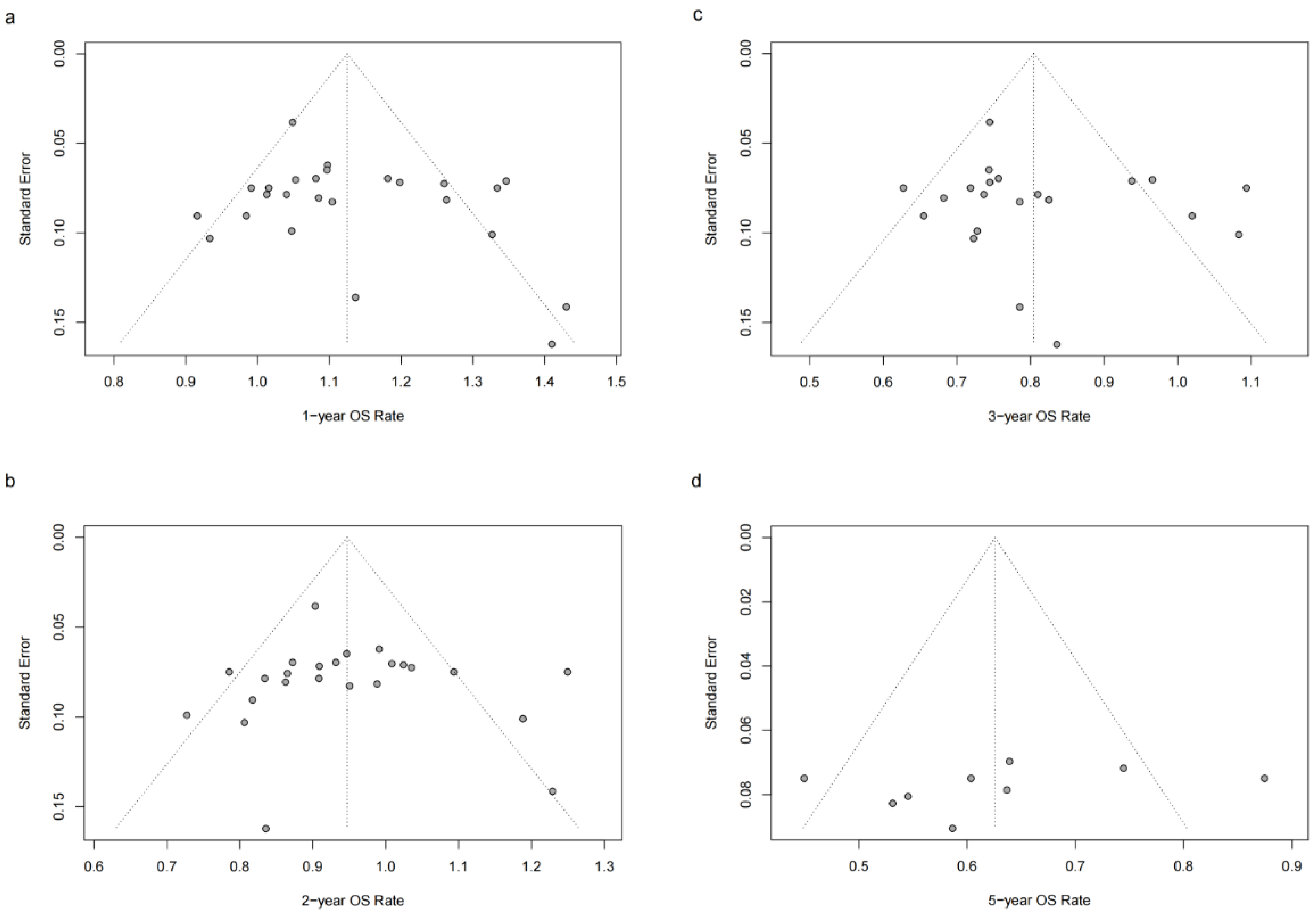

Supplementary Figure S22. Funnel plots for overall survival (OS) rates

(a) 1-year OS, (b) 2-year OS, (c) 3-year OS, and (d) 5-year OS

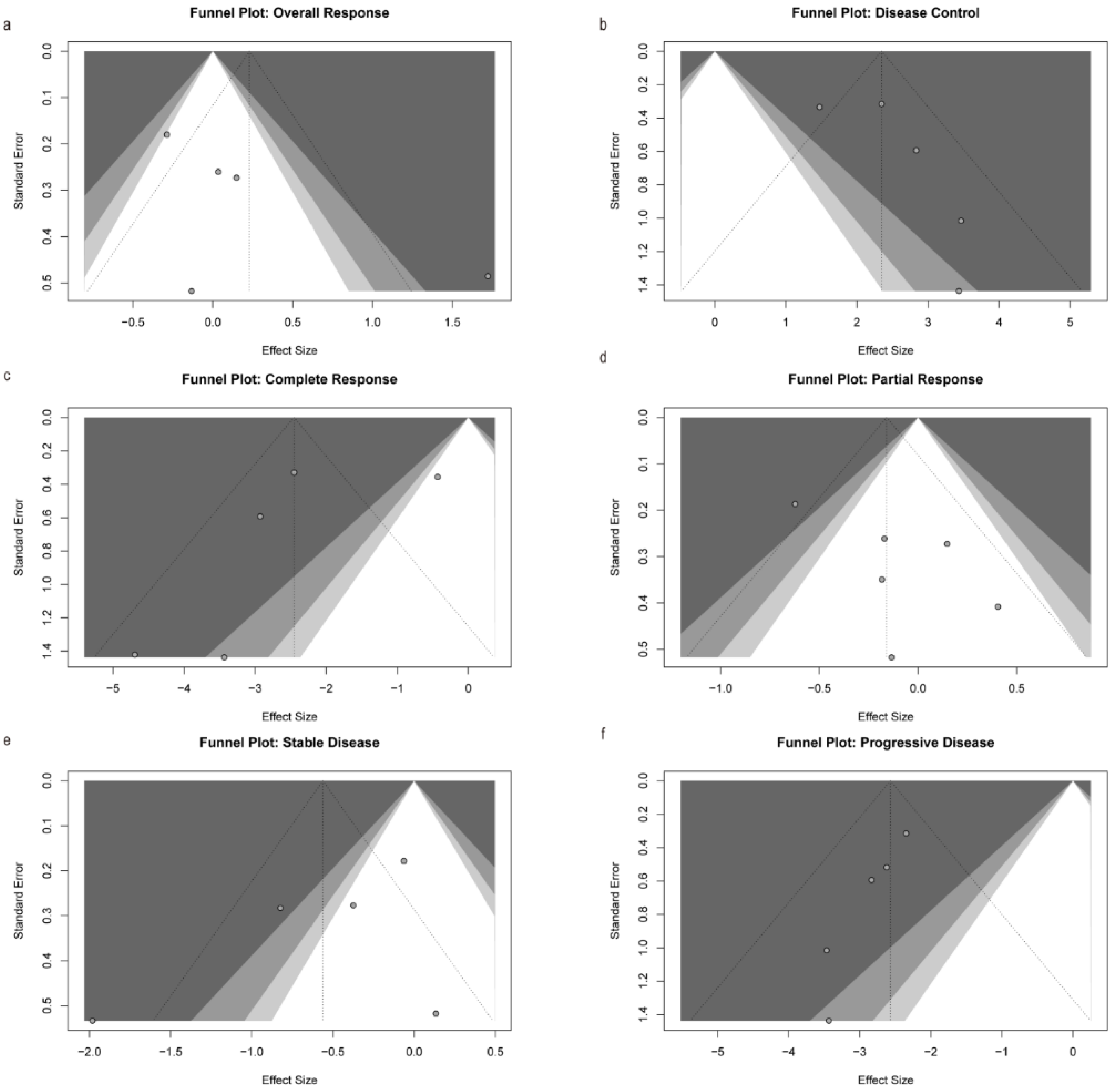

Supplementary Figure S23. Funnel plots for tumor response outcomes assessed by mRECIST criteria

(a) objective response rate, (b) disease control rate, (c) complete response, (d) partial response, (e) stable disease, and (f) progressive disease

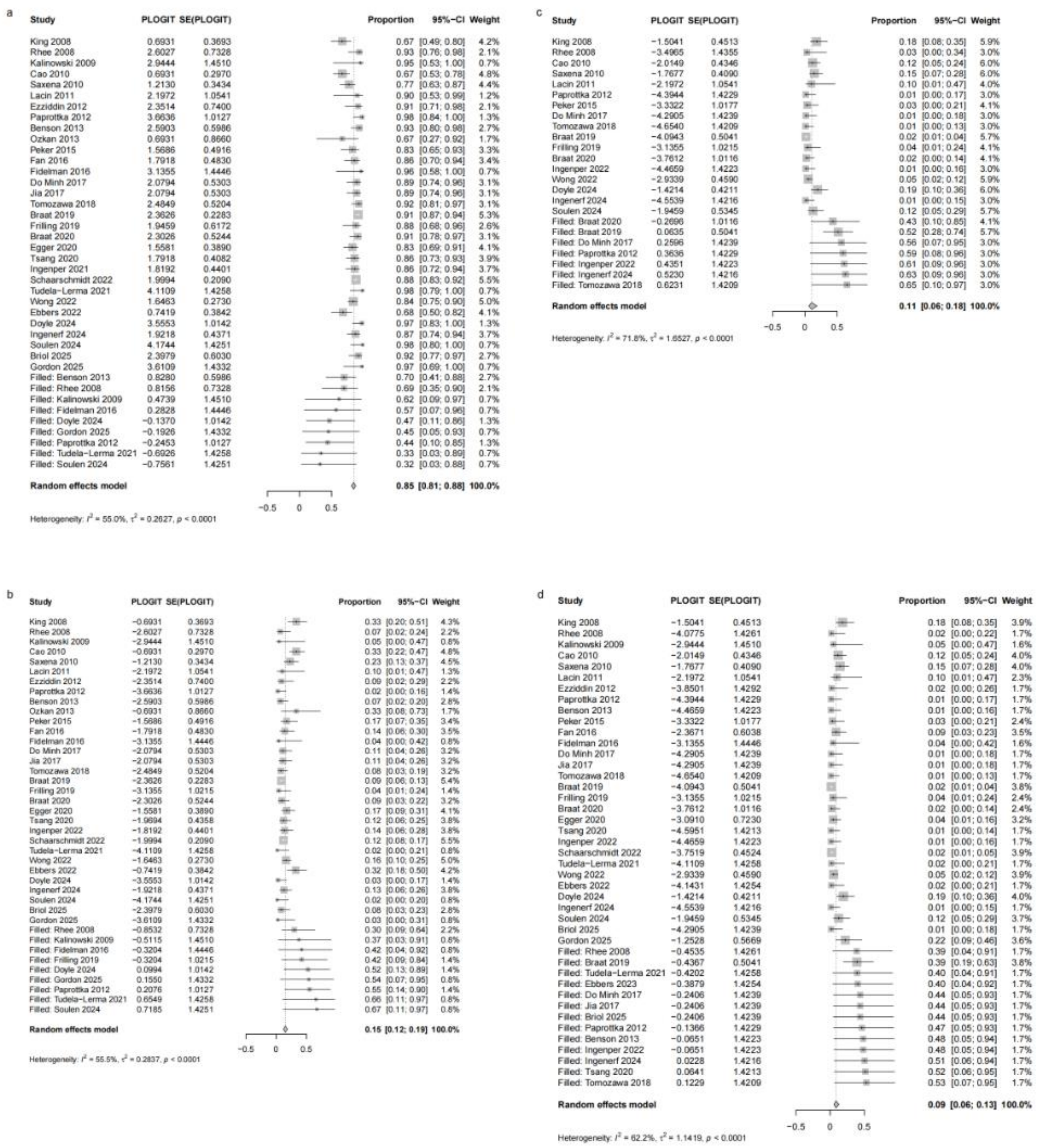

Supplementary Figure S24. Trim-and-fill analyses for tumor response outcomes assessed by mRECIST criteria

(a) disease control rate, (b) complete response, (c) complete response in the resin microsphere subgroup, and (d) progressive disease

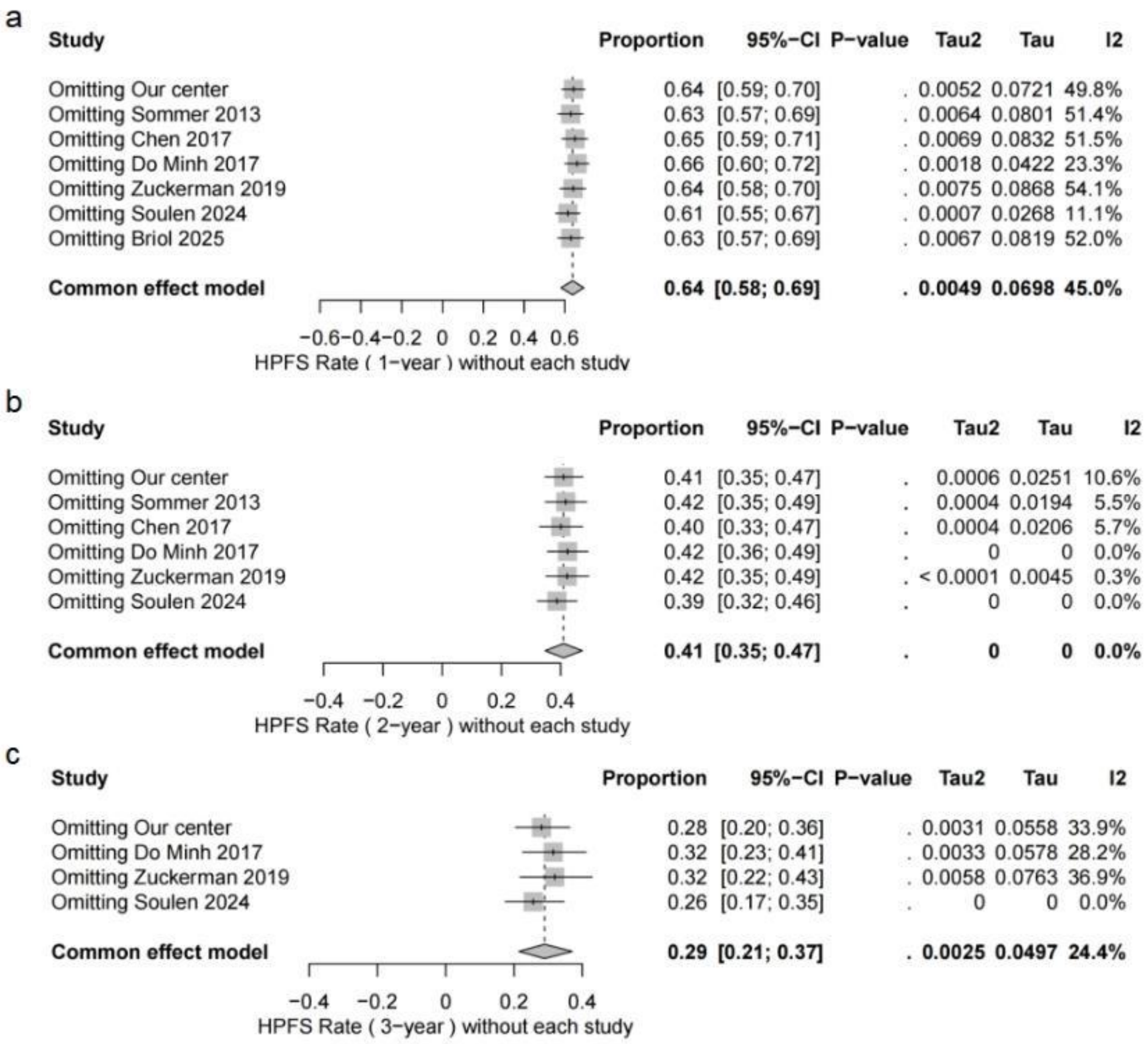

Supplementary Figure S25. Leave-one-out sensitivity analyses for hepatic progression-free survival (HPFS)

(a) 1-year HPFS rate, (b) 2-year HPFS rate, and (c) 3-year HPFS rate

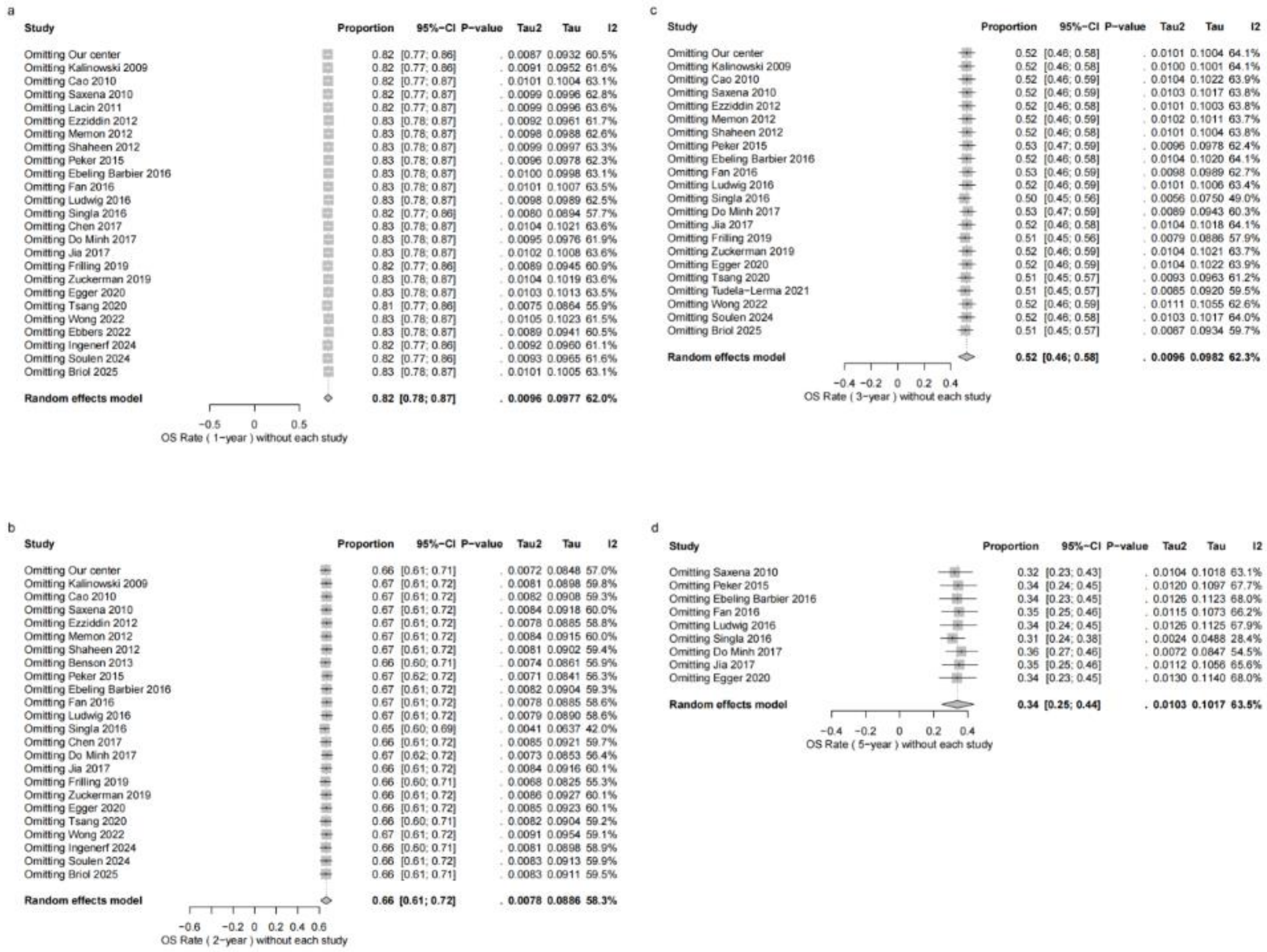

Supplementary Figure S26. Leave-one-out sensitivity analyses for overall survival (OS)

(a) 1-year OS rate, (b) 2-year OS rate, (c) 3-year OS rate, and (d) 5-year OS rate

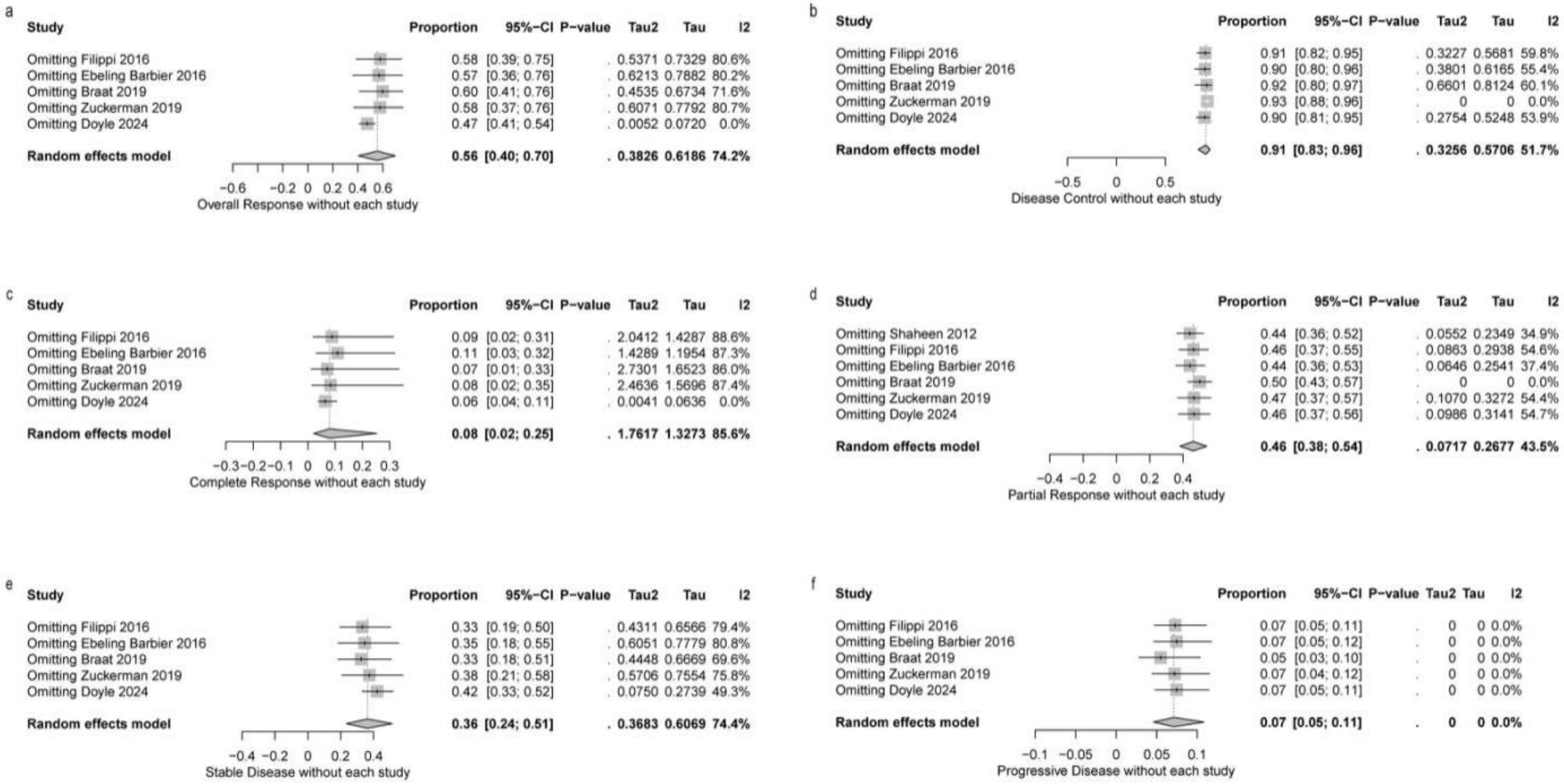

Supplementary Figure S27. Leave-one-out sensitivity analyses for tumor response outcomes assessed by mRECIST criteria

(a) objective response rate, (b) disease control rate, (c) complete response, (d) partial response, (e) stable disease, and (f) progressive disease
